# Supplementary figures and images for: Elimination of subtelomeric repeat sequences exerts little effect on telomere essential functions in Saccharomyces cerevisiae
Source: eLife. 2024 Apr 24;12:RP91223. doi: 10.7554/eLife.91223 (PMC11042809; doi:10.7554/eLife.91223)

**A**

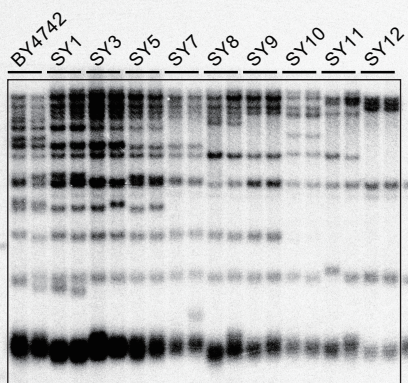

Supplement: Figure 1—source data 2. [file elife-91223-fig1-data2.zip › PDF containing Figure 1A and original scans of the relevant Southern blot analysis.pdf]

B

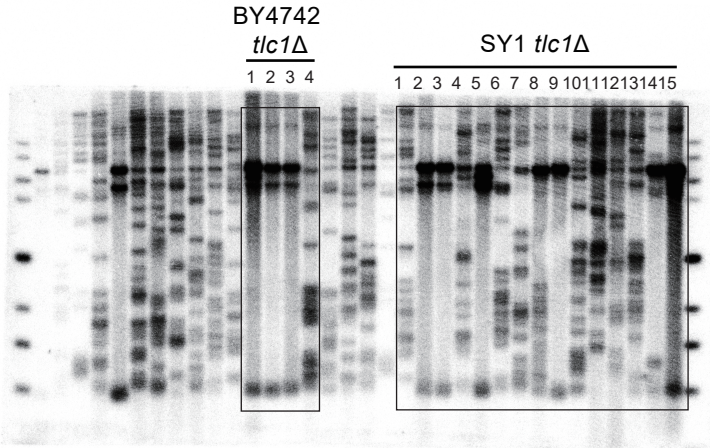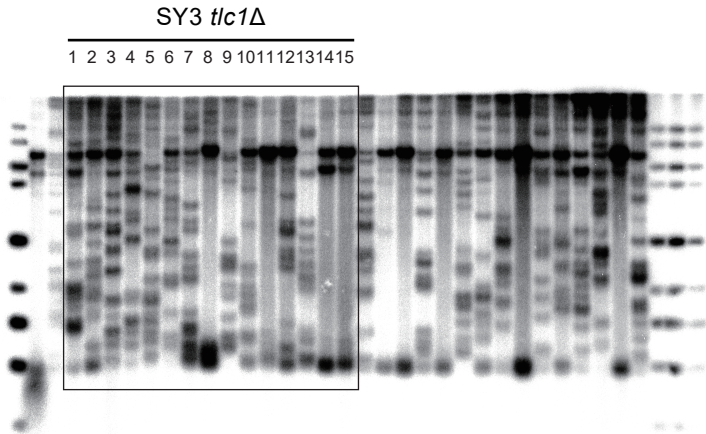

Supplement: Figure 1—source data 5. [file elife-91223-fig1-data5.zip › PDF containing Figure 1B and original scans of the relevant Southern blot analysis.pdf]

C

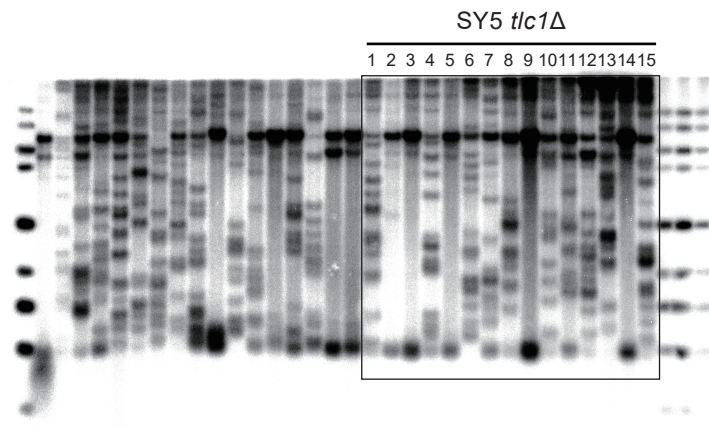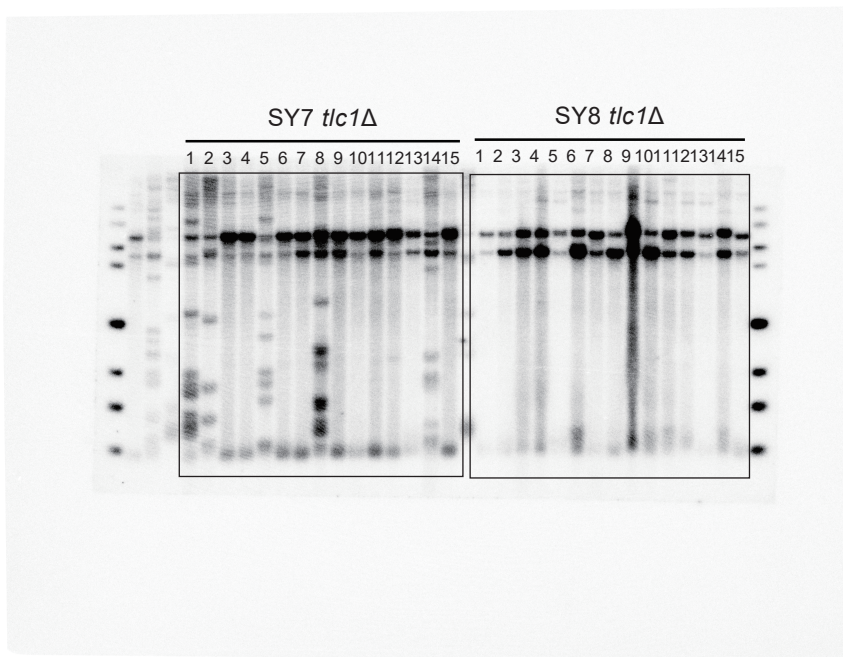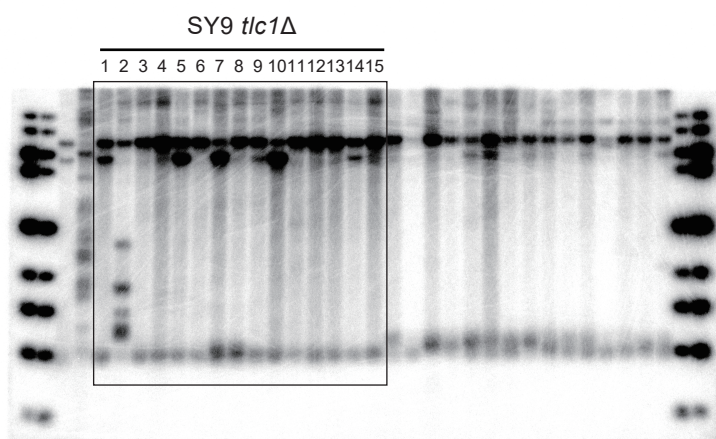

Supplement: Figure 1—source data 9. [file elife-91223-fig1-data9.zip › PDF containing Figure 1C and original scans of the relevant Southern blot analysis.pdf]

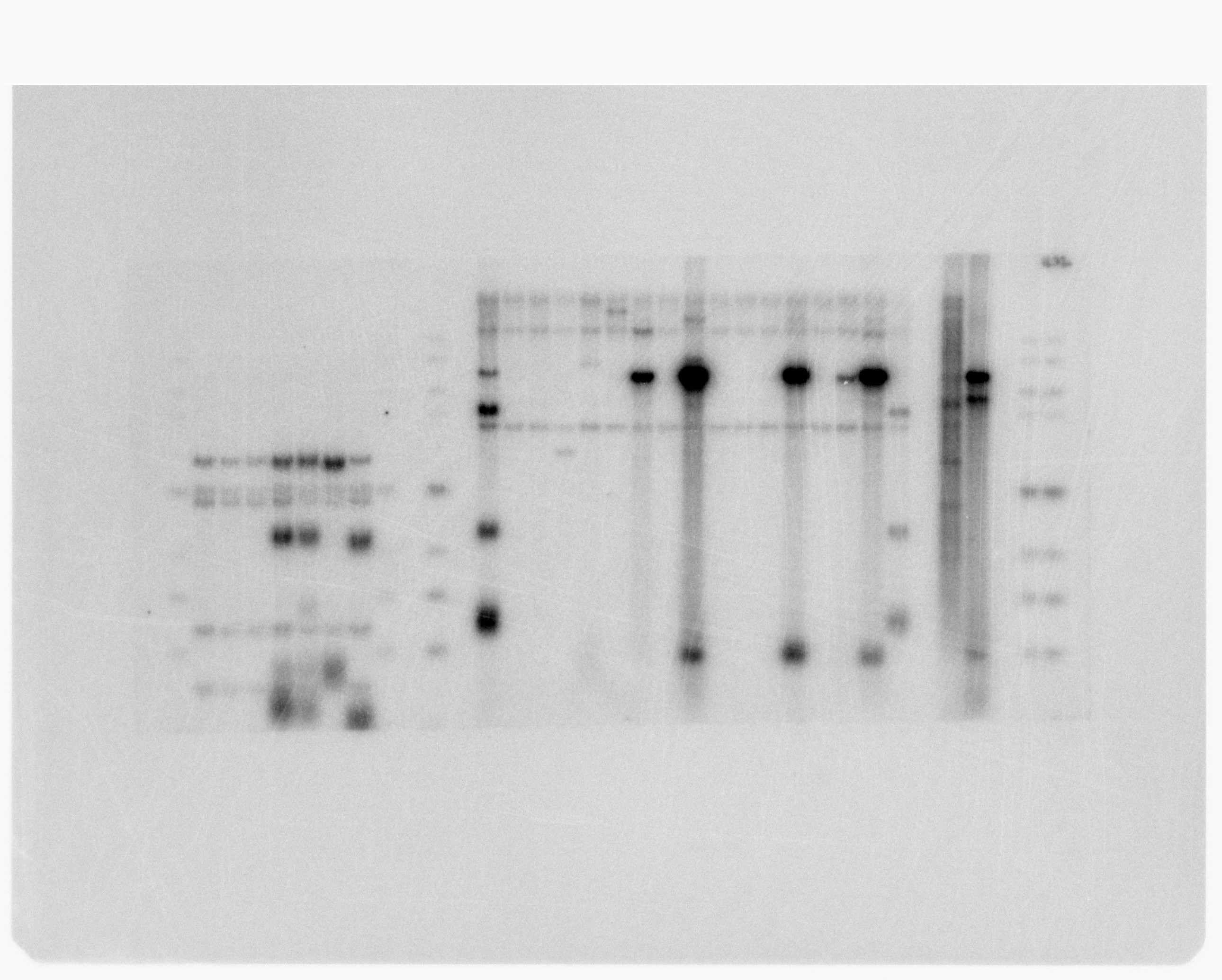

Supplement: Figure 1—source data 13. [file elife-91223-fig1-data13.zip › Figure1D source data4.tif]

D

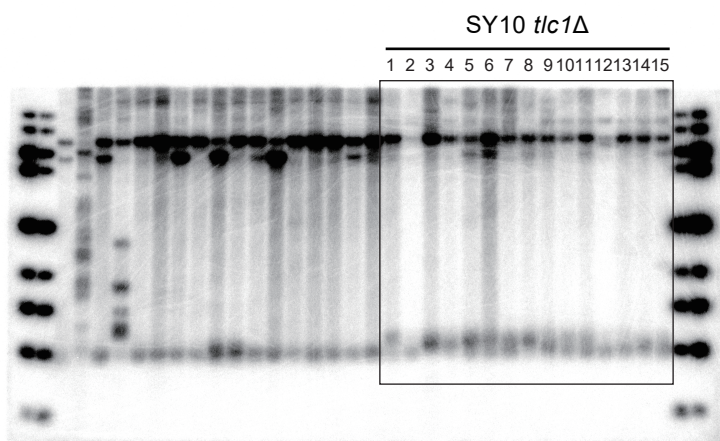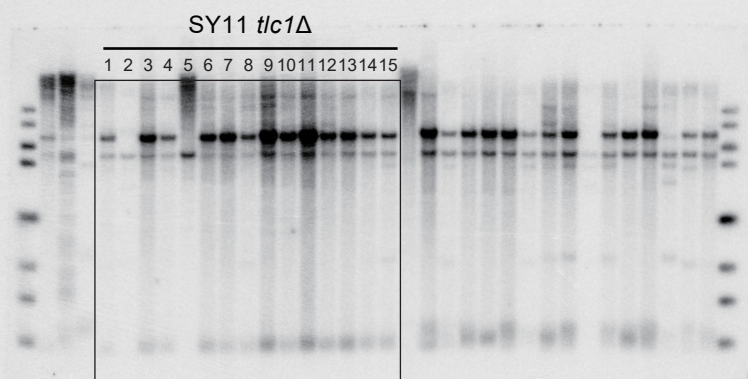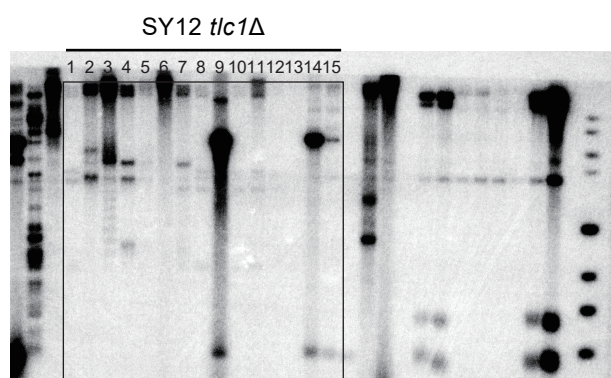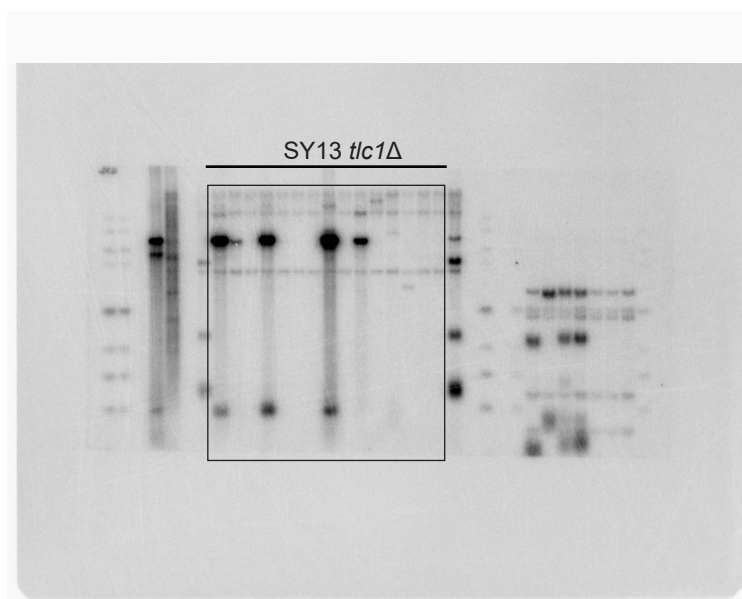

Supplement: Figure 1—source data 14. [file elife-91223-fig1-data14.zip › PDF containing Figure 1D and original scans of the relevant Southern blot analysis.pdf]

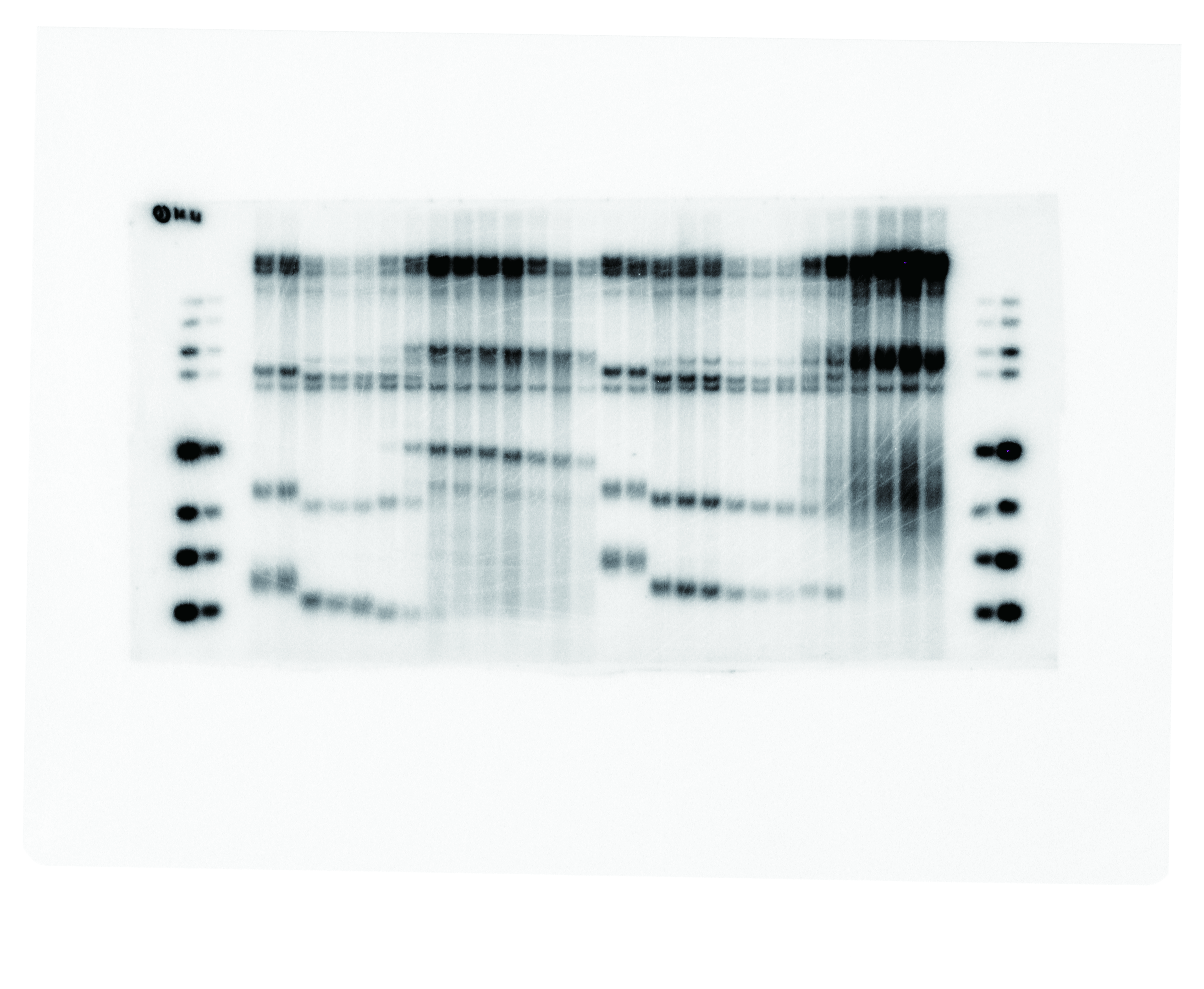

Supplement: Figure 2—source data 2. [file elife-91223-fig2-data2.zip › Figure2C source data1.tif]

C

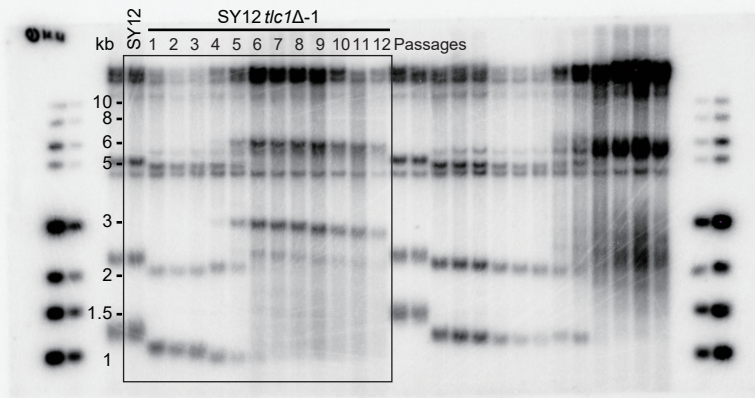

Supplement: Figure 2—source data 3. [file elife-91223-fig2-data3.zip › PDF containing Figure 2C and original scans of the relevant Southern blot analysis.pdf]

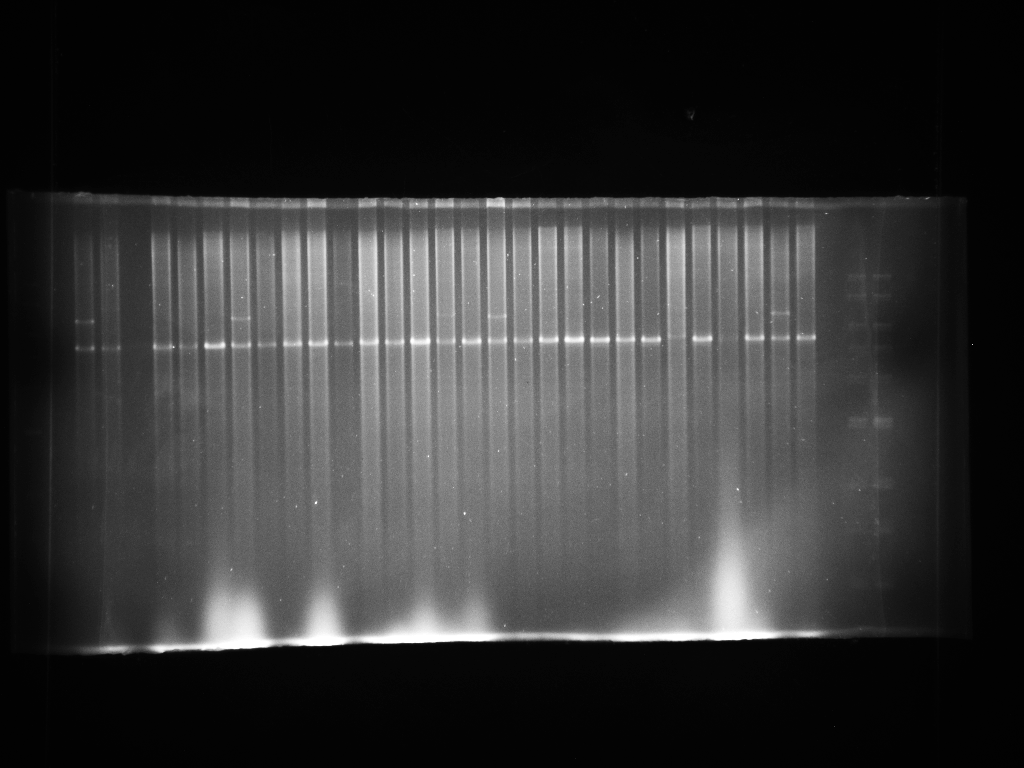

Supplement: Figure 2—source data 6. [file elife-91223-fig2-data6.zip › Figure2D source data3.tif]

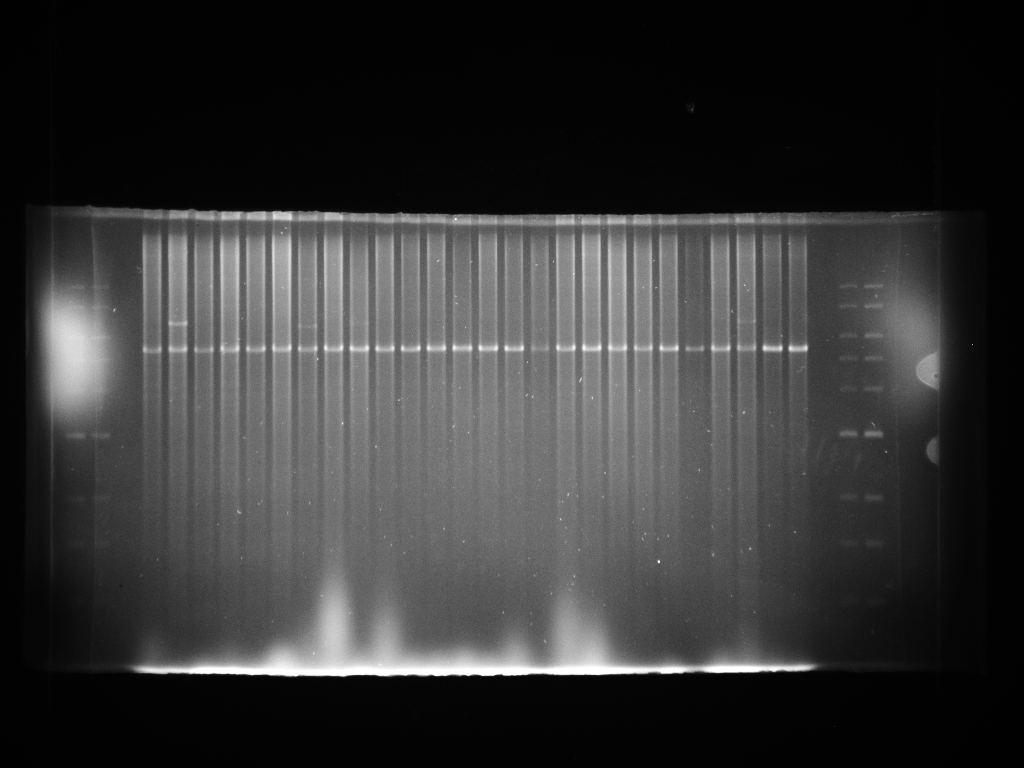

Supplement: Figure 2—source data 7. [file elife-91223-fig2-data7.zip › Figure2D source data4.tif]

D

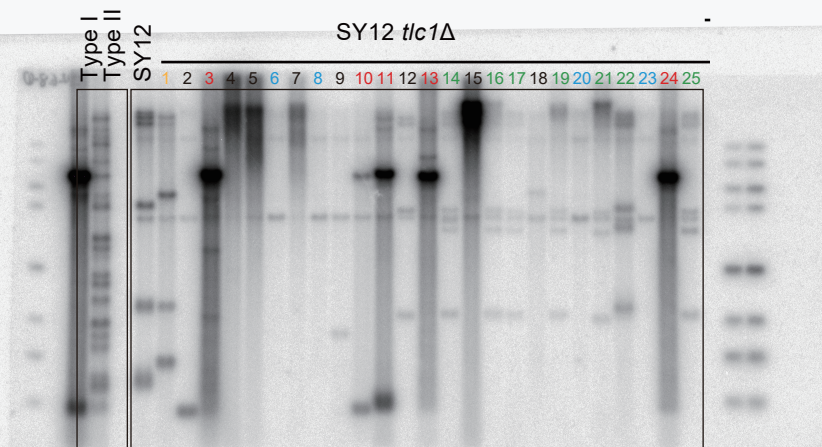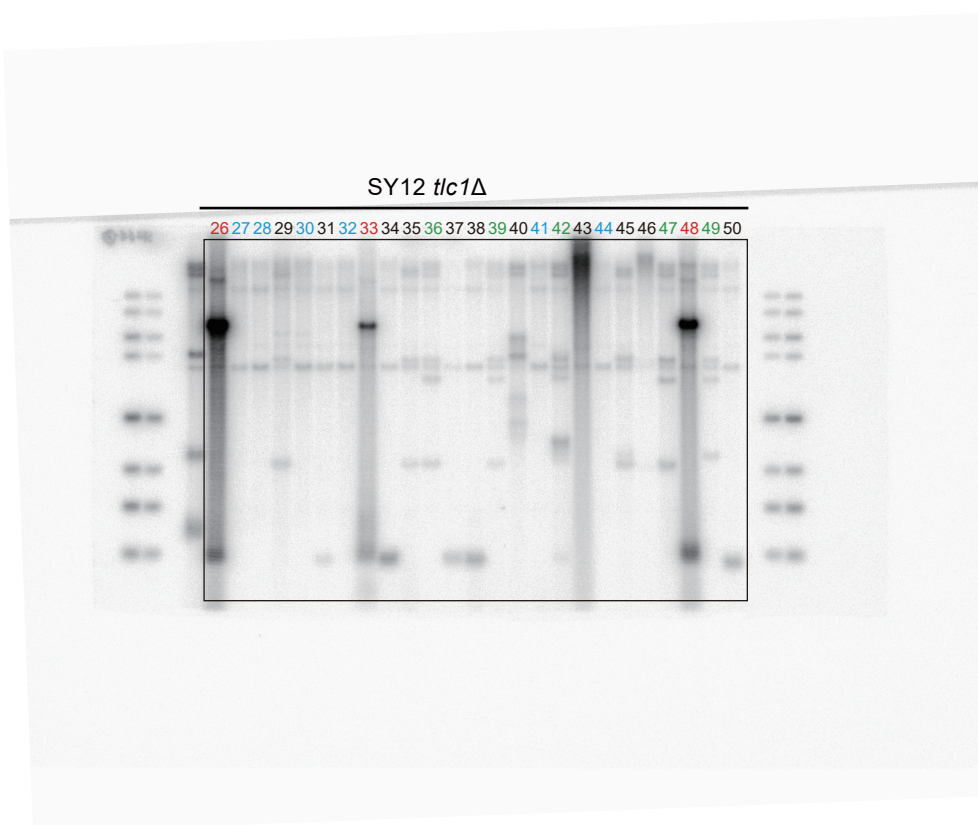

Supplement: Figure 2—source data 8. [file elife-91223-fig2-data8.zip › PDF containing Figure 2D and original scans of the relevant Southern blot analysis.pdf]

D

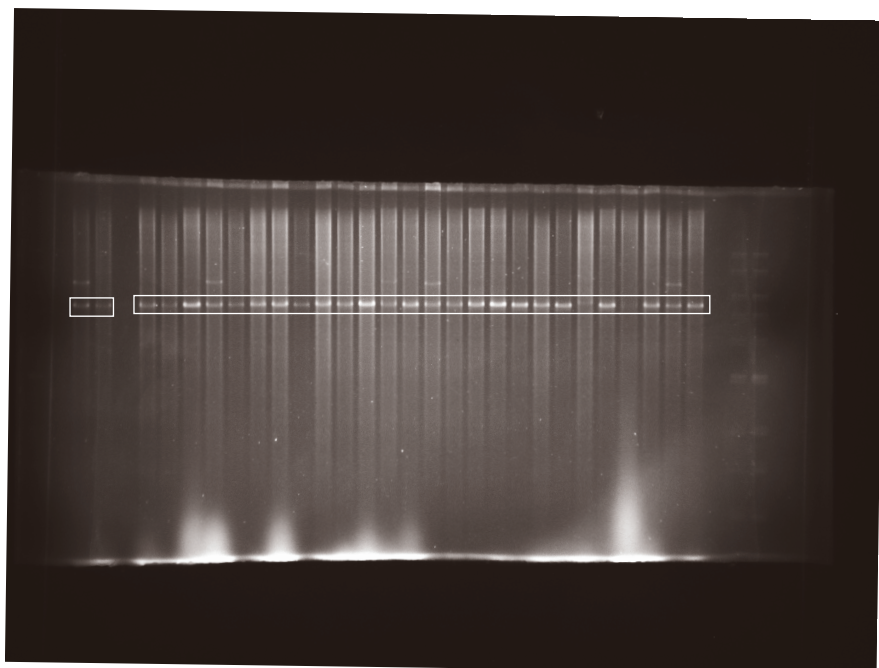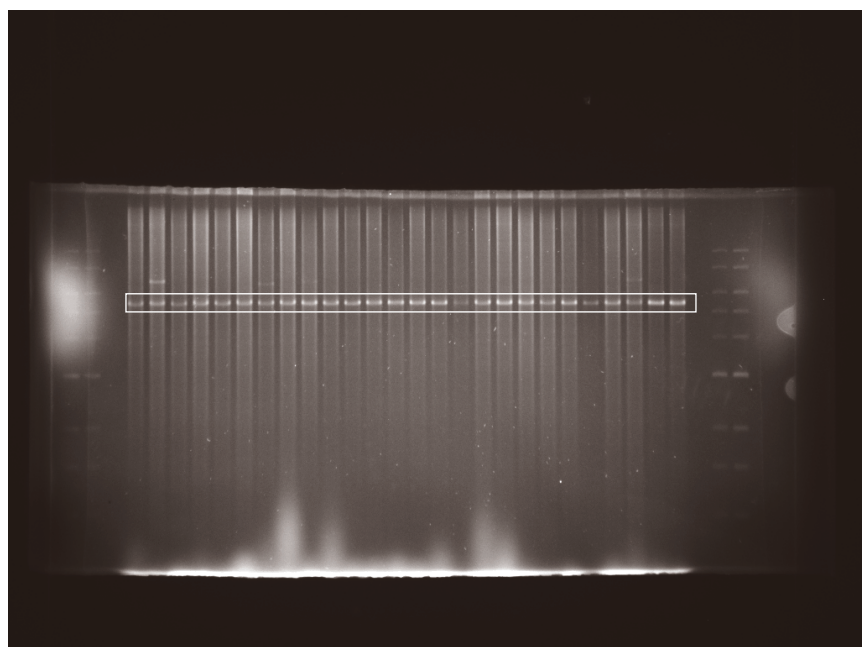

Supplement: Figure 2—source data 9. [file elife-91223-fig2-data9.zip › PDF containing original scans of the loading contral in Figure 2D.pdf]

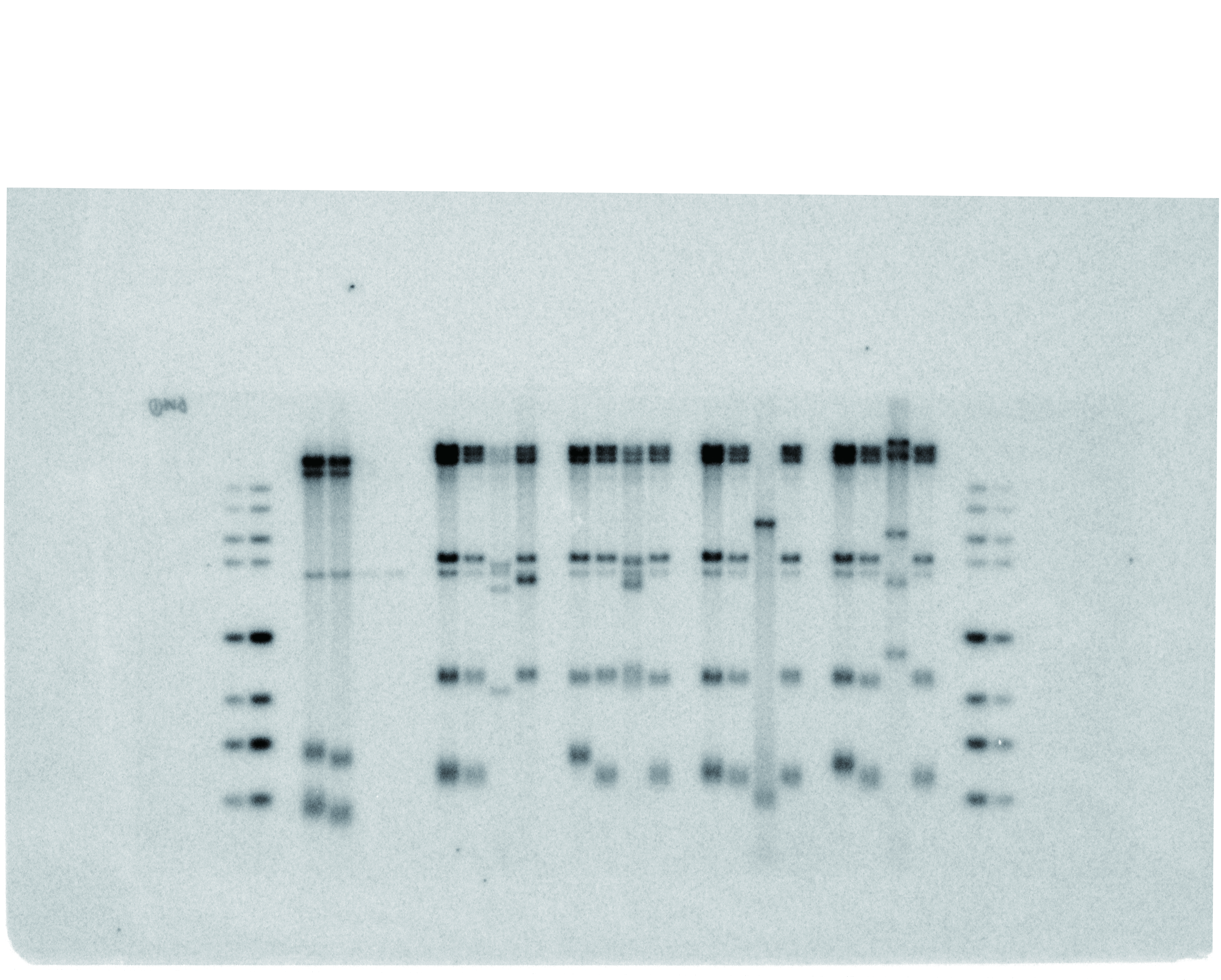

Supplement: Figure 2—source data 10. [file elife-91223-fig2-data10.zip › Figure2H source data1.tif]

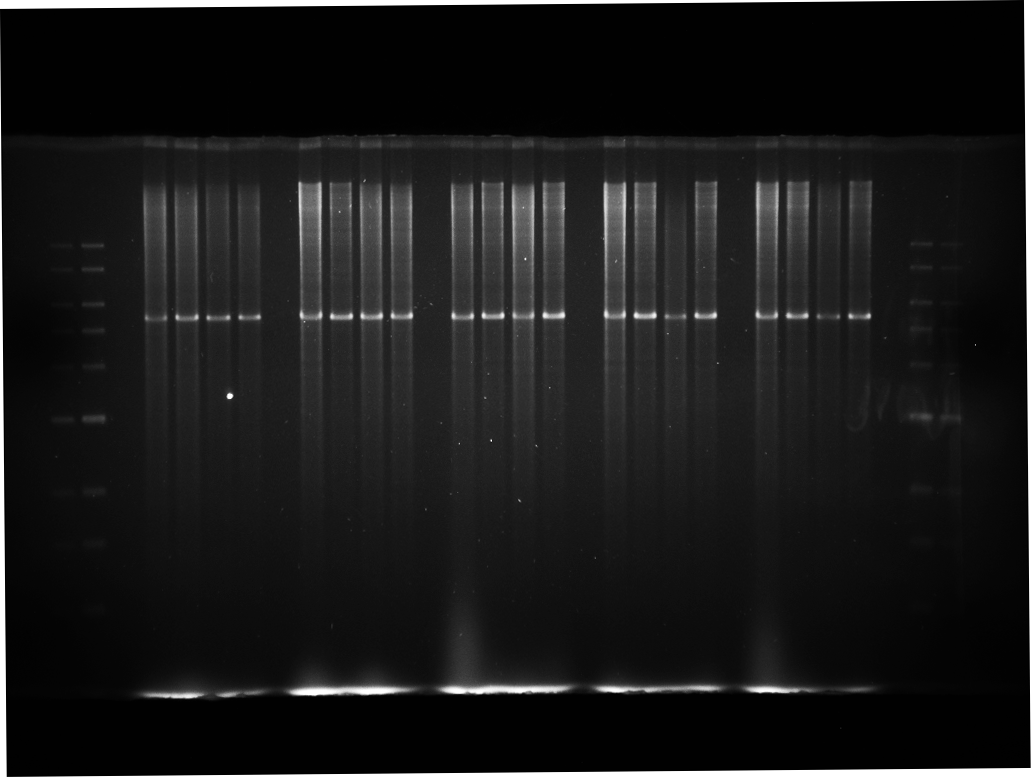

Supplement: Figure 2—source data 11. [file elife-91223-fig2-data11.zip › Figure2G source data2.tif]

G

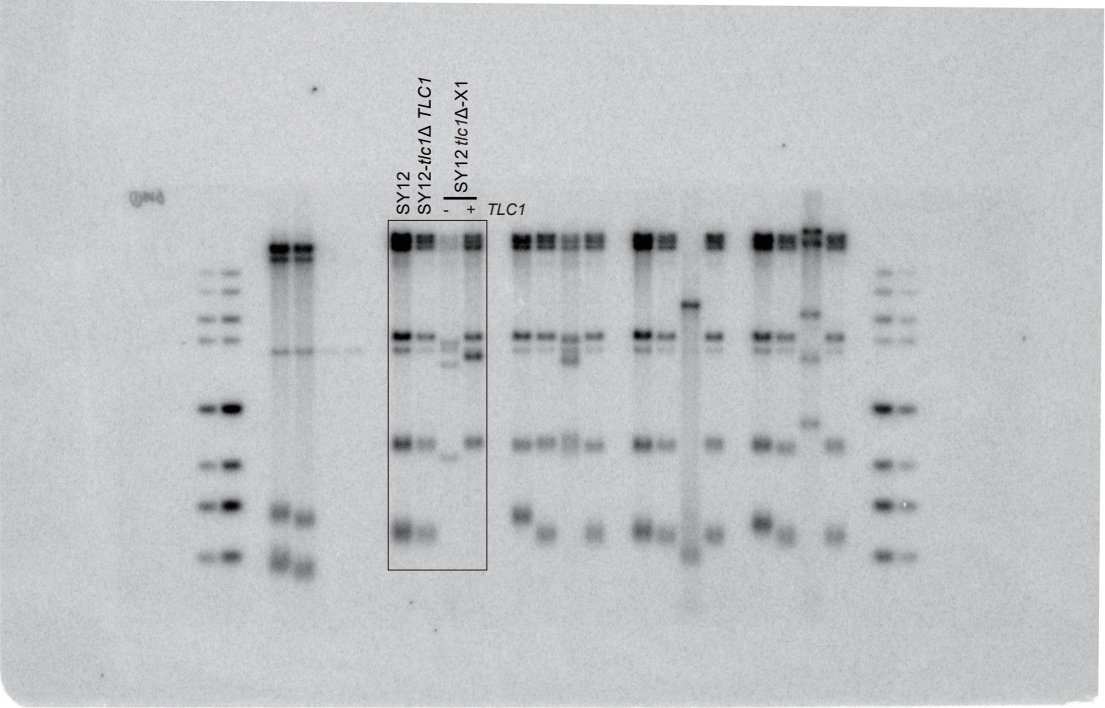

Supplement: Figure 2—source data 12. [file elife-91223-fig2-data12.zip › PDF containing Figure 2G and original scans of the relevant Southern blot analysis.pdf]

SY12  
SY12-*tlc1Δ* TLC1  
+ SY12 *tlc1Δ*-X1

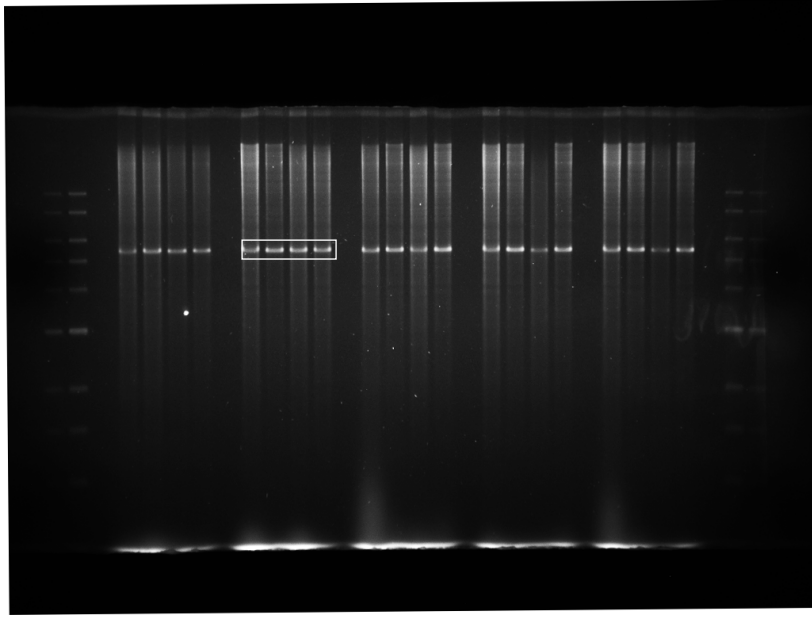

Supplement: Figure 2—source data 13. [file elife-91223-fig2-data13.zip › PDF containing original scans of the loading contral in Figure 2G.pdf]

**B**

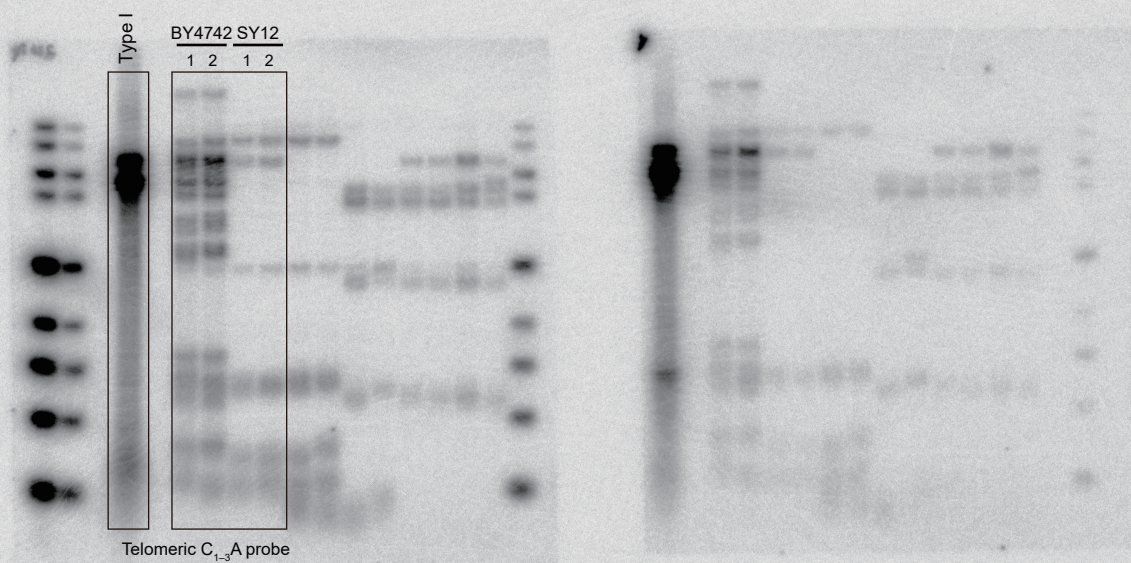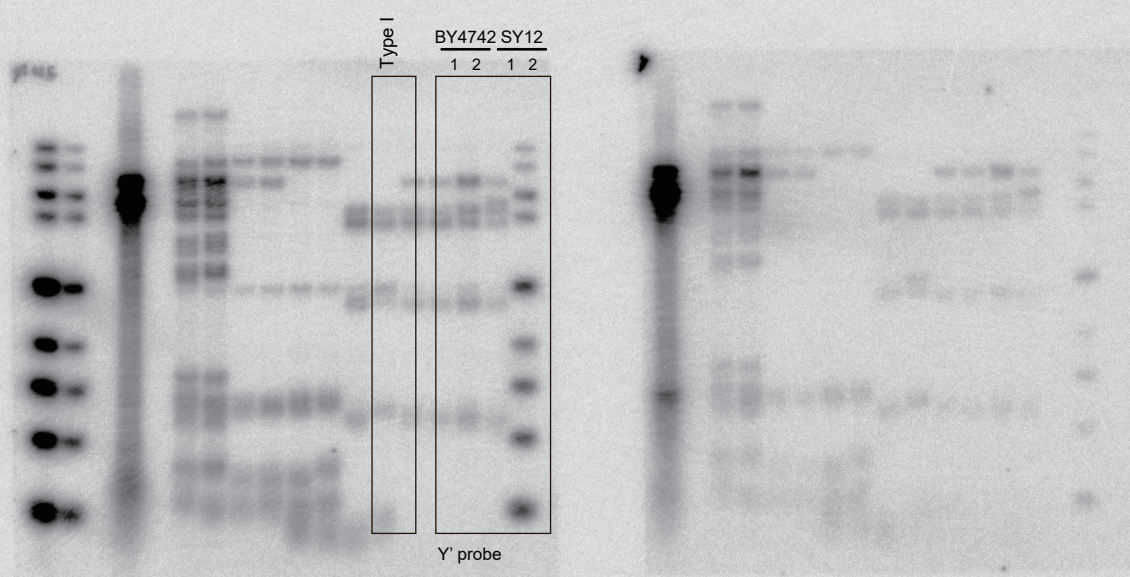

Supplement: Figure 2—figure supplement 1—source data 3. [file elife-91223-fig2-figsupp1-data3.zip › PDF containing Figure 2-figure supplementary1 and original scans of the relevant Southern blot analysis.pdf]

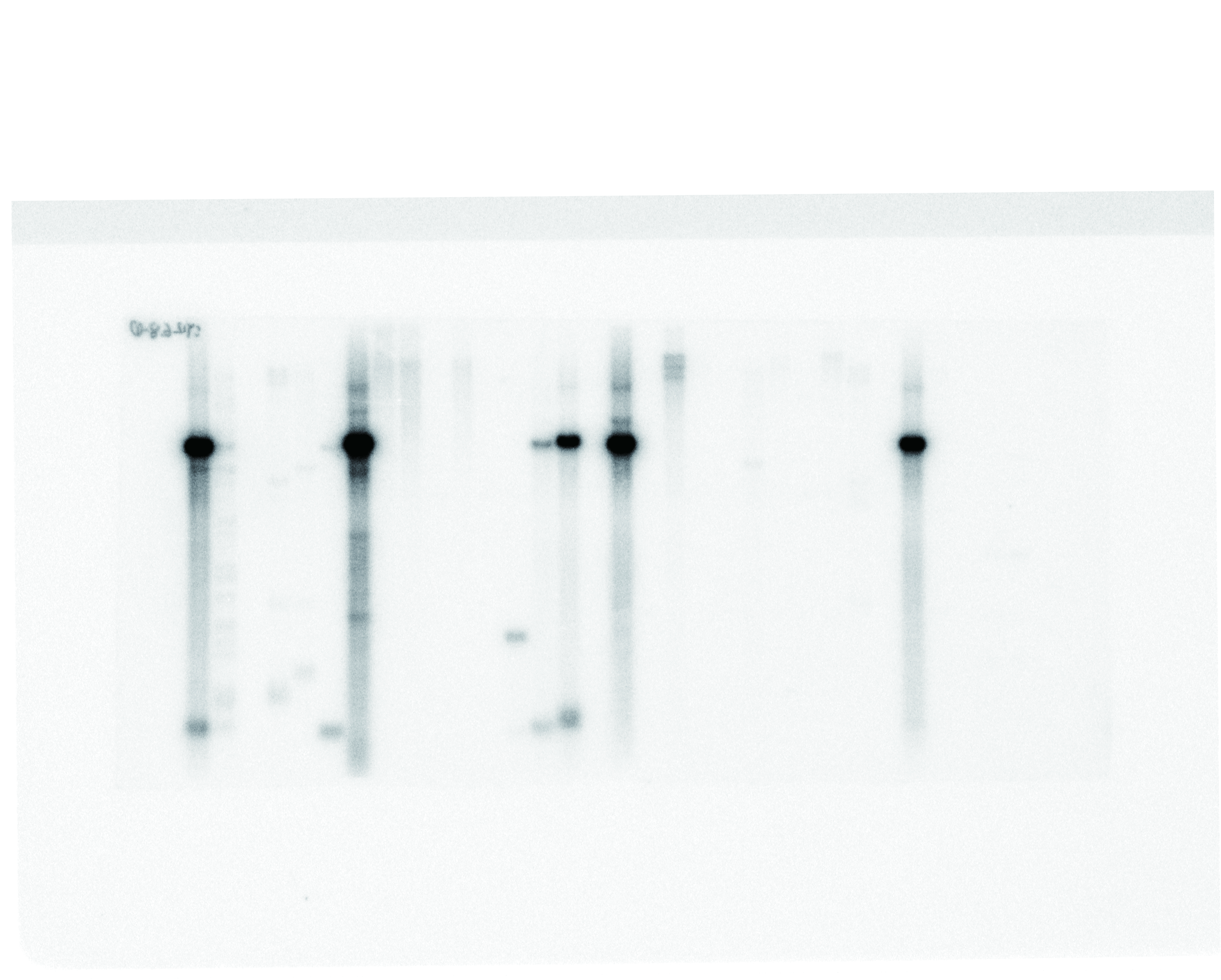

Supplement: Figure 2—figure supplement 2—source data 1. [file elife-91223-fig2-figsupp2-data1.zip › Figure2-figure supplementary2- source data1.tif]

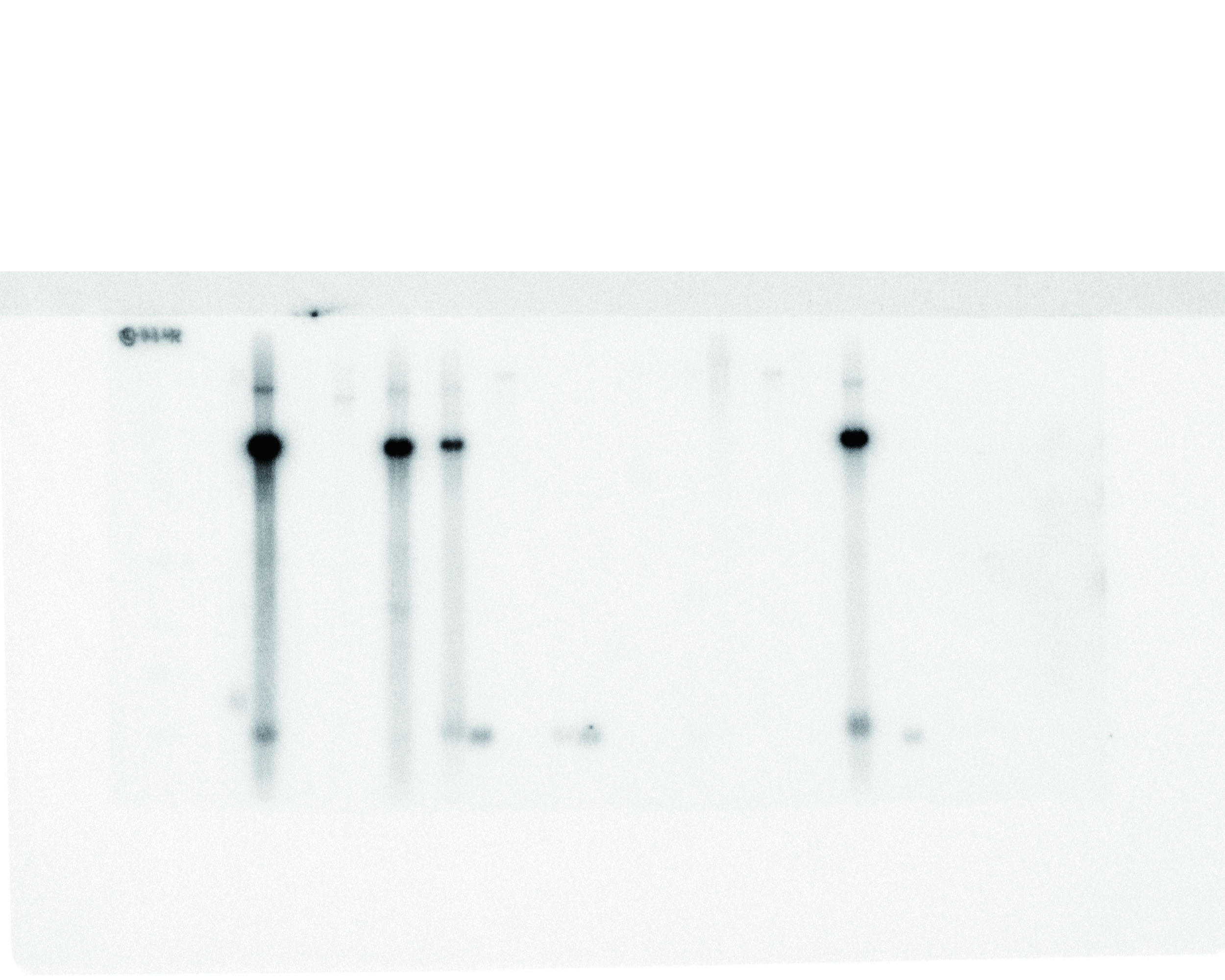

Supplement: Figure 2—figure supplement 2—source data 2. [file elife-91223-fig2-figsupp2-data2.zip › Figure2-figure supplementary2- source data2.tif]

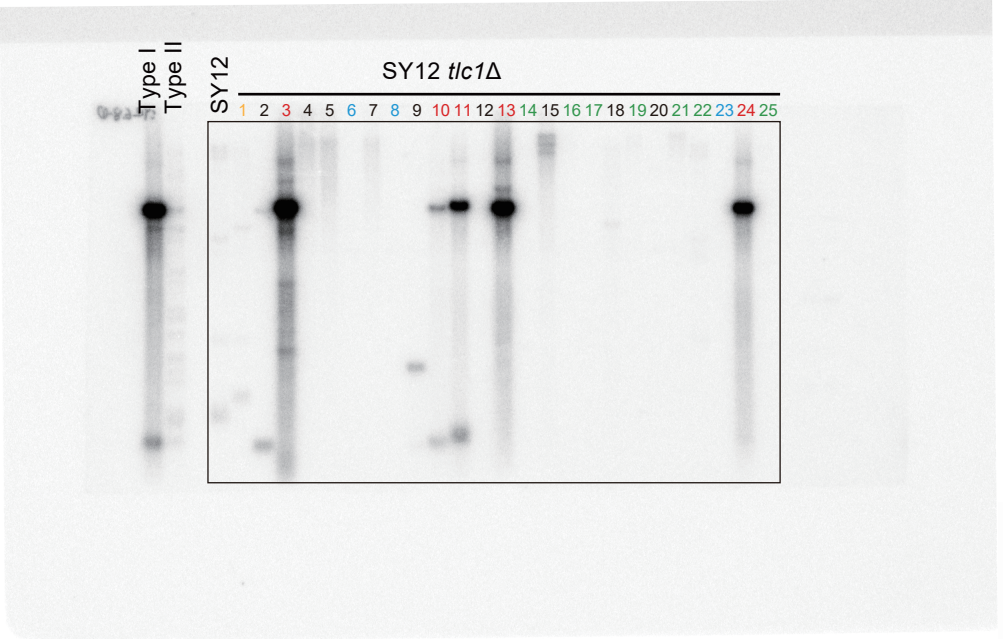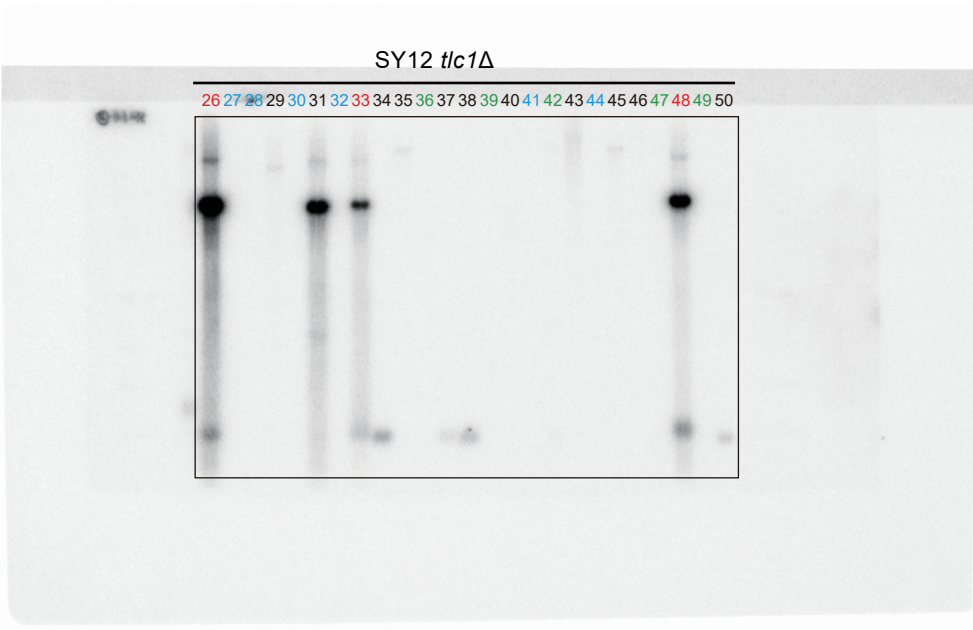

Supplement: Figure 2—figure supplement 2—source data 3. [file elife-91223-fig2-figsupp2-data3.zip › PDF containing Figure 2-figure supplementary2 and original scans of the relevant Southern blot analysis.pdf]

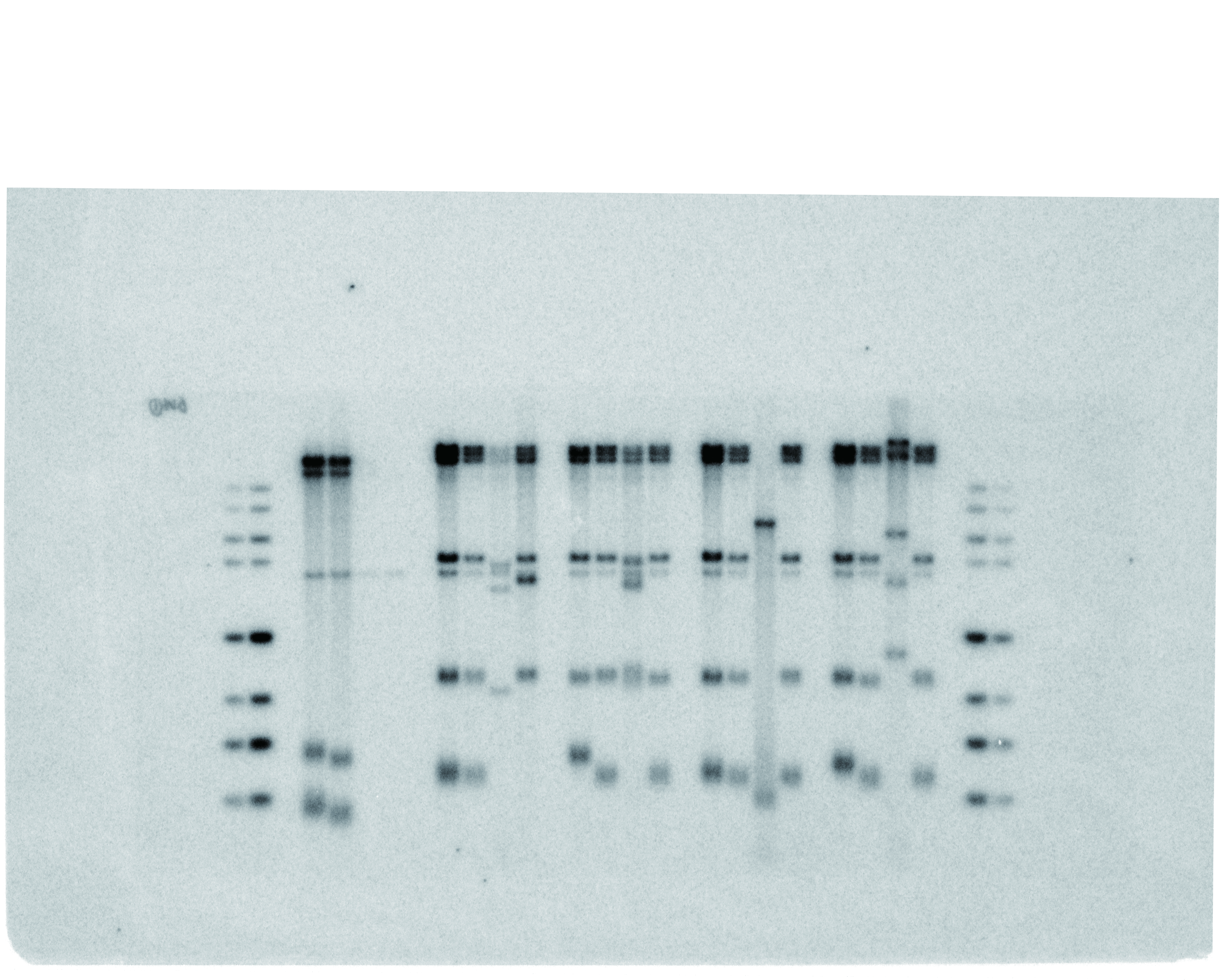

Supplement: Figure 2—figure supplement 3—source data 1. [file elife-91223-fig2-figsupp3-data1.zip › Figure2-figure supplementary3- source data1.tif]

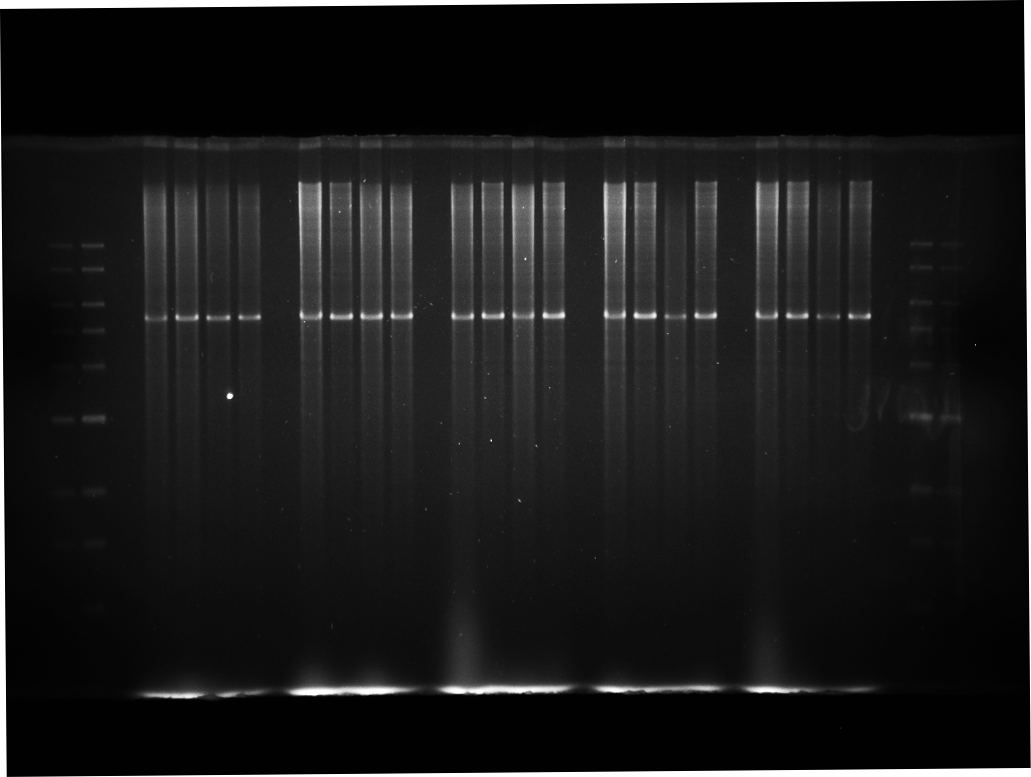

Supplement: Figure 2—figure supplement 3—source data 3. [file elife-91223-fig2-figsupp3-data3.zip › Figure2-figure supplementary3- source data 3.tif]

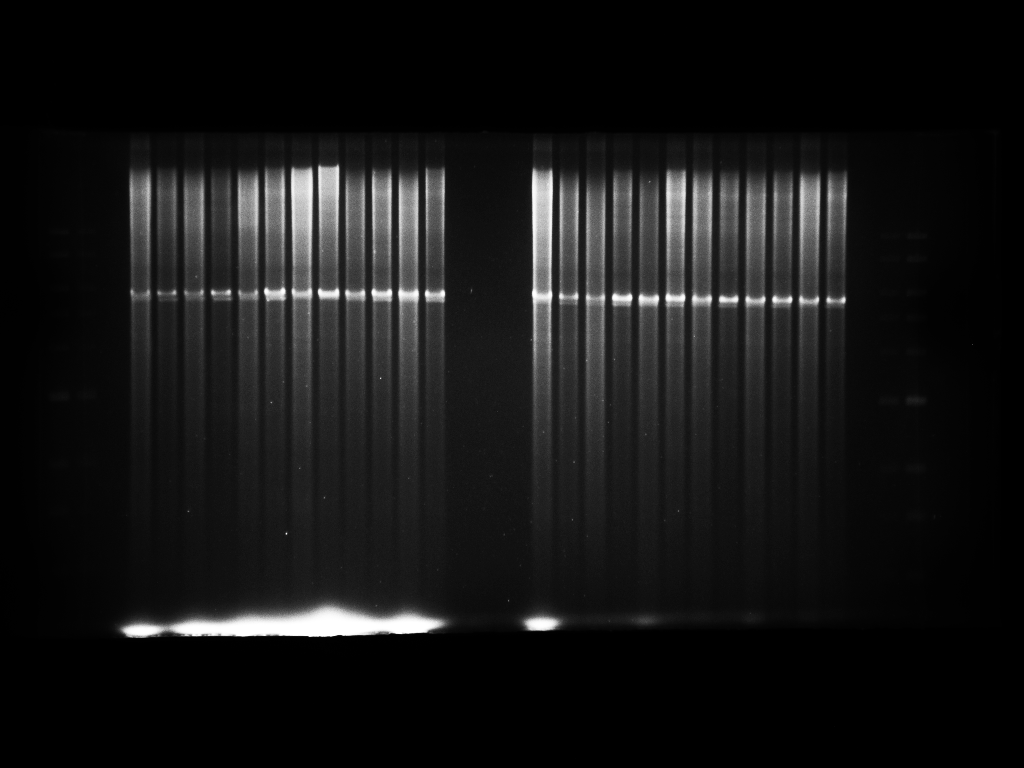

Supplement: Figure 2—figure supplement 3—source data 4. [file elife-91223-fig2-figsupp3-data4.zip › Figure2-figure supplementary3- source data 4.tif]

**A**

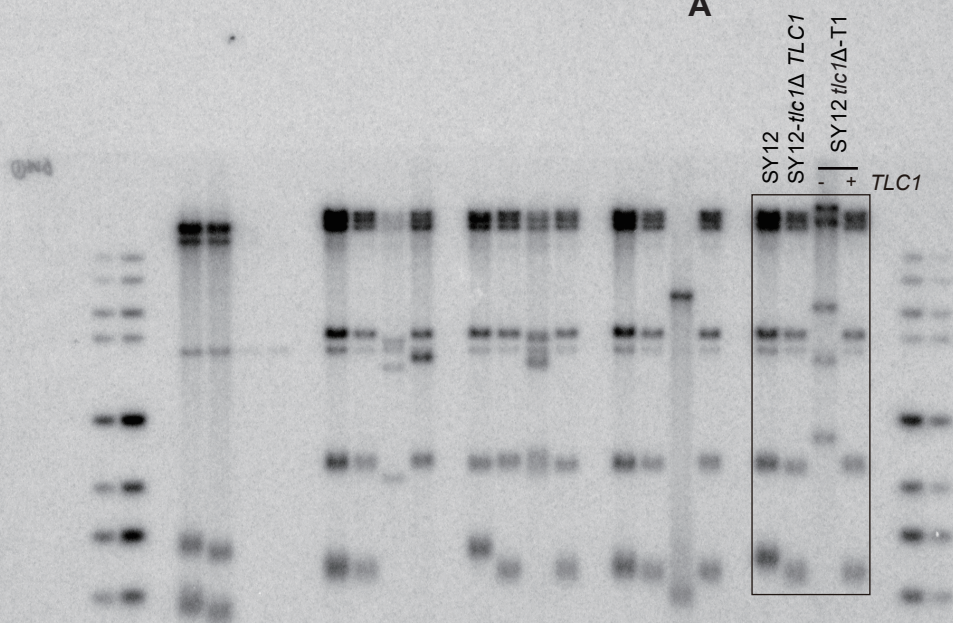

**B**

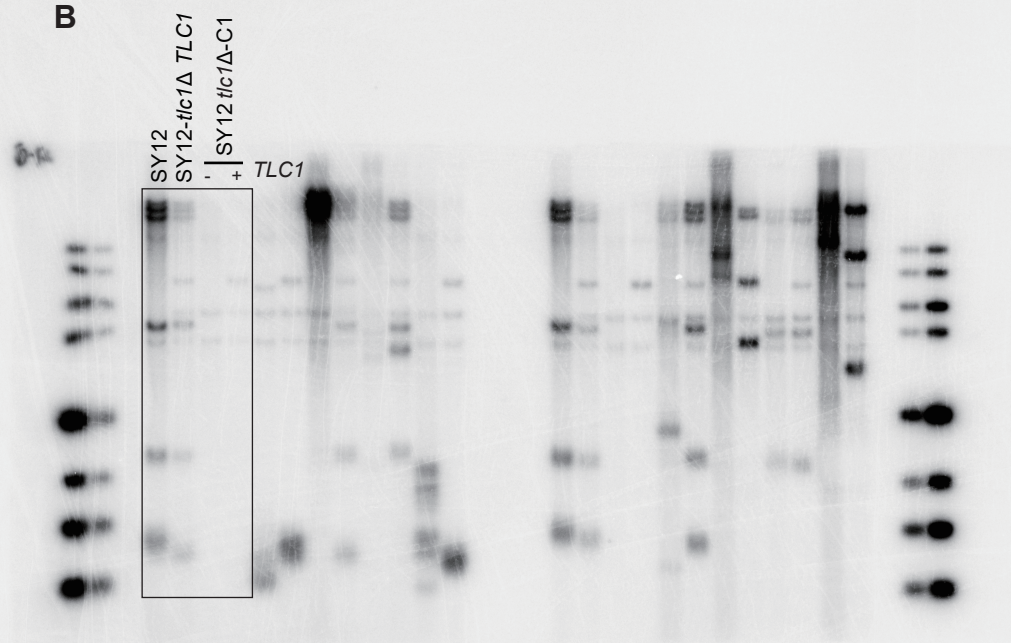

Supplement: Figure 2—figure supplement 3—source data 5. [file elife-91223-fig2-figsupp3-data5.zip › PDF containing Figure 2-figure supplementary3 and original scans of the relevant Southern blot analysis.pdf]

**E**

SY12  
SY12-*tlc1Δ* TLC1  
- +  
SY12-*tlc1Δ*-T1

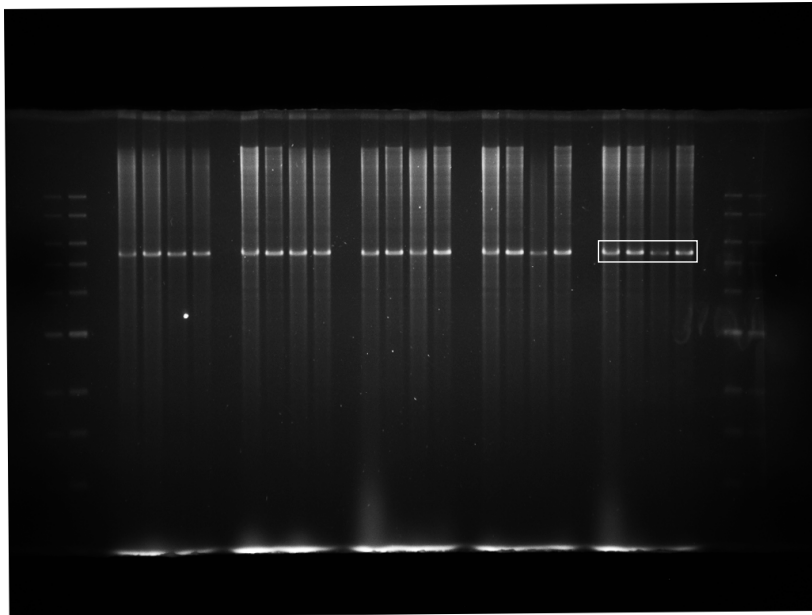

**G**

SY12  
SY12-*tlc1Δ* TLC1  
- +  
SY12-*tlc1Δ*-C1

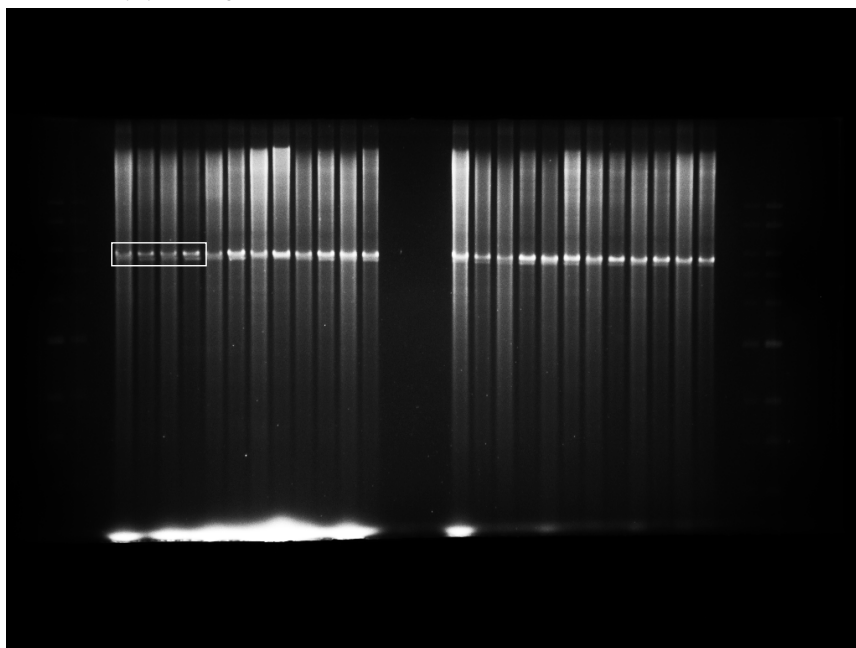

Supplement: Figure 2—figure supplement 3—source data 6. [file elife-91223-fig2-figsupp3-data6.zip › PDF containing original scans of the loading contral in Figure 2-figure supplementary 3.pdf]

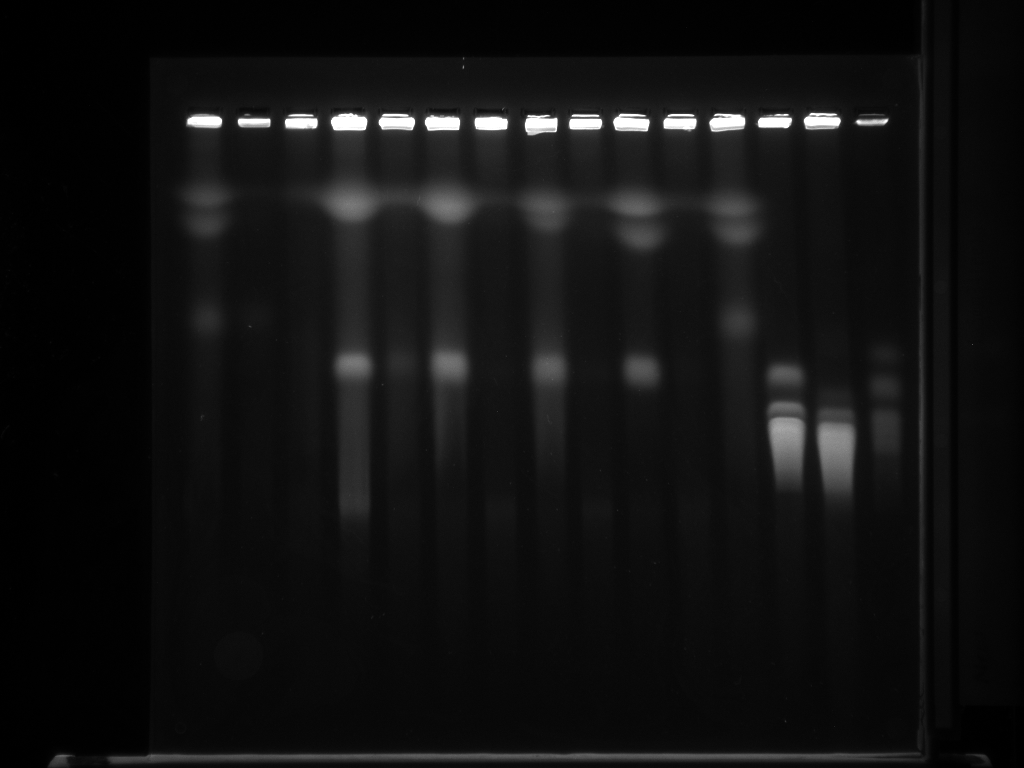

Supplement: Figure 2—figure supplement 5—source data 1. [file elife-91223-fig2-figsupp5-data1.zip › Figure2-figure supplementary5- source data2.Tif]

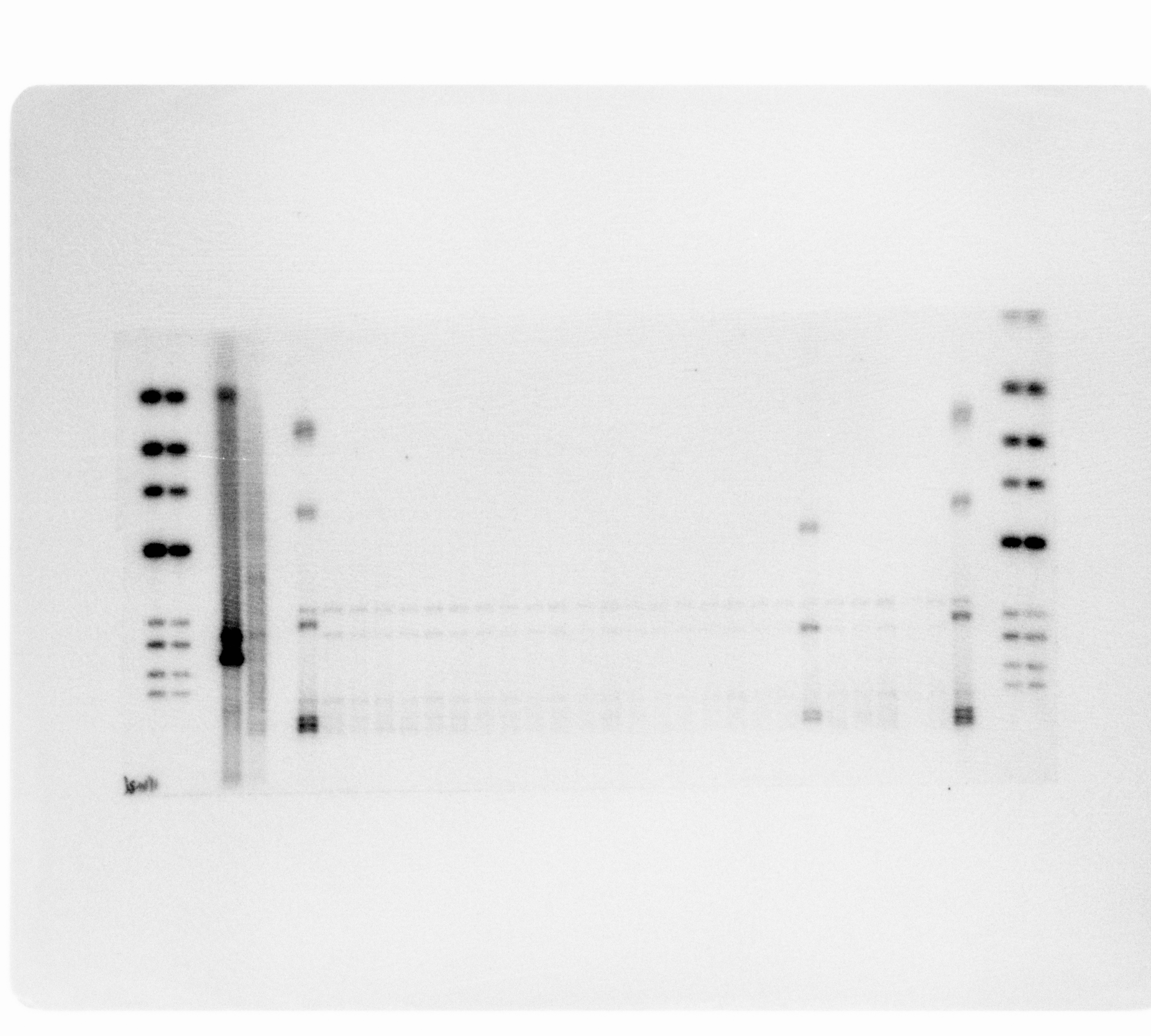

Supplement: Figure 2—figure supplement 7—source data 2. [file elife-91223-fig2-figsupp7-data2.zip › Figure2-figure supplementary7- source data1.tif]

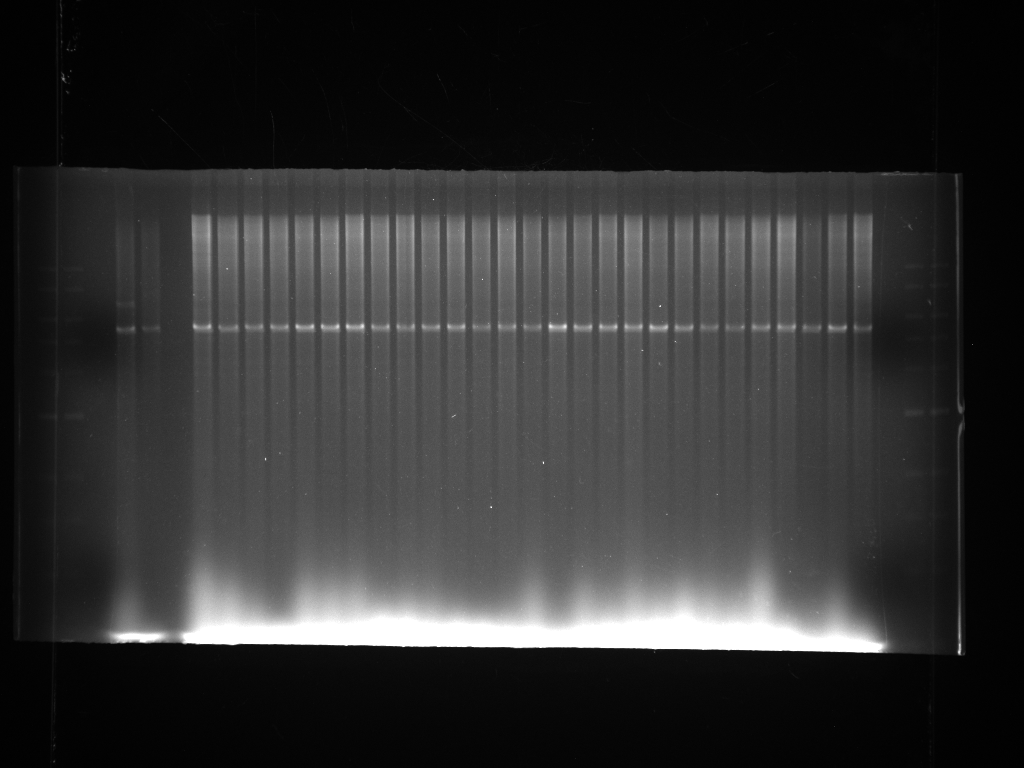

Supplement: Figure 2—figure supplement 7—source data 3. [file elife-91223-fig2-figsupp7-data3.zip › Figure2-figure supplementary7- source data2.Tif]

2013

SY12

SY12 *tlc1Δ rad52Δ*

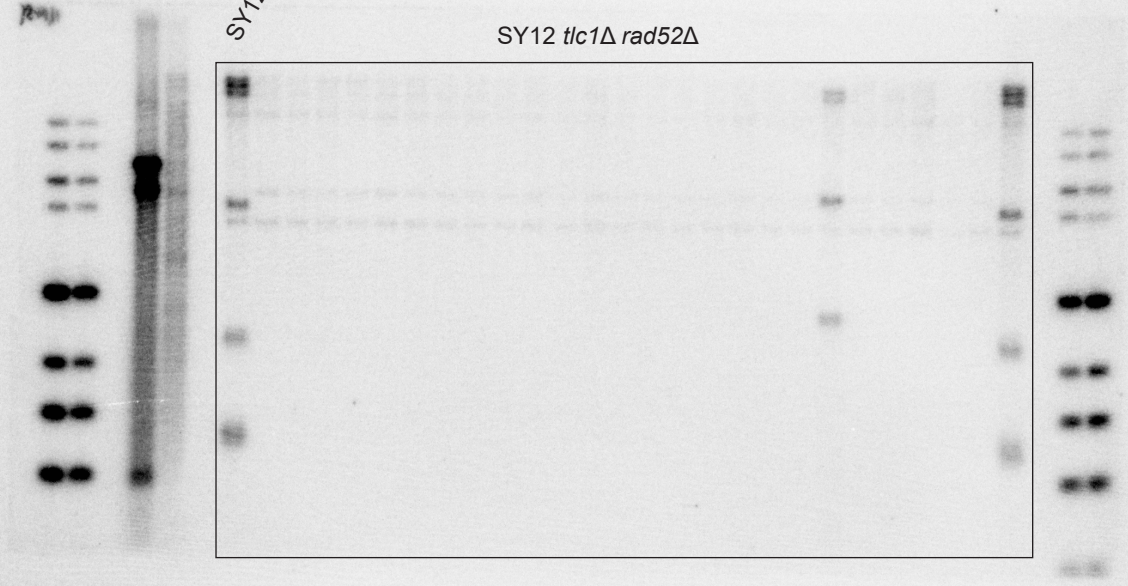

Supplement: Figure 2—figure supplement 7—source data 4. [file elife-91223-fig2-figsupp7-data4.zip › PDF containing Figure 2-figure supplementary7 and original scans of the relevant Southern blot analysis.pdf]

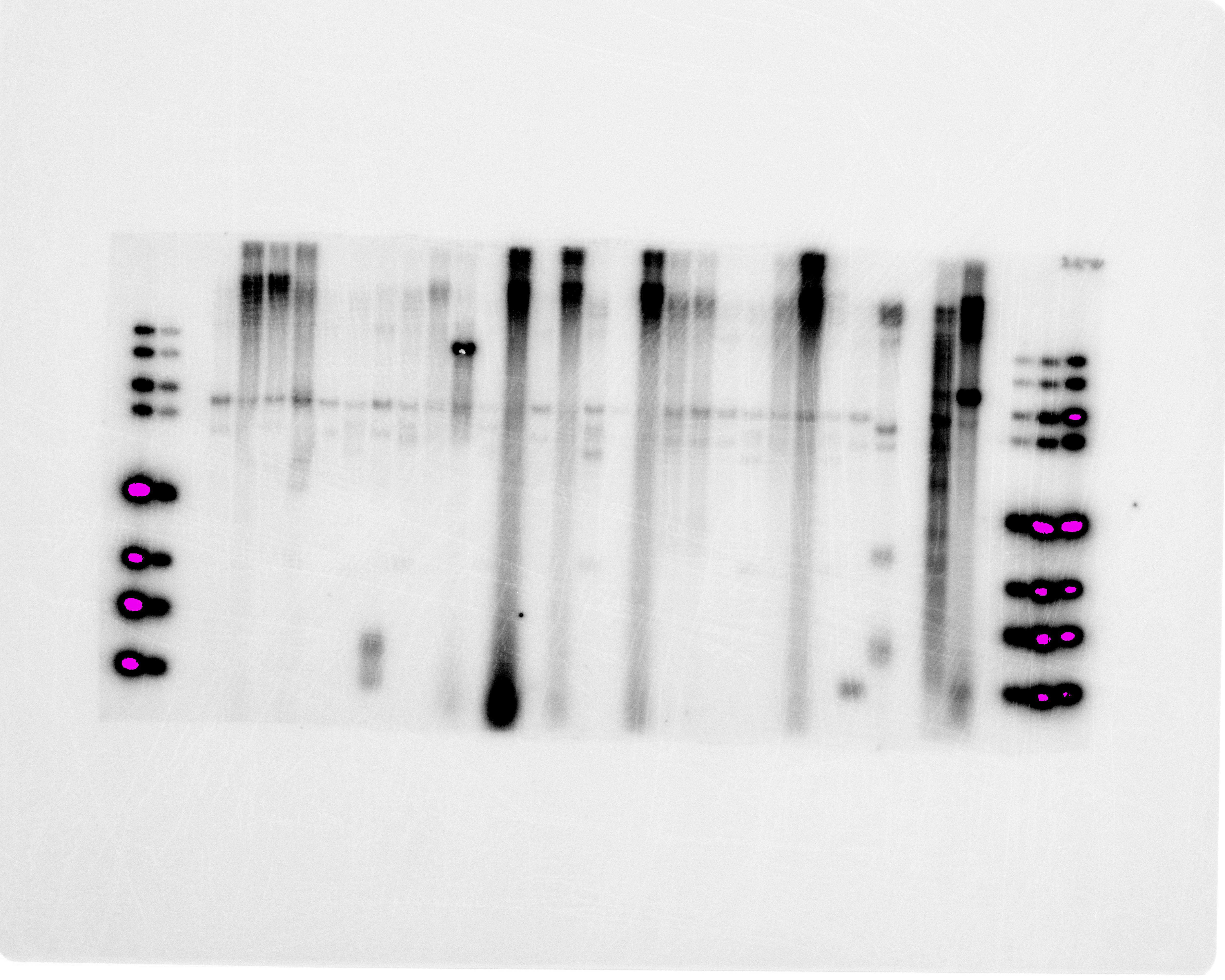

Supplement: Figure 2—figure supplement 8—source data 1. [file elife-91223-fig2-figsupp8-data1.zip › Figure2-figure supplementary8- source data1.tif]

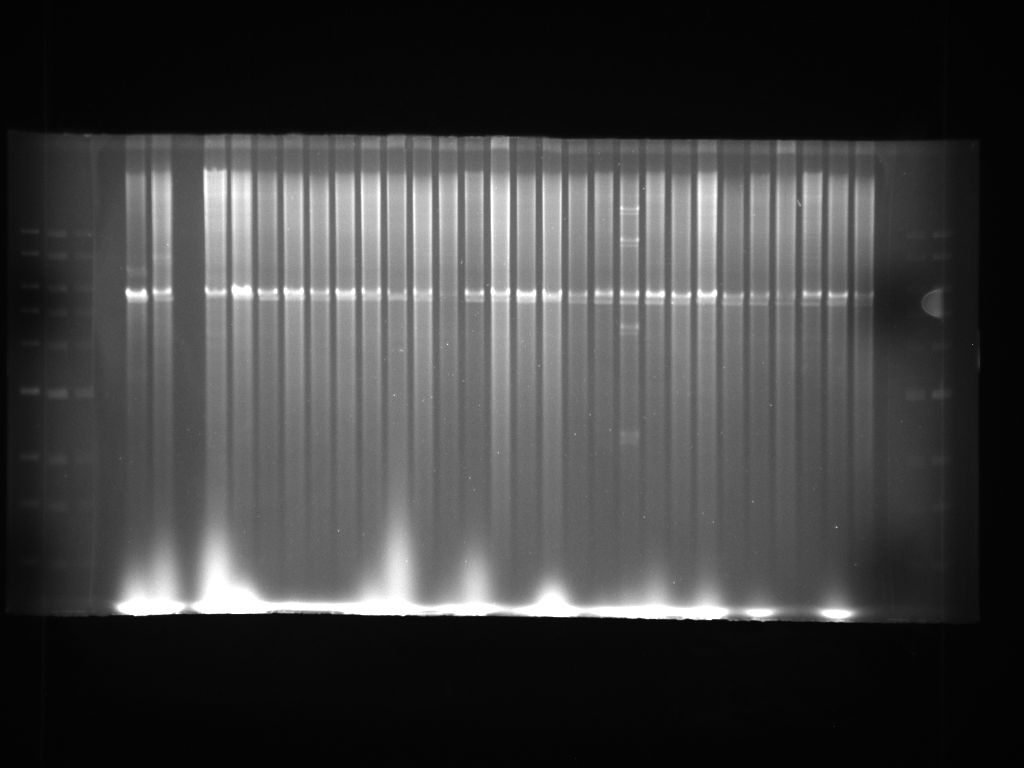

Supplement: Figure 2—figure supplement 8—source data 2. [file elife-91223-fig2-figsupp8-data2.zip › Figure2-figure supplementary8- source data3.Tif]

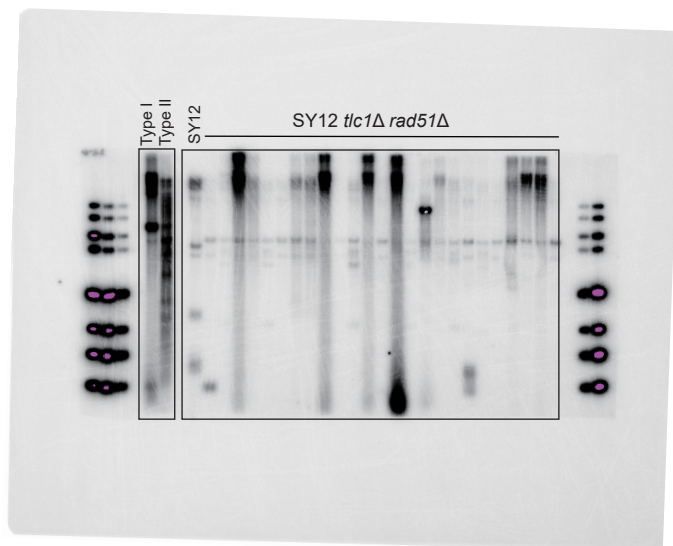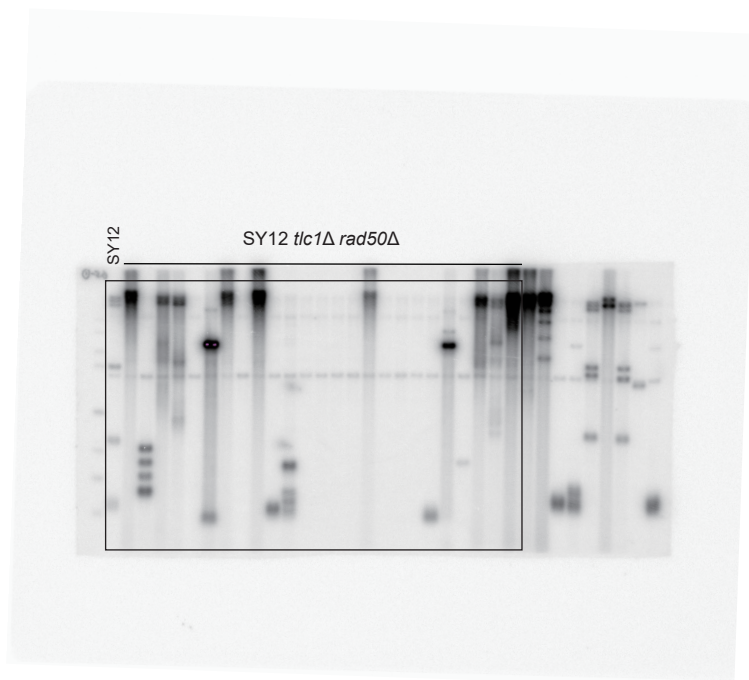

Supplement: Figure 2—figure supplement 8—source data 5. [file elife-91223-fig2-figsupp8-data5.zip › PDF containing Figure 2-figure supplementary8 and original scans of the relevant Southern blot analysis.pdf]

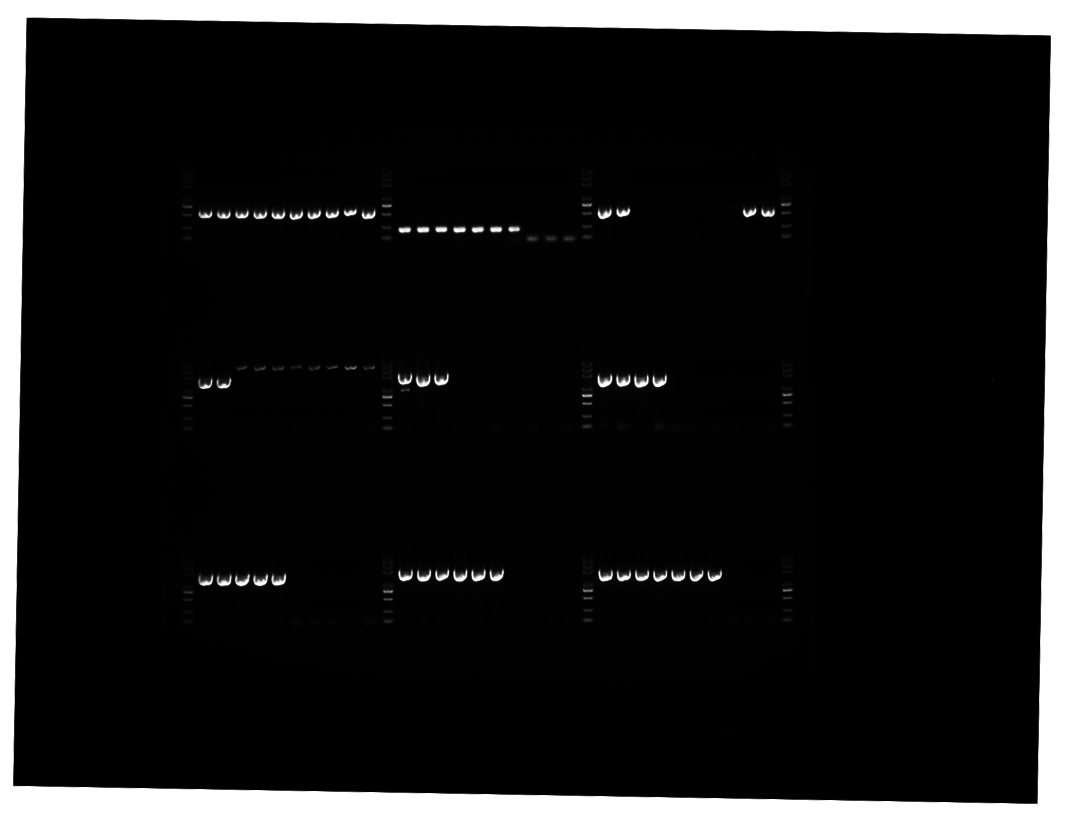

Supplement: Figure 3—source data 1. [file elife-91223-fig3-data1.zip › Figure 3B source data 1.tif]

B

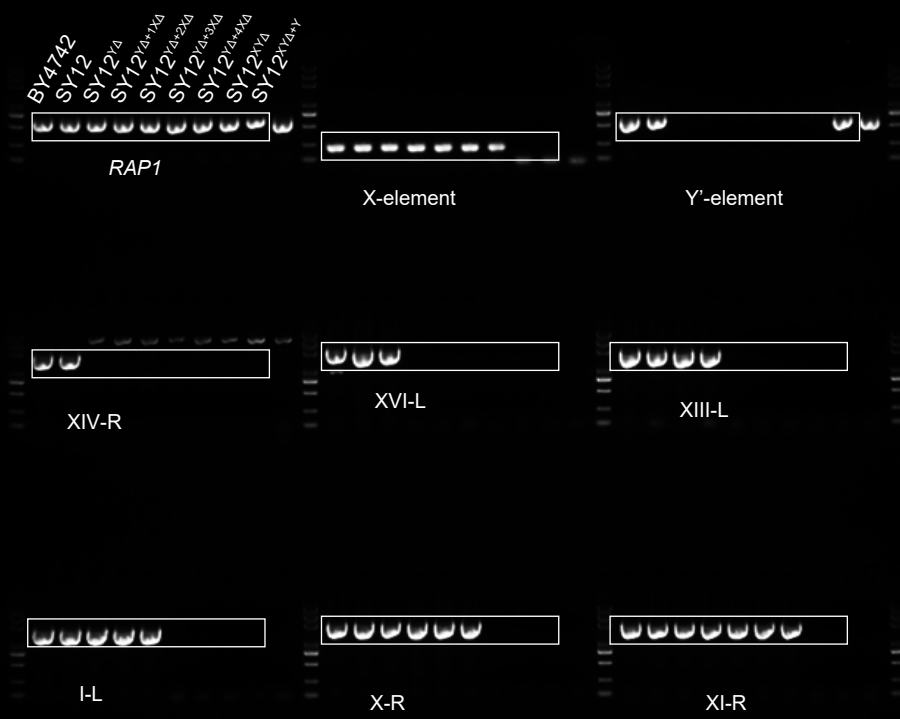

Supplement: Figure 3—source data 2. [file elife-91223-fig3-data2.zip › PDF containing Figure 3B and original scans of the relevant Southern blot analysis.pdf]

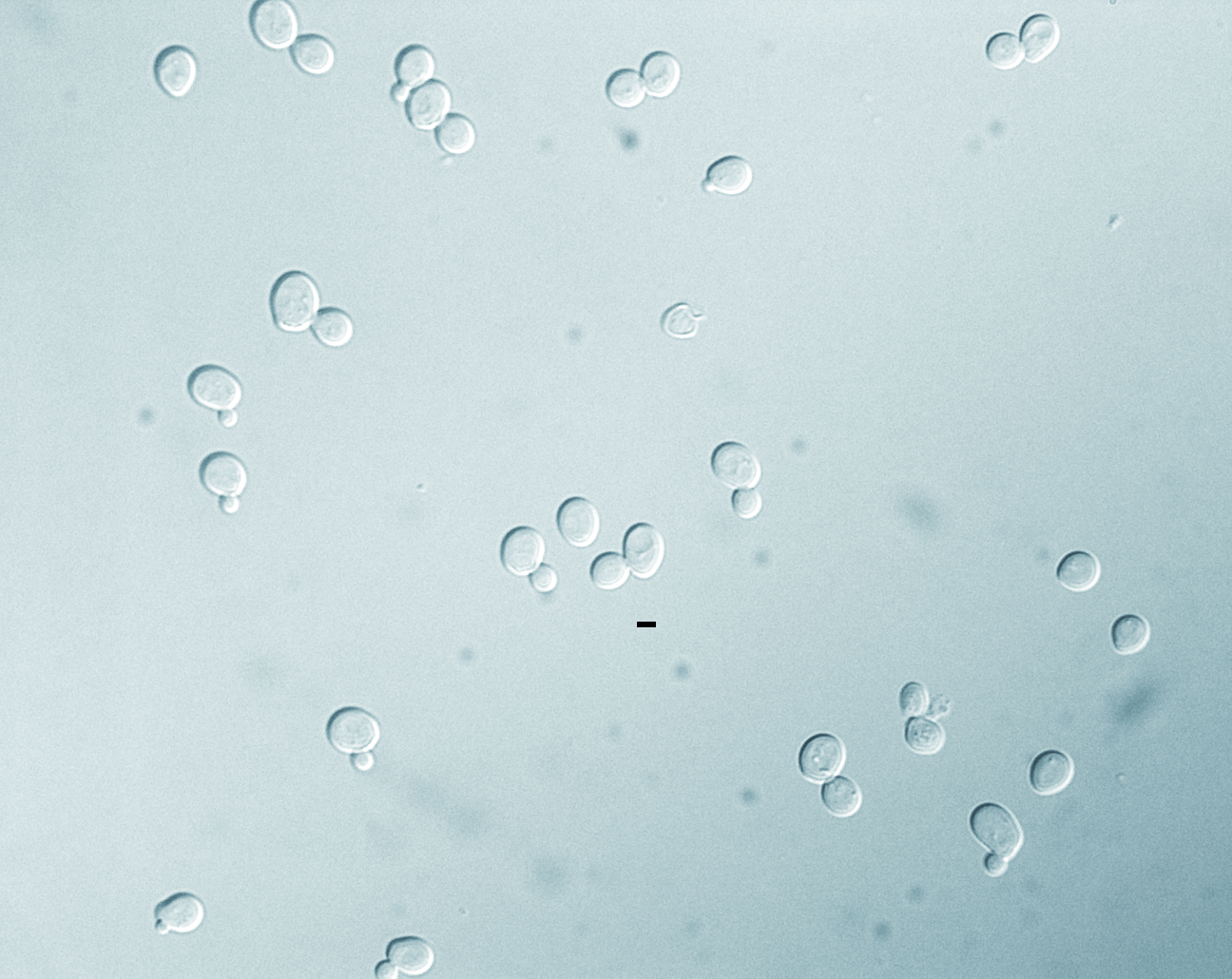

Supplement: Figure 3—source data 3. [file elife-91223-fig3-data3.zip › Figure 3C source data 1.tif]

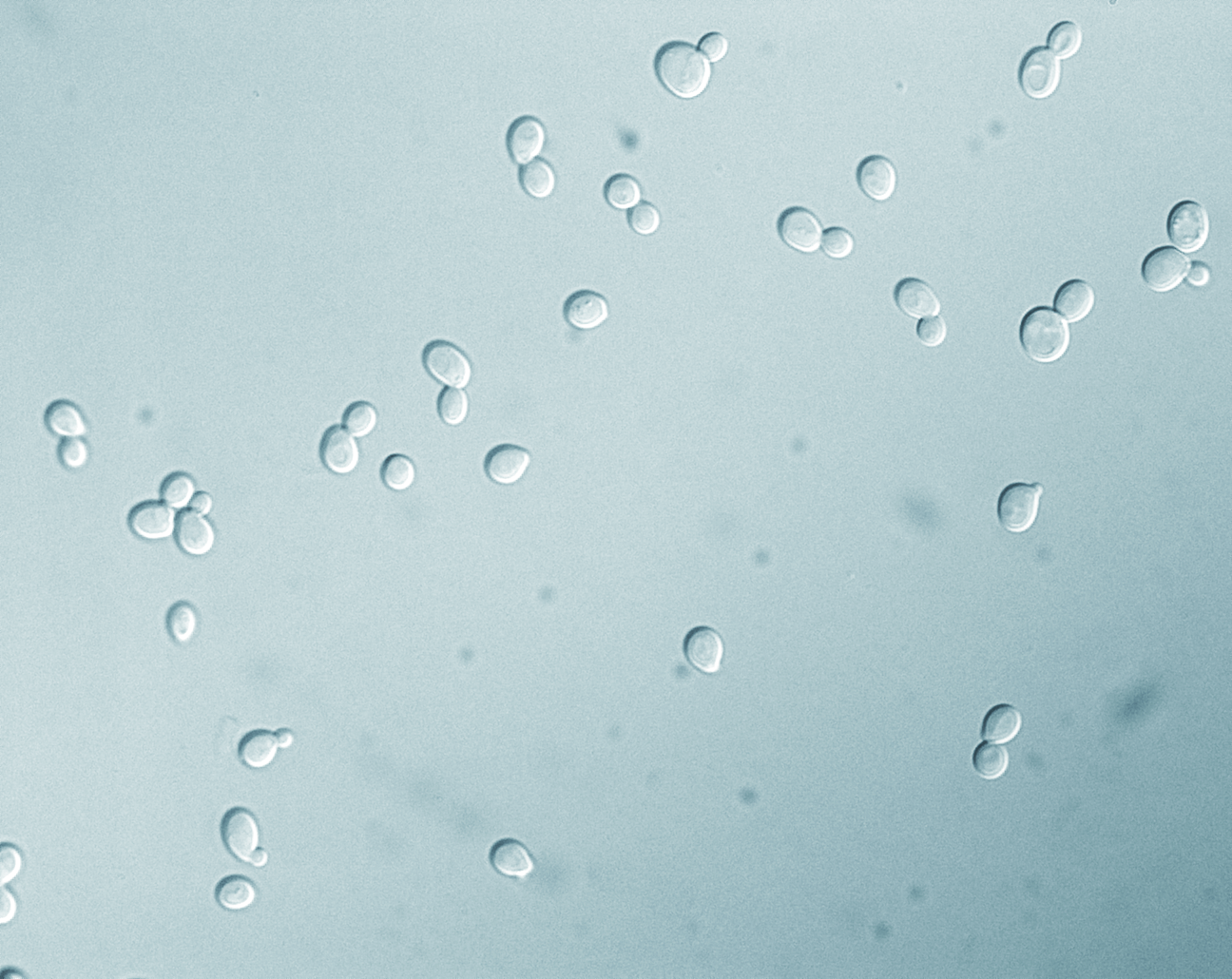

Supplement: Figure 3—source data 4. [file elife-91223-fig3-data4.zip › Figure 3C source data 2.tif]

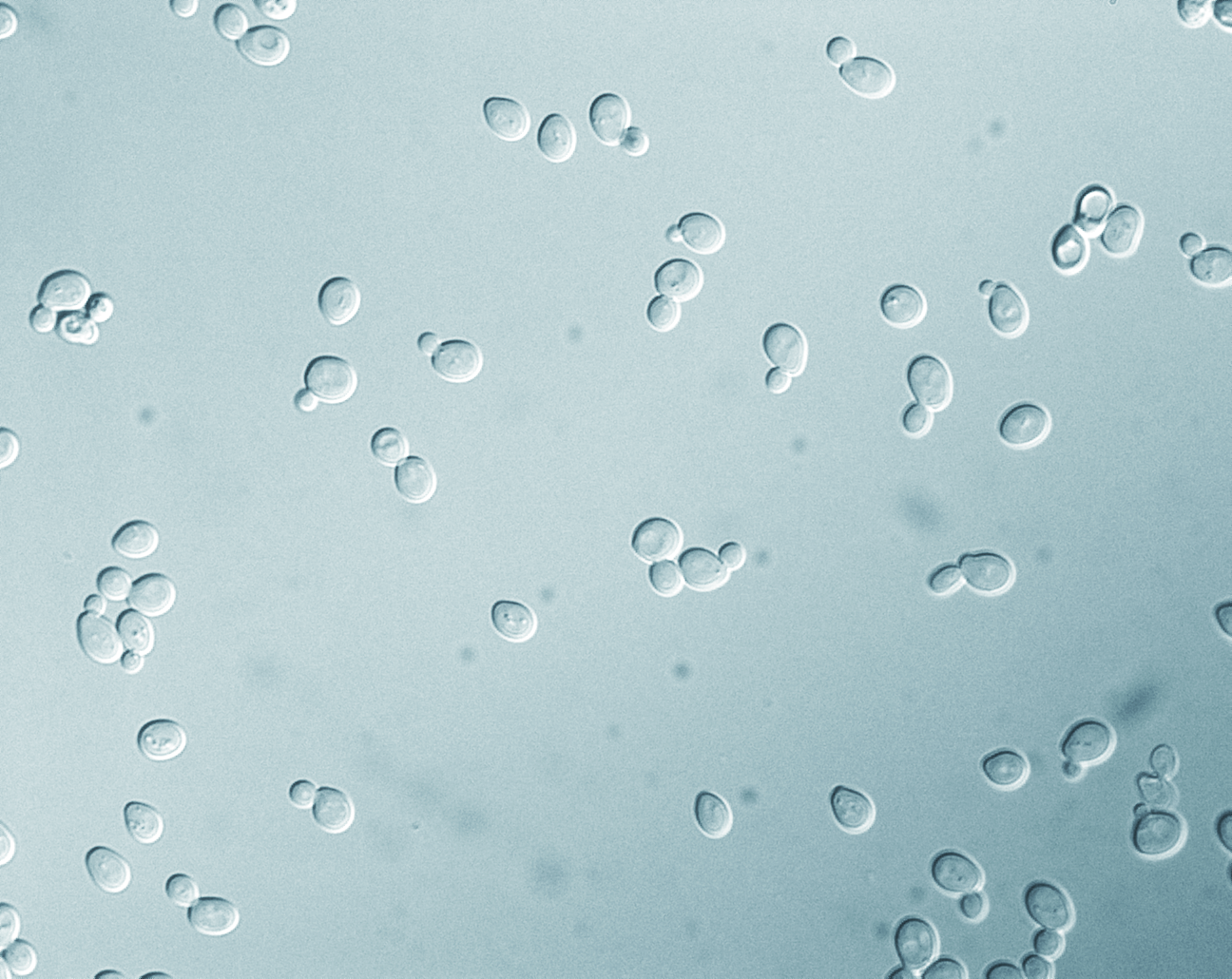

Supplement: Figure 3—source data 5. [file elife-91223-fig3-data5.zip › Figure 3C source data 3.tif]

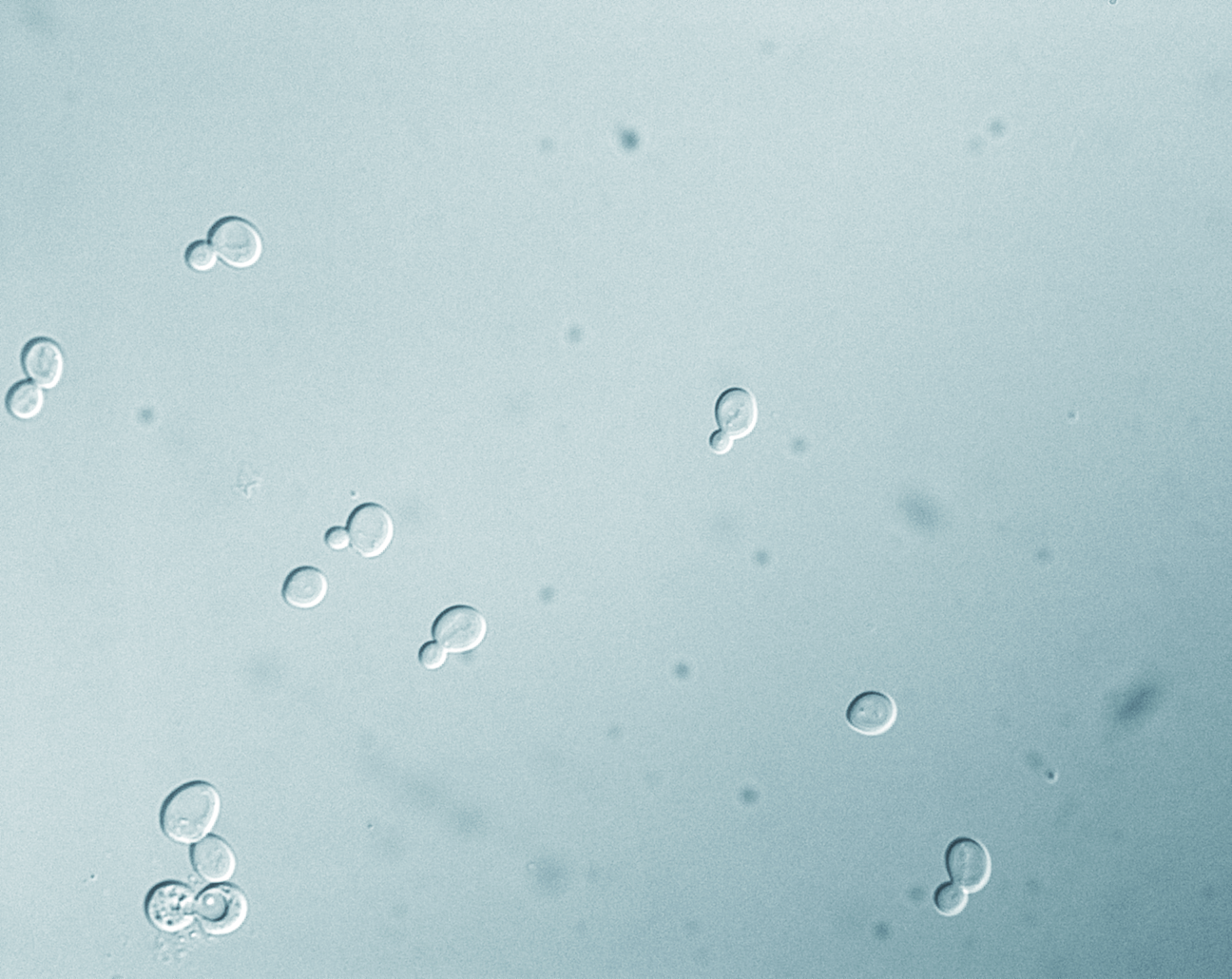

Supplement: Figure 3—source data 6. [file elife-91223-fig3-data6.zip › Figure 3C source data 4.tif]

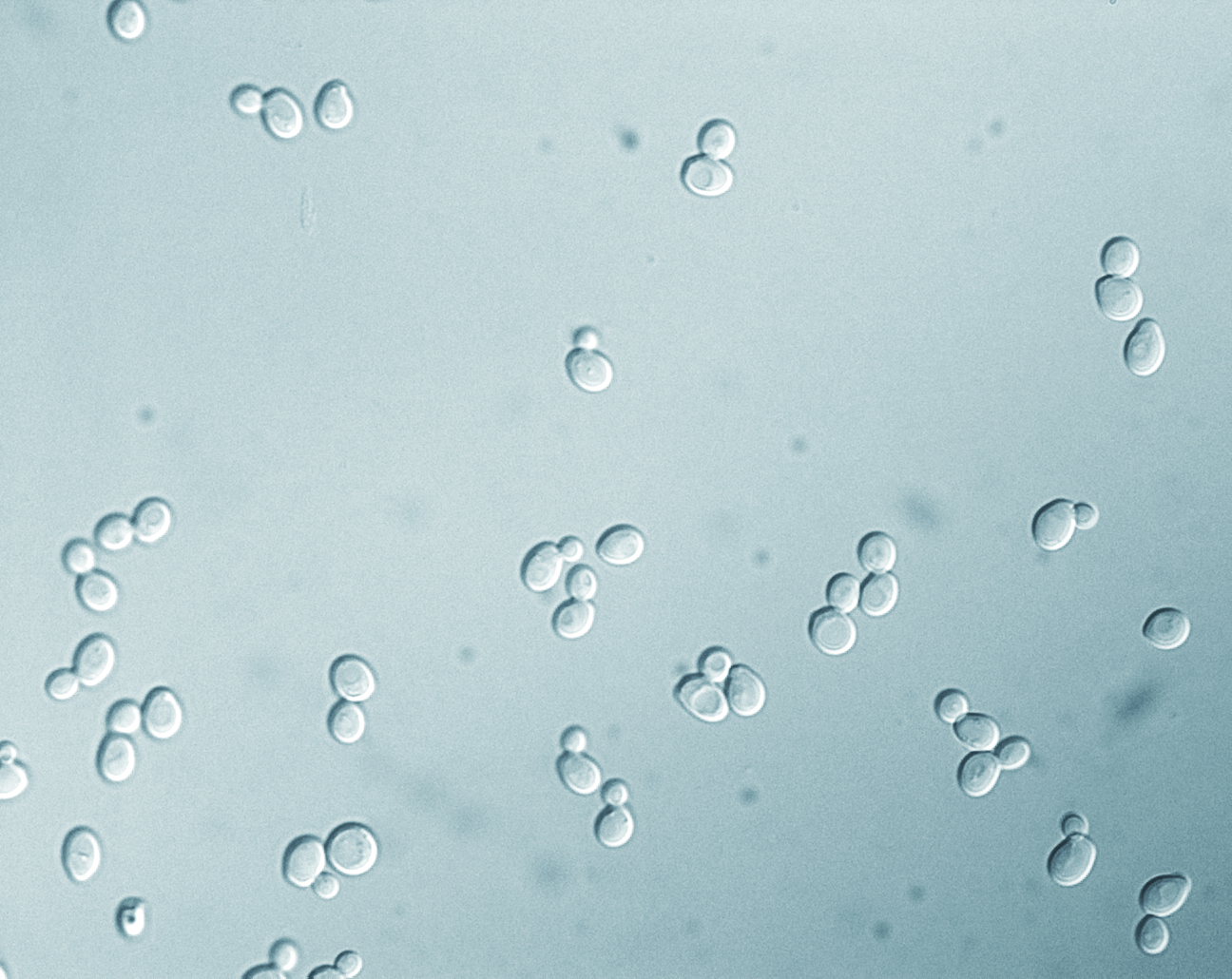

Supplement: Figure 3—source data 7. [file elife-91223-fig3-data7.zip › Figure 3C source data 5.tif]

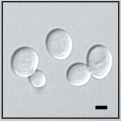

BY4742

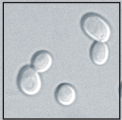

SY12

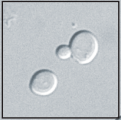

SY12<sup>XYΔ</sup>

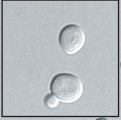

SY12<sup>YΔ</sup>

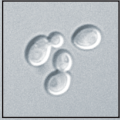

SY12<sup>XYΔ+Y</sup>

Supplement: Figure 3—source data 8. [file elife-91223-fig3-data8.zip › PDF containing Figure 3C and original photos of the strains.pdf]

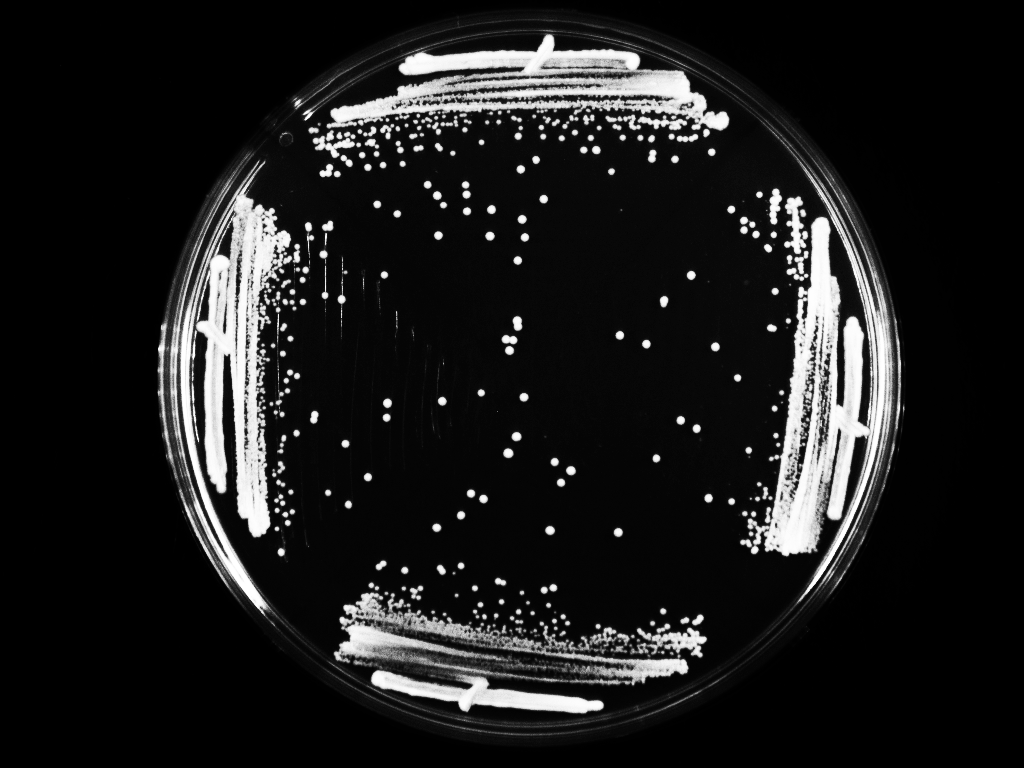

Supplement: Figure 3—source data 9. [file elife-91223-fig3-data9.zip › Figure 3D source data 1.tif]

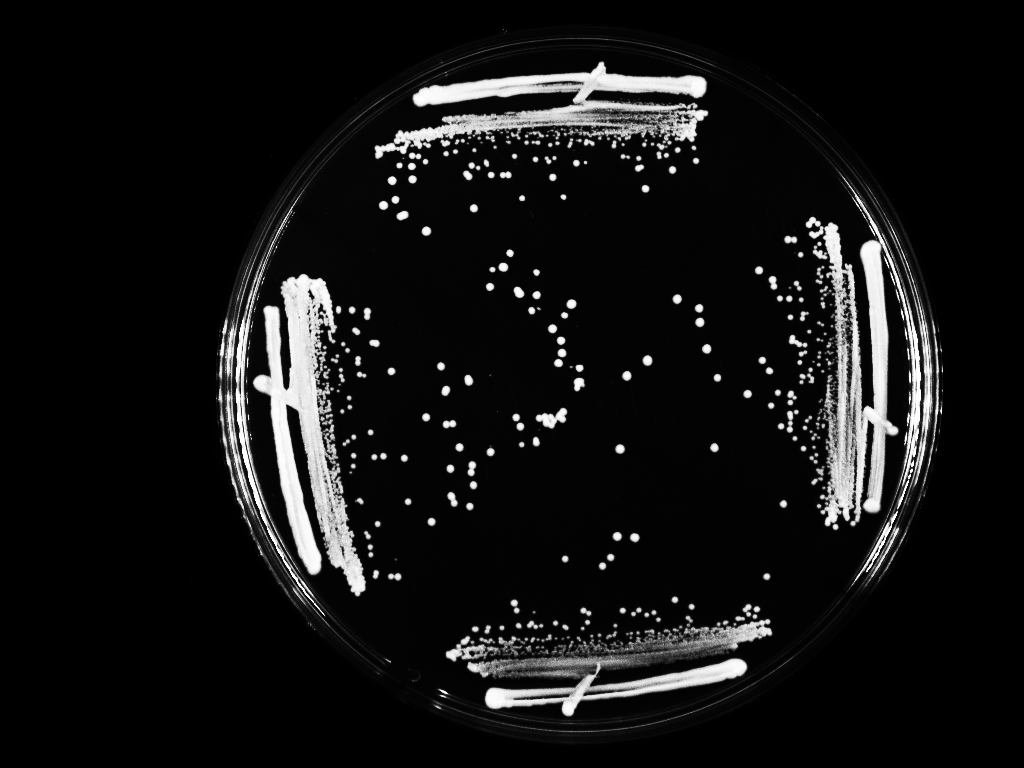

Supplement: Figure 3—source data 10. [file elife-91223-fig3-data10.zip › Figure 3D source data 2.tif]

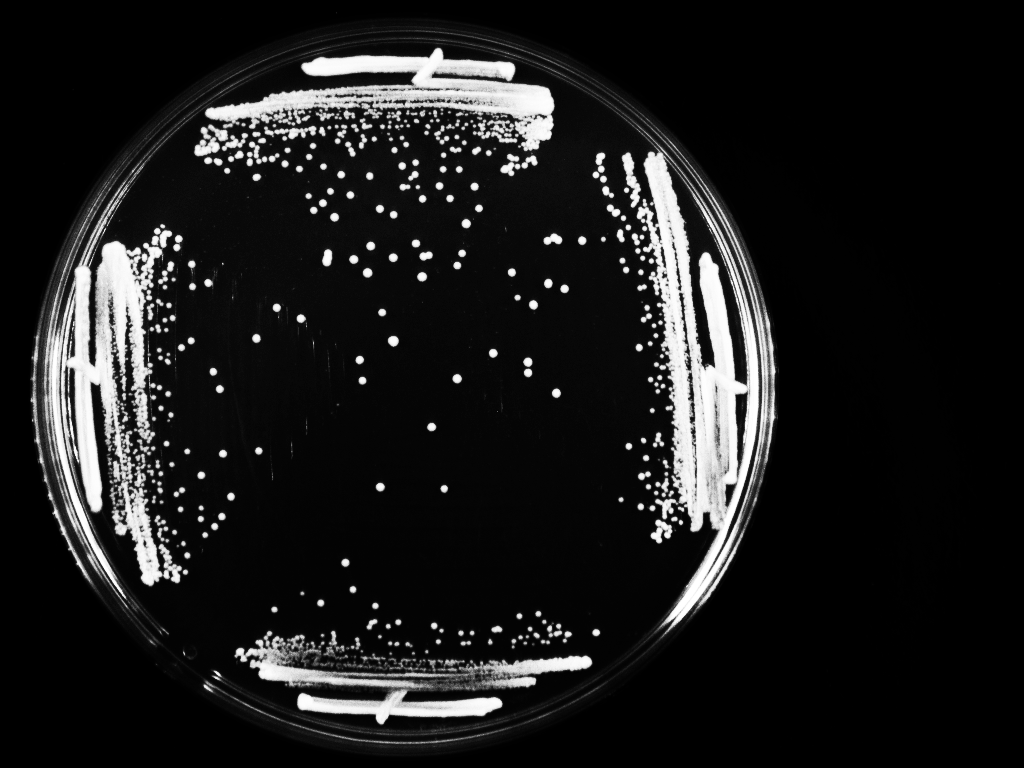

Supplement: Figure 3—source data 11. [file elife-91223-fig3-data11.zip › Figure 3D source data 3.tif]

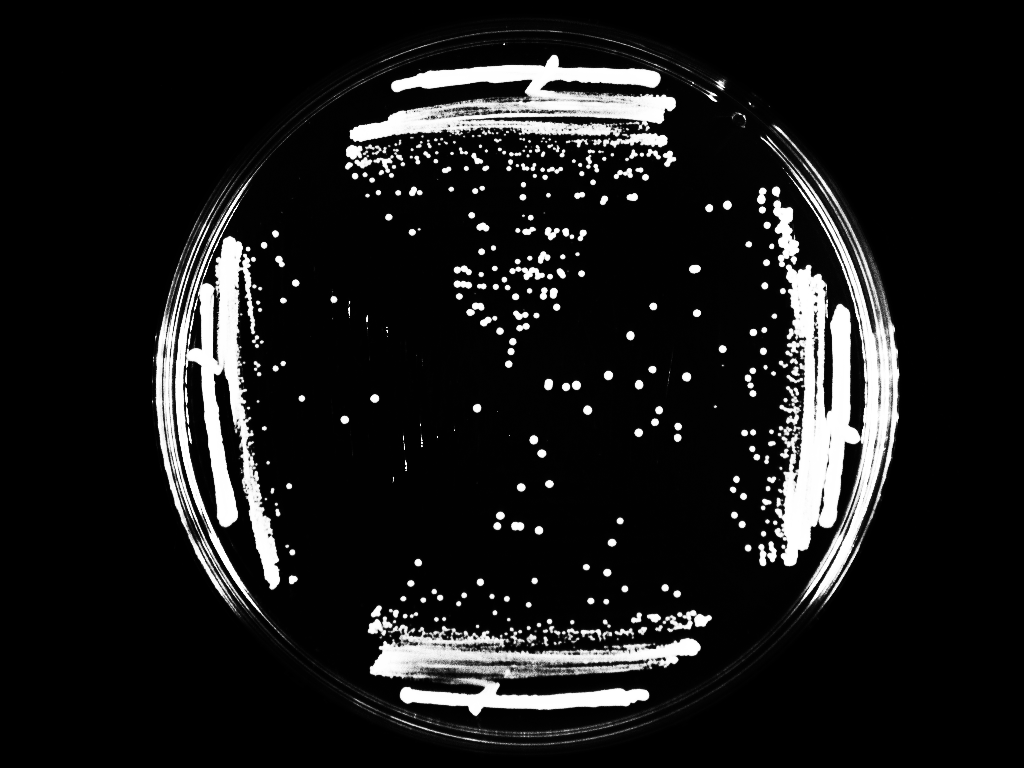

Supplement: Figure 3—source data 12. [file elife-91223-fig3-data12.zip › Figure 3D source data 4.tif]

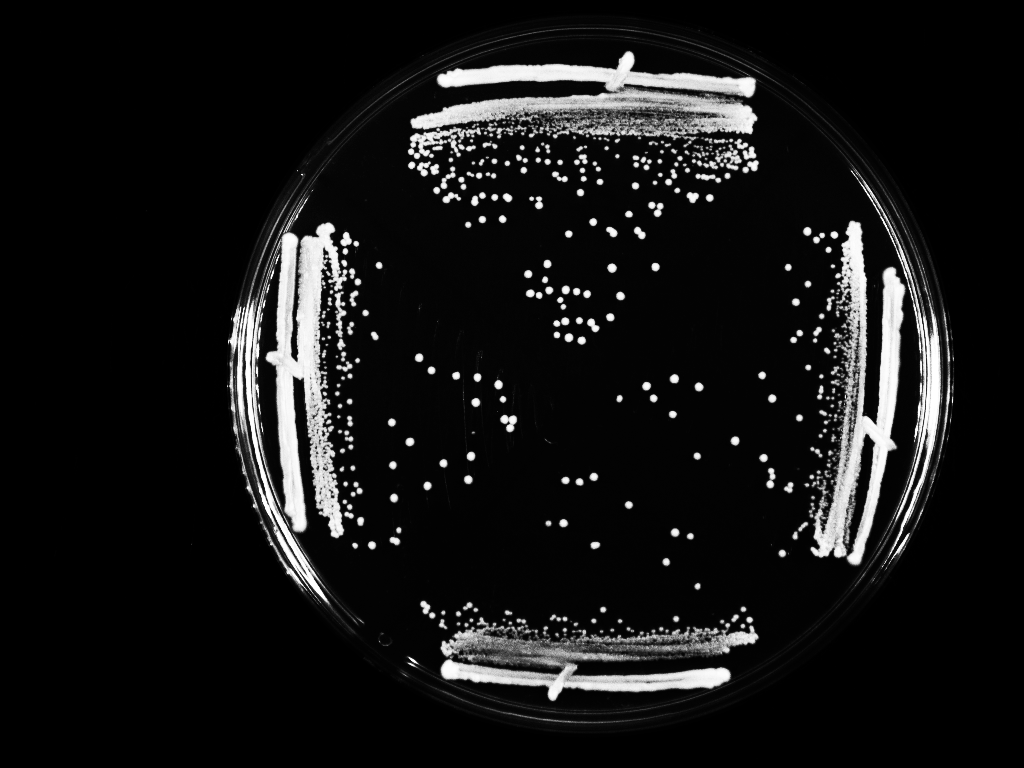

Supplement: Figure 3—source data 13. [file elife-91223-fig3-data13.zip › Figure 3D source data 5.tif]

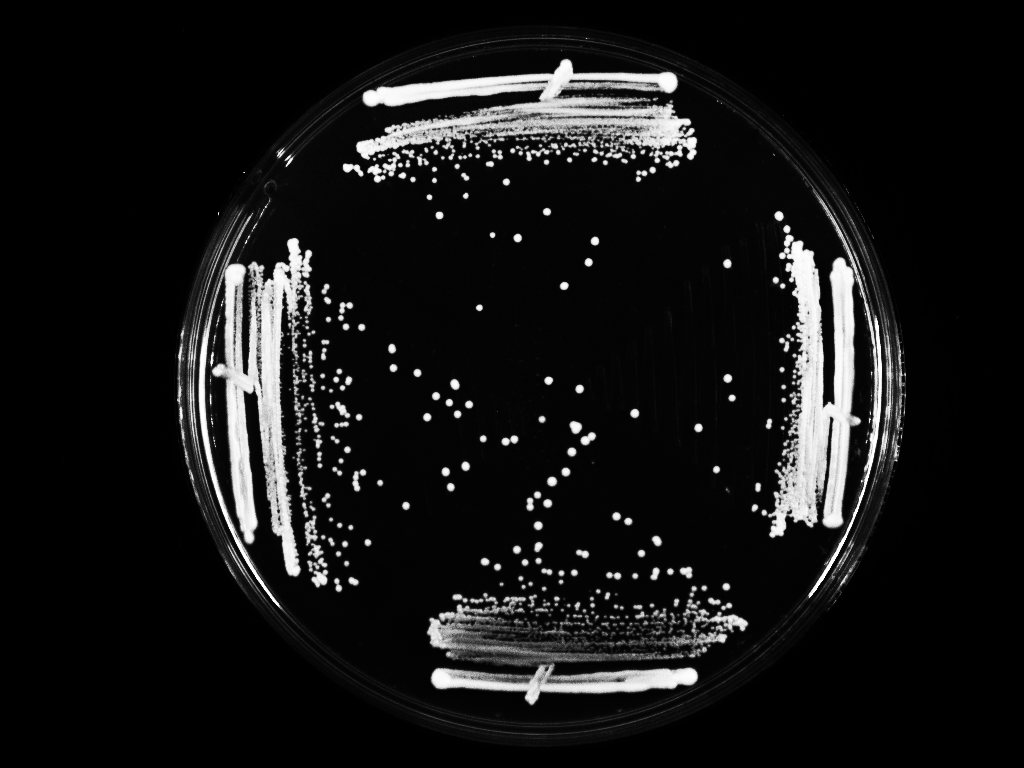

Supplement: Figure 3—source data 14. [file elife-91223-fig3-data14.zip › Figure 3D source data 6.tif]

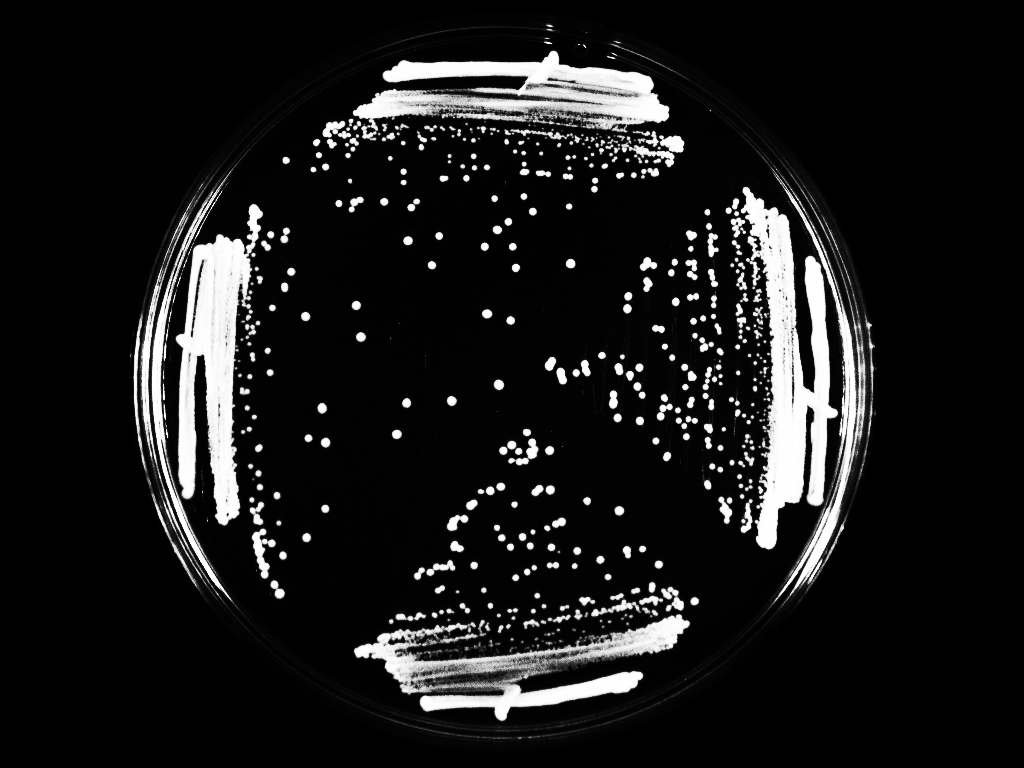

Supplement: Figure 3—source data 15. [file elife-91223-fig3-data15.zip › Figure 3D source data 7.tif]

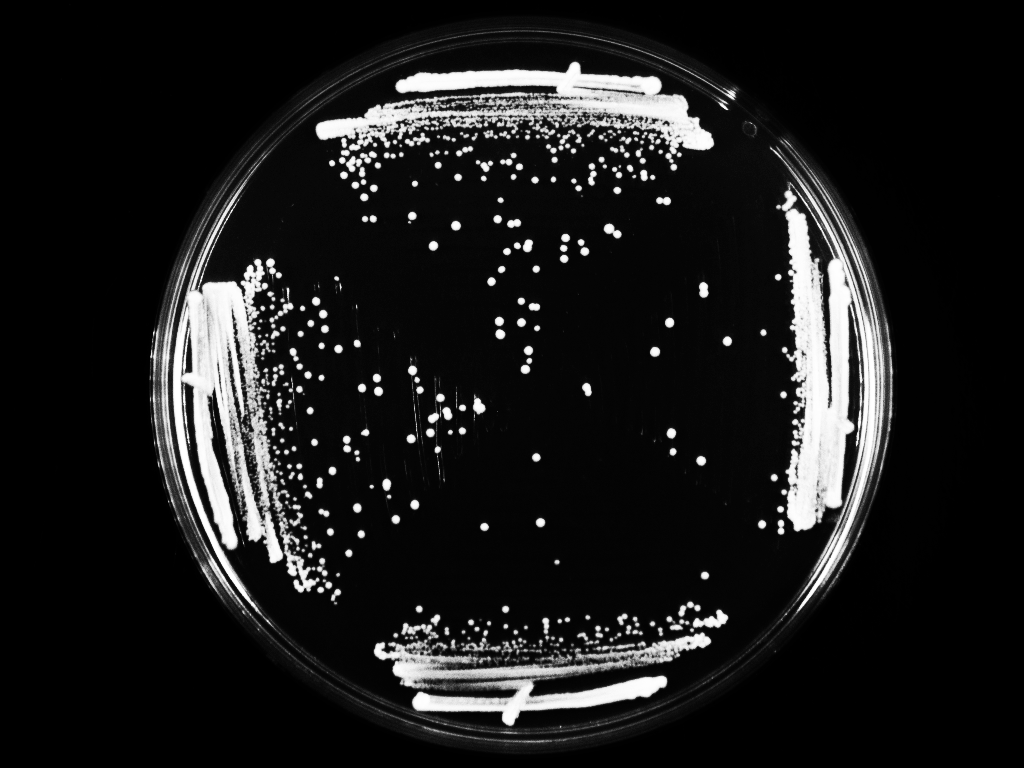

Supplement: Figure 3—source data 16. [file elife-91223-fig3-data16.zip › Figure 3D source data 8.tif]

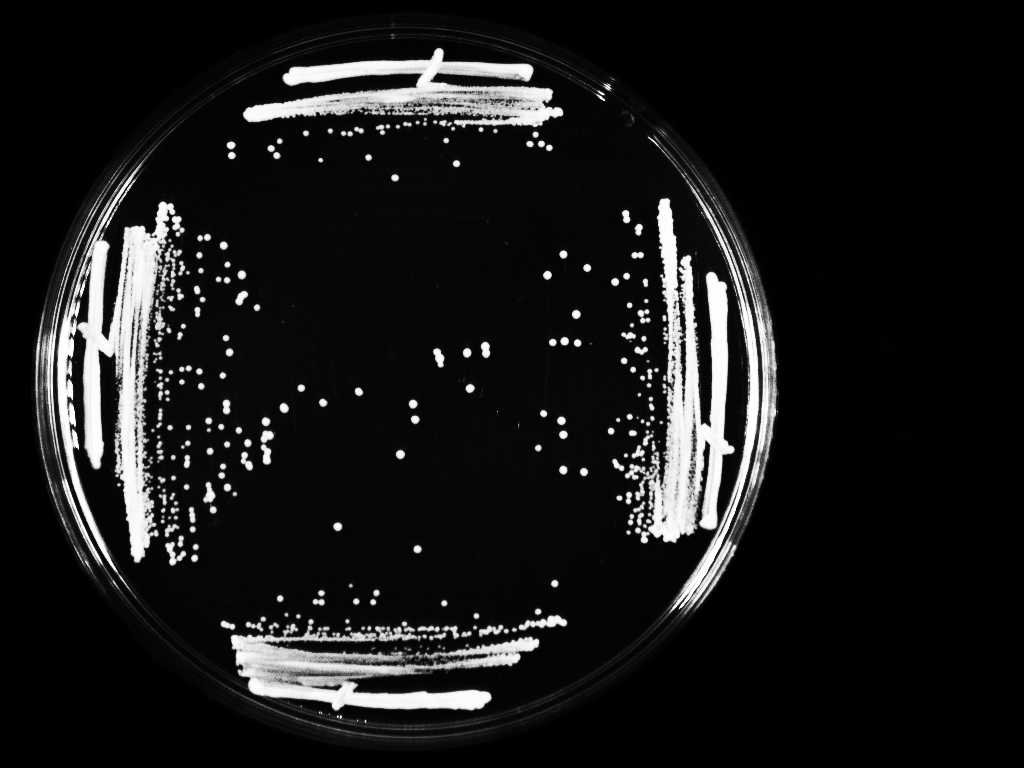

Supplement: Figure 3—source data 17. [file elife-91223-fig3-data17.zip › Figure 3D source data 9.tif]

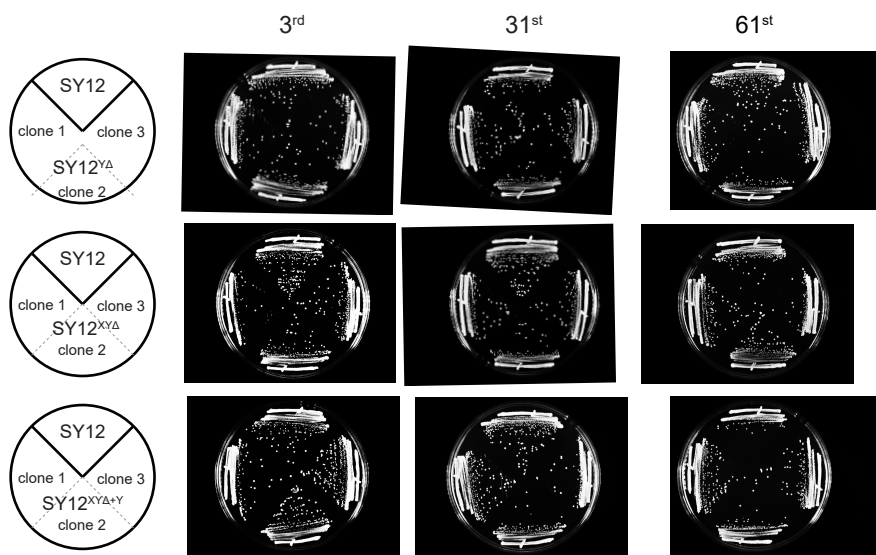

Supplement: Figure 3—source data 18. [file elife-91223-fig3-data18.zip › PDF containing Figure 3D and original scans of the relevant dotting blot analysis.pdf]

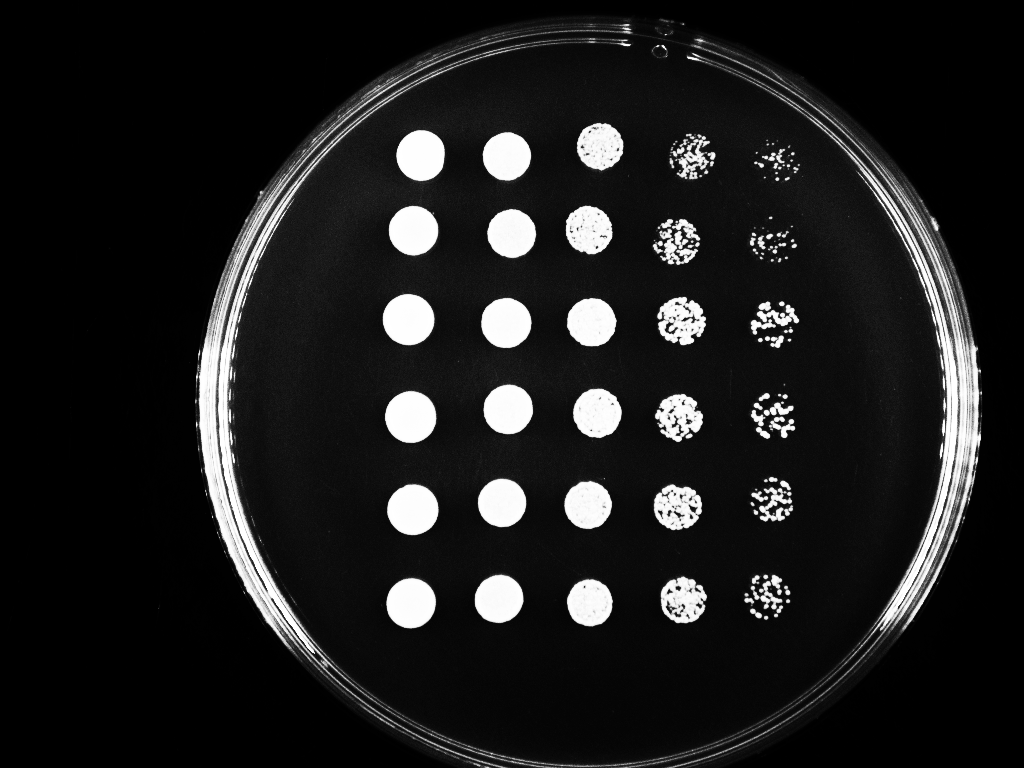

Supplement: Figure 3—source data 21. [file elife-91223-fig3-data21.zip › Figure 3G source data 1.tif]

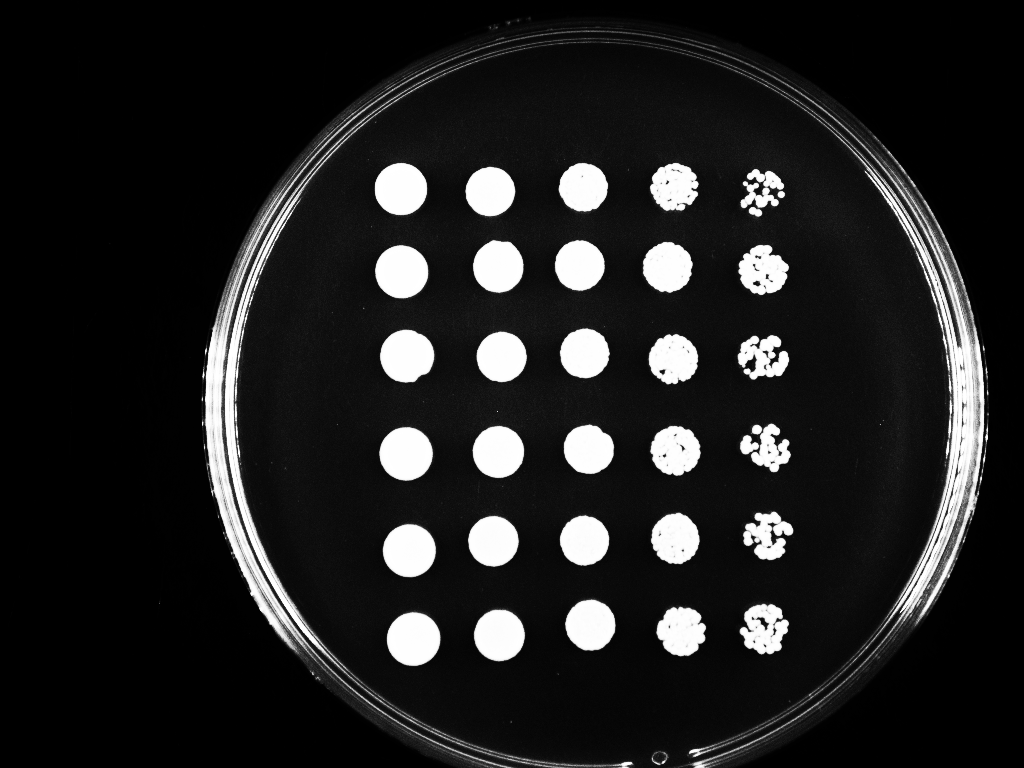

Supplement: Figure 3—source data 22. [file elife-91223-fig3-data22.zip › Figure 3G source data 2.tif]

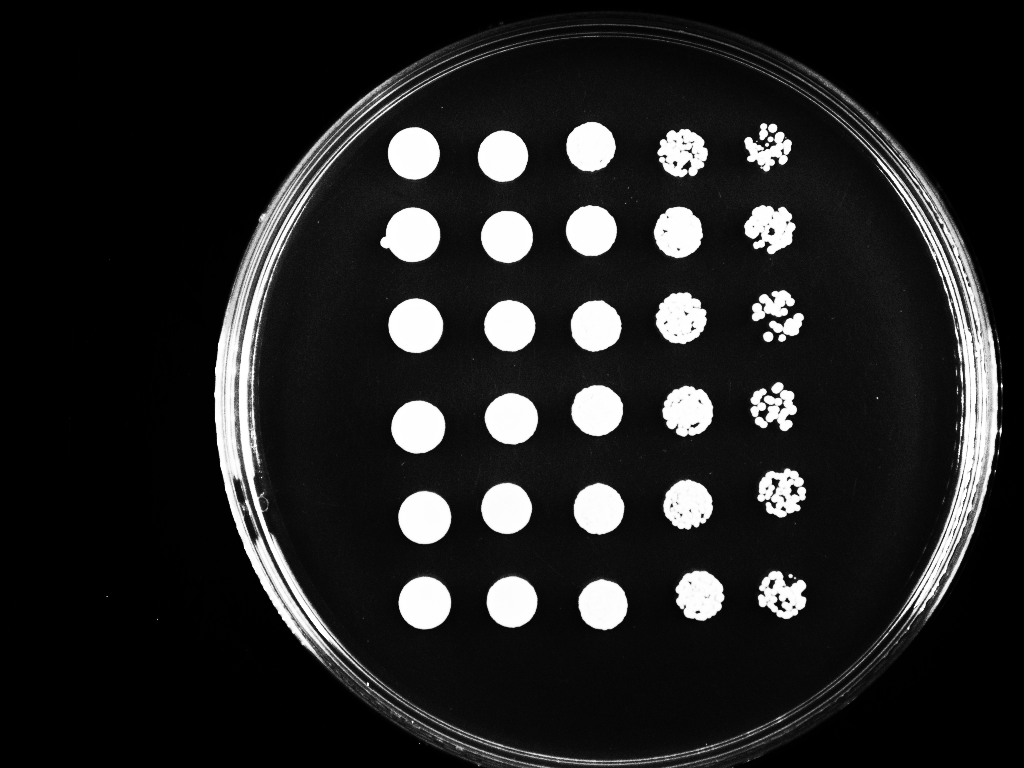

Supplement: Figure 3—source data 23. [file elife-91223-fig3-data23.zip › Figure 3G source data 3.tif]

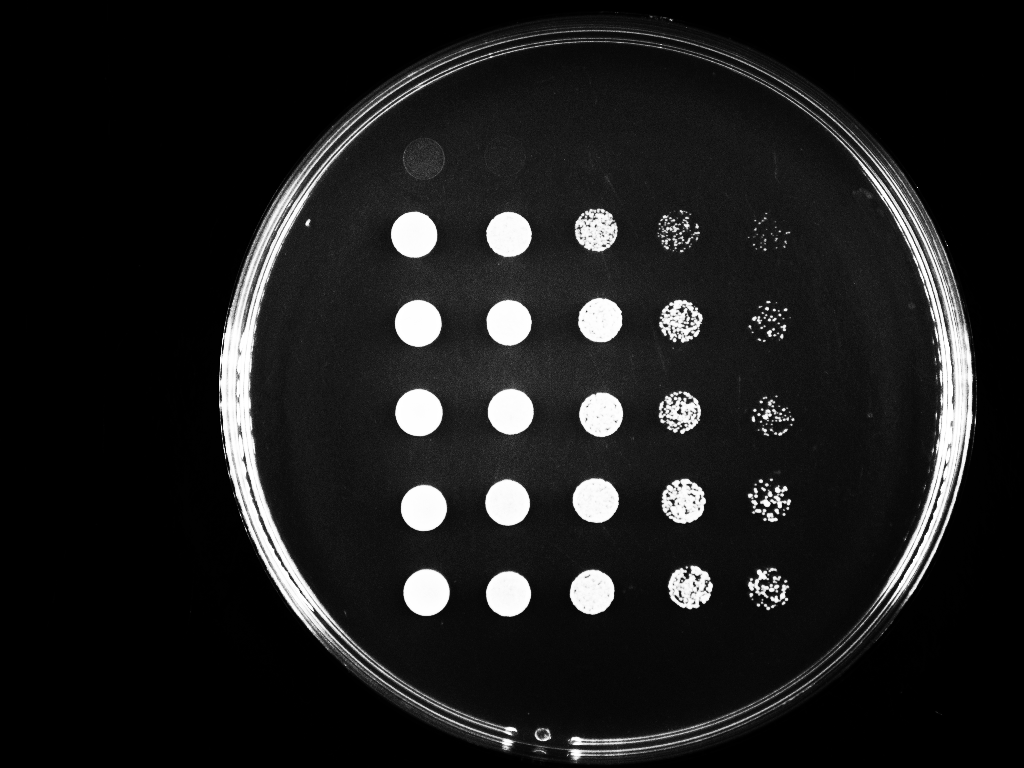

Supplement: Figure 3—source data 24. [file elife-91223-fig3-data24.zip › Figure 3G source data 4.tif]

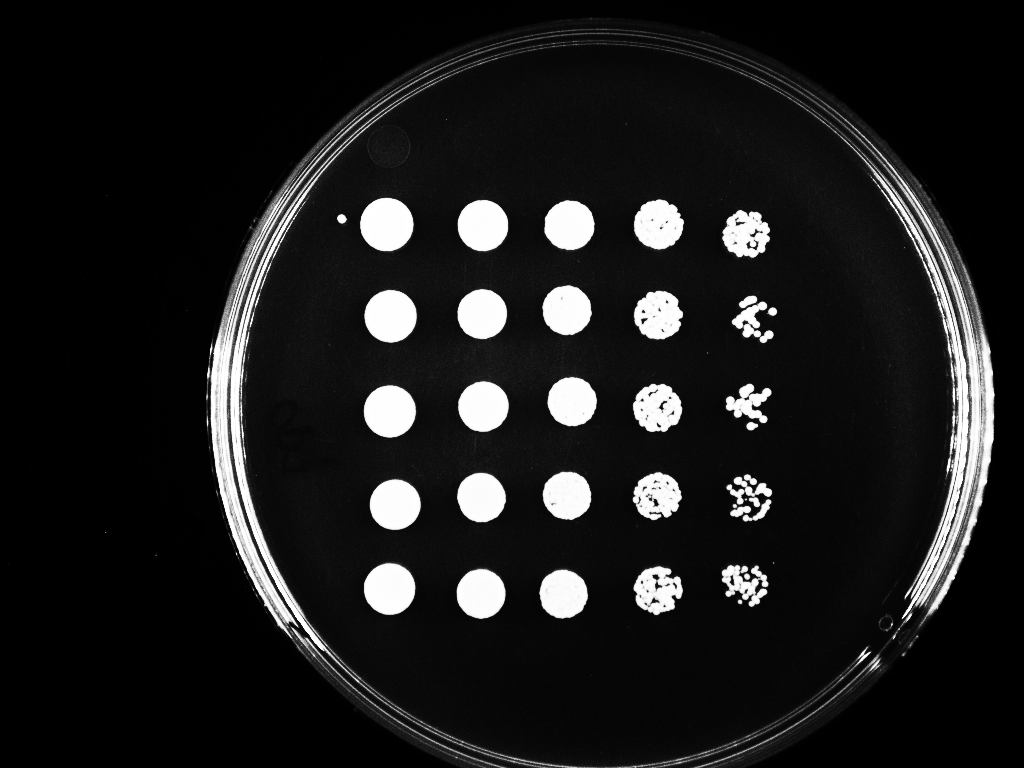

Supplement: Figure 3—source data 25. [file elife-91223-fig3-data25.zip › Figure 3G source data 5.tif]

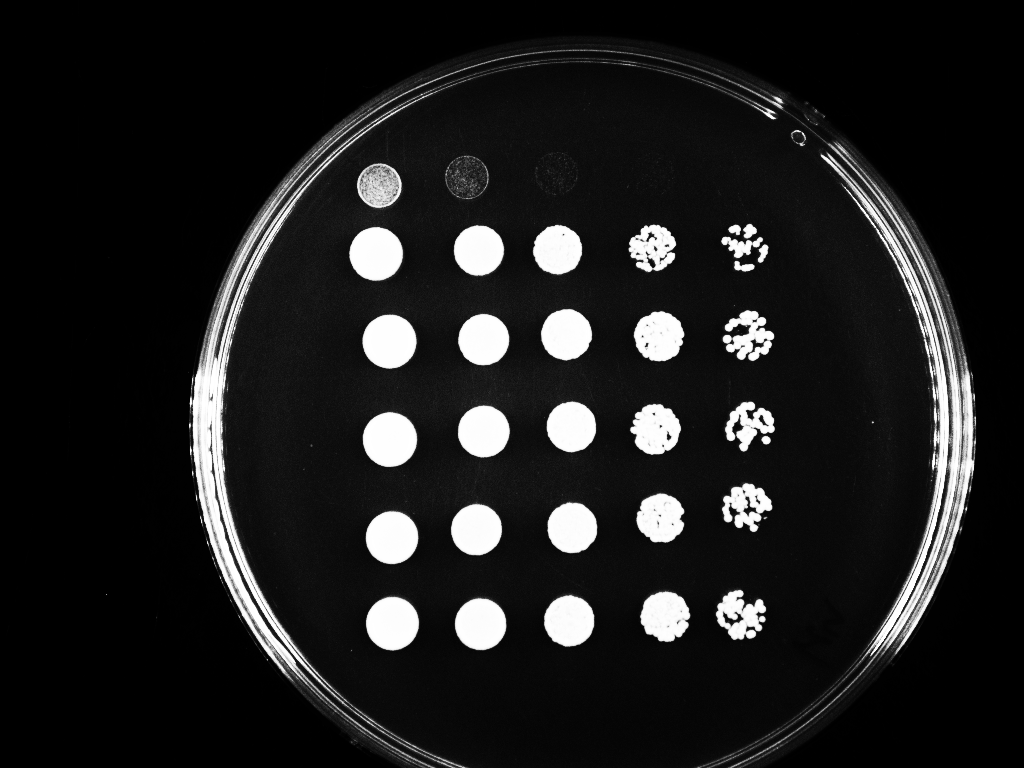

Supplement: Figure 3—source data 26. [file elife-91223-fig3-data26.zip › Figure 3G source data 6.tif]

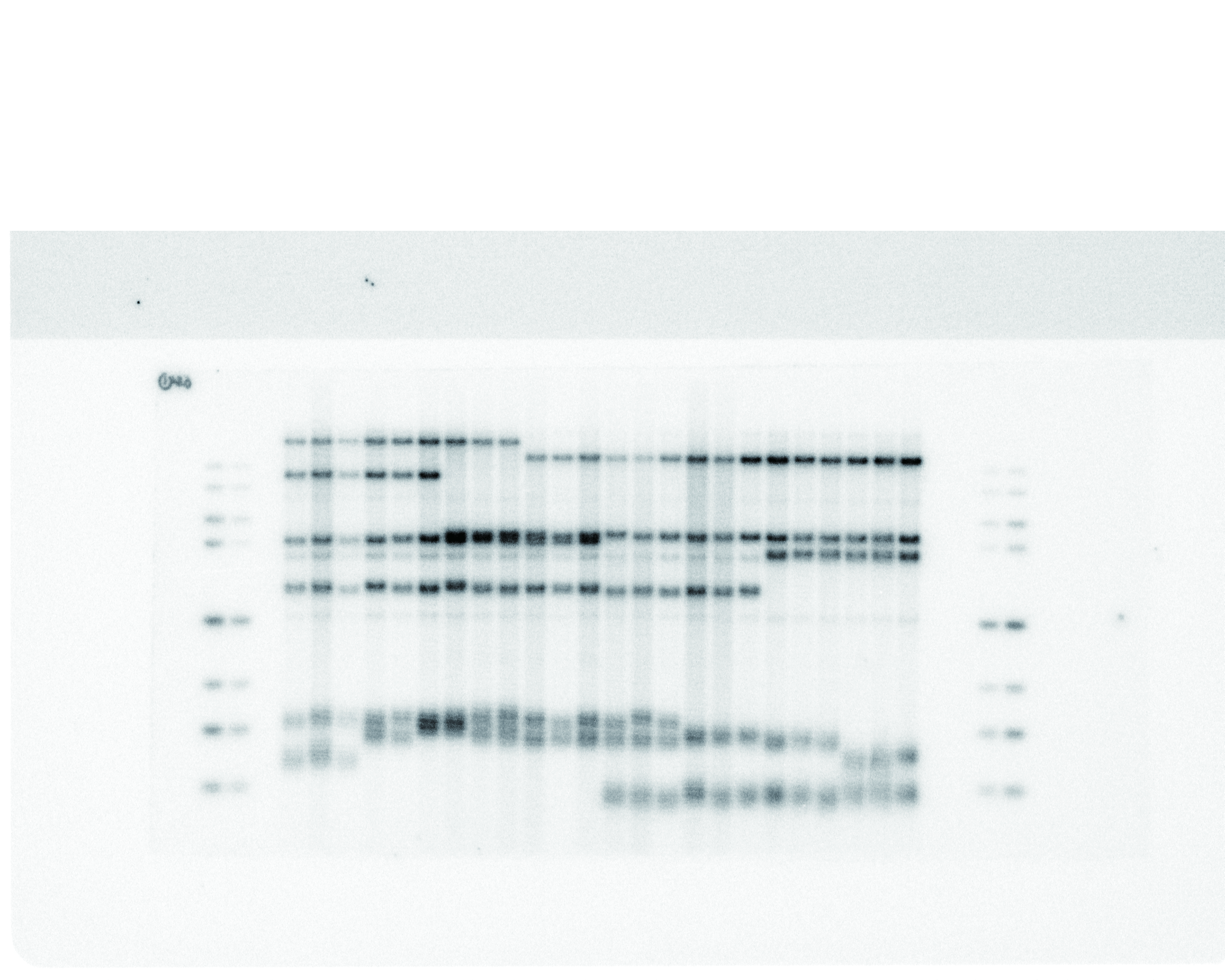

Supplement: Figure 4—source data 1. [file elife-91223-fig4-data1.zip › Figure 4A source data 1.tif]

A

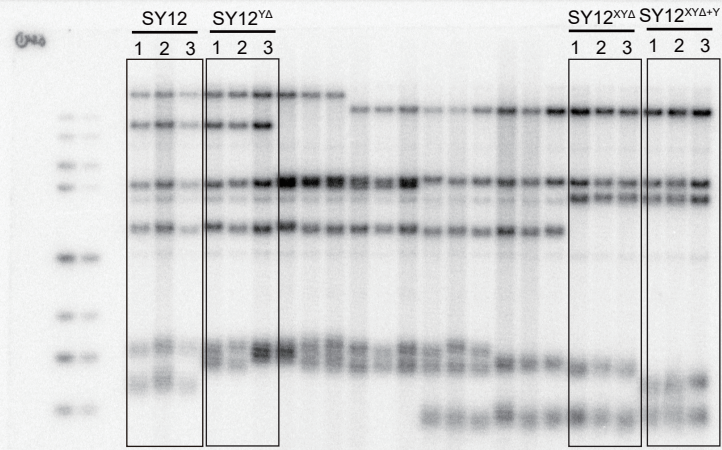

Supplement: Figure 4—source data 2. [file elife-91223-fig4-data2.zip › PDF containing Figure 4A and original scans of the relevant Southern blot analysis.pdf]

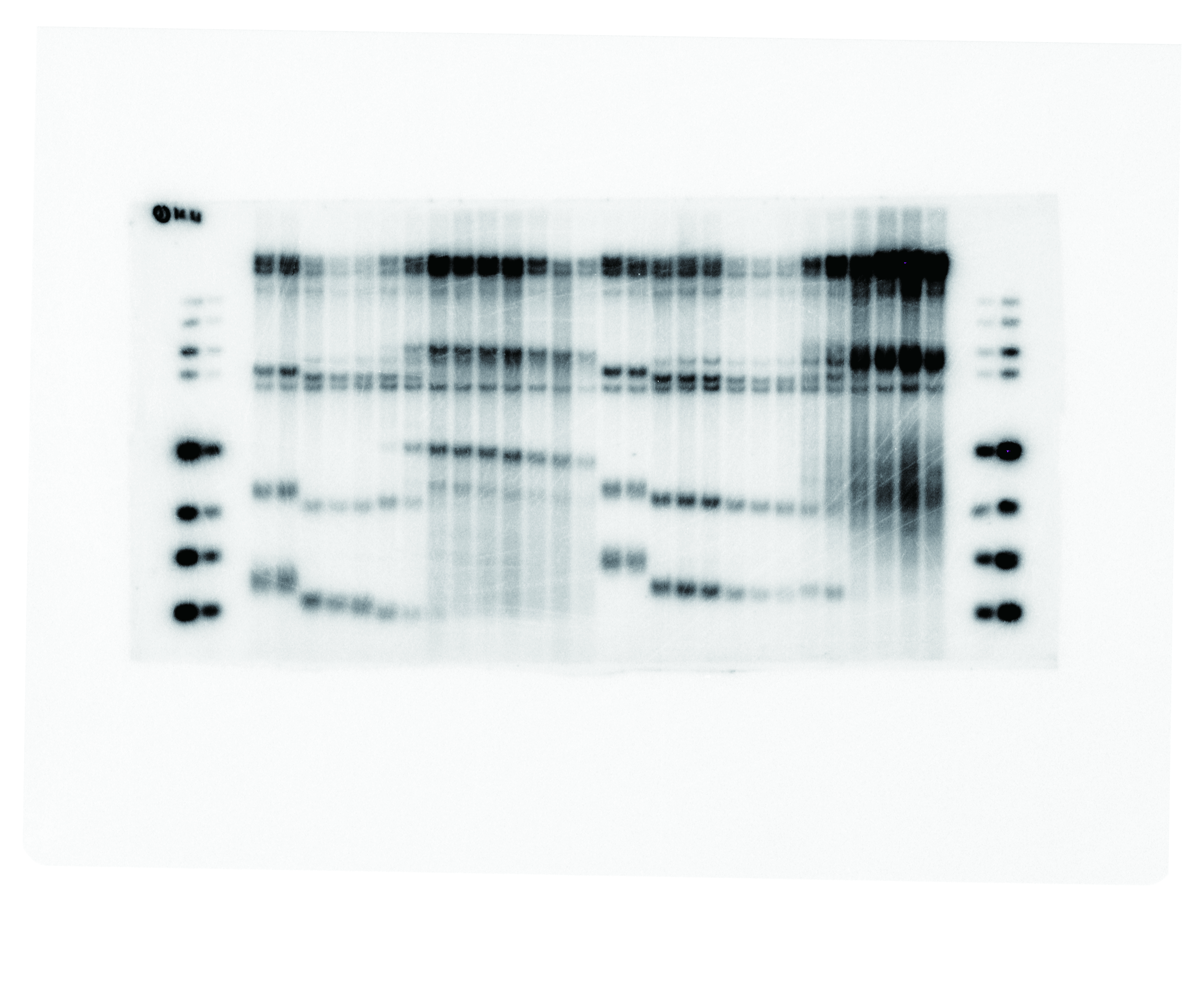

Supplement: Figure 5—source data 2. [file elife-91223-fig5-data2.zip › Figure 5B source data1.tif]

**B**

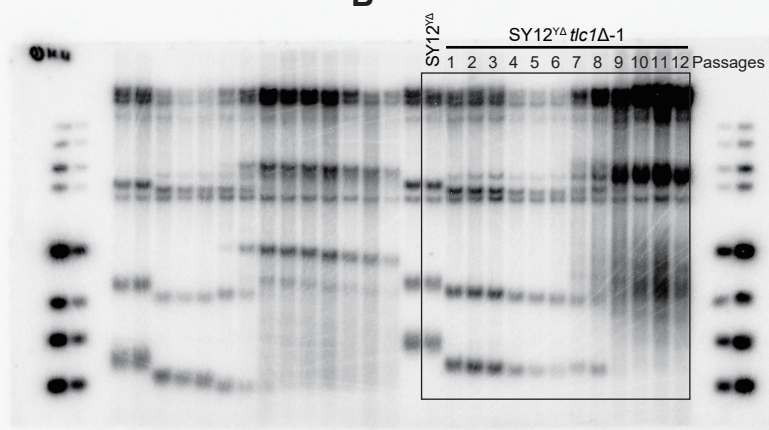

Supplement: Figure 5—source data 3. [file elife-91223-fig5-data3.zip › PDF containing Figure 5B and original scans of the relevant Southern blot analysis.pdf]

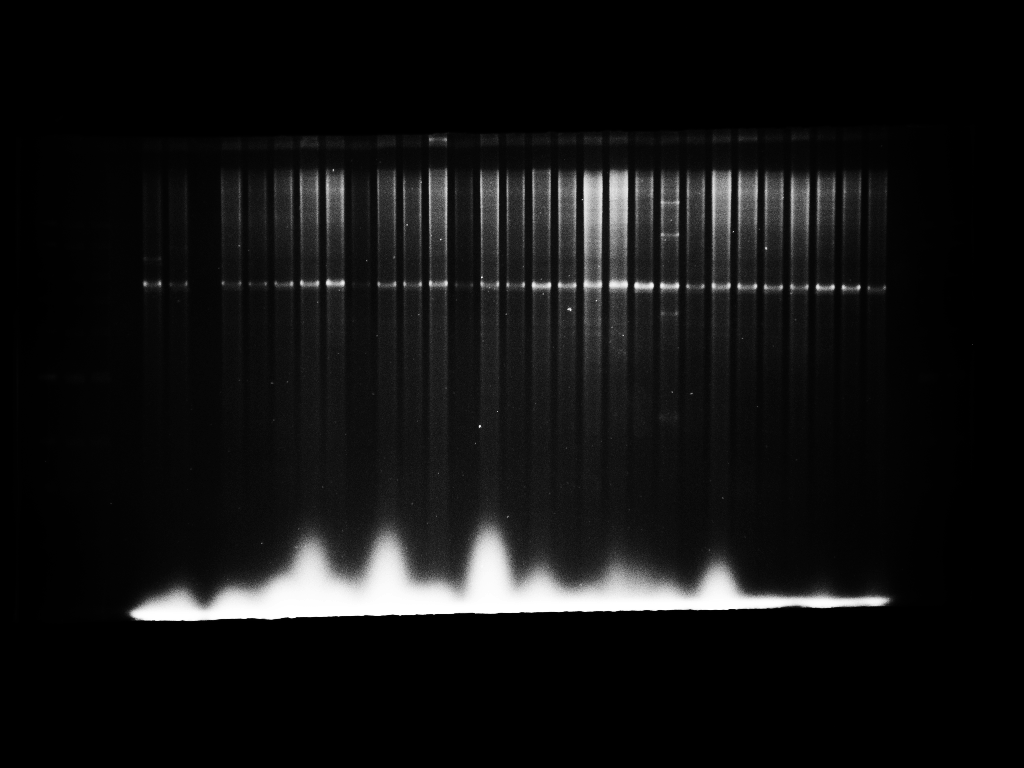

Supplement: Figure 5—source data 6. [file elife-91223-fig5-data6.zip › Figure 5C source data 3.tif]

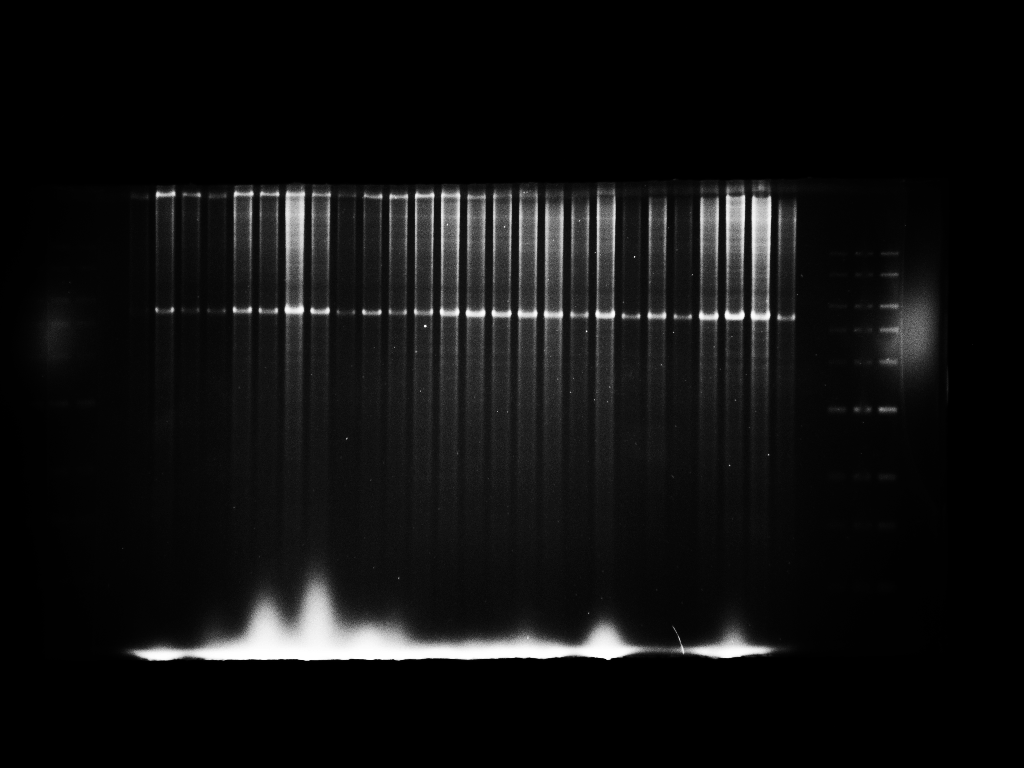

Supplement: Figure 5—source data 7. [file elife-91223-fig5-data7.zip › Figure 5C source data 4.tif]

C

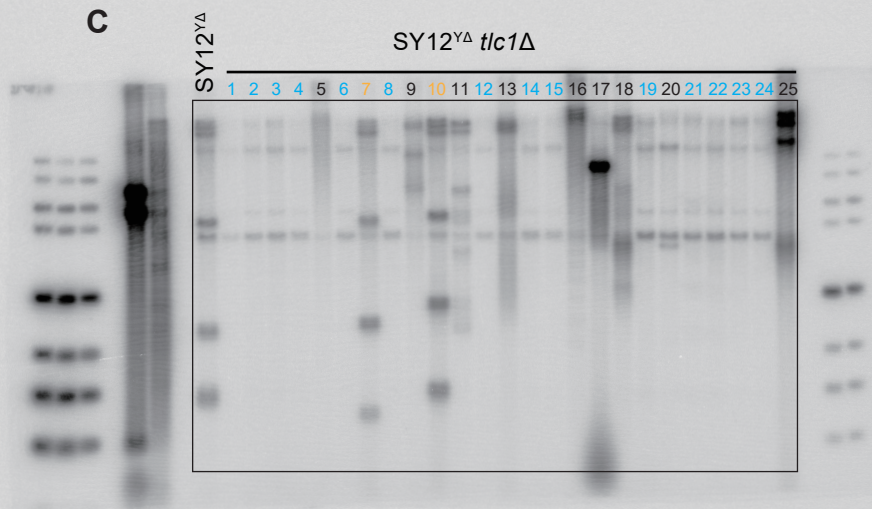

SY12<sup>YΔ</sup> *tlc1Δ*

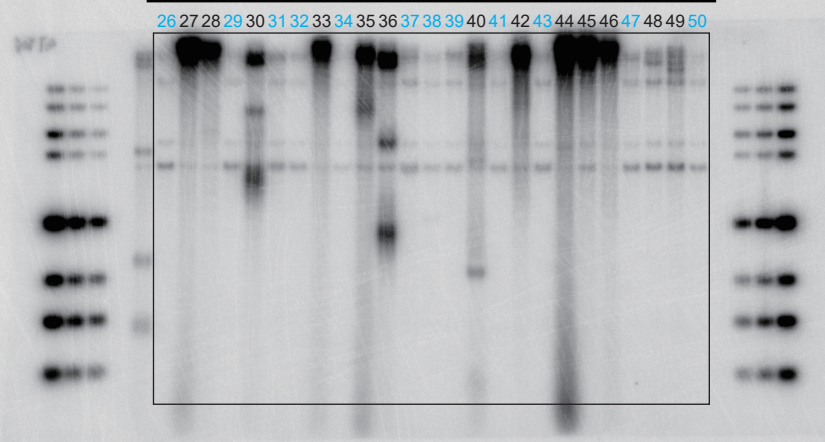

Supplement: Figure 5—source data 8. [file elife-91223-fig5-data8.zip › PDF containing Figure 5C and original scans of the relevant Southern blot analysis.pdf]

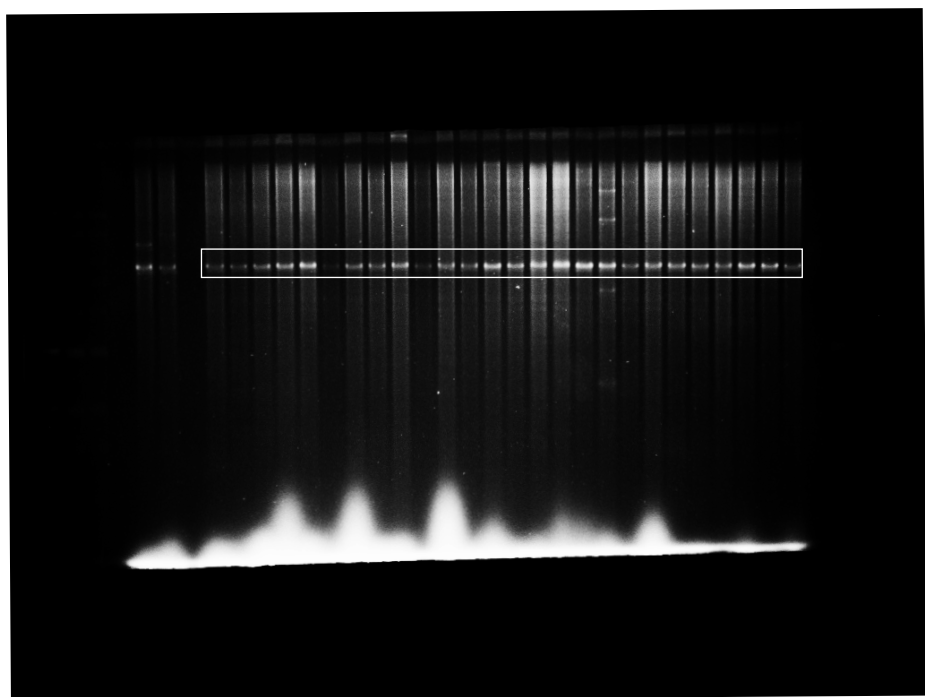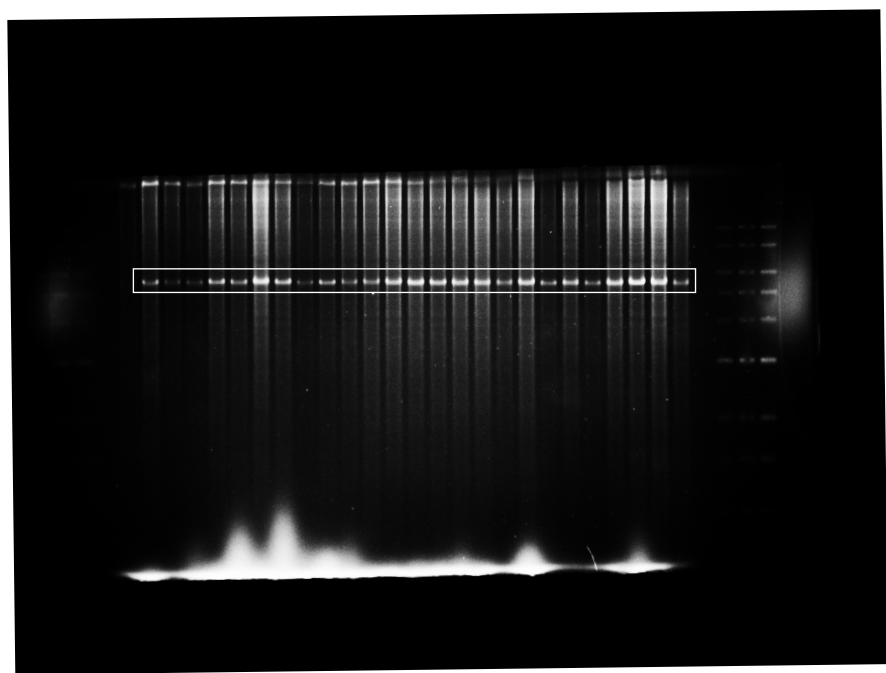

Supplement: Figure 5—source data 9. [file elife-91223-fig5-data9.zip › PDF containing original scans of the loading contral in Figure 5C.pdf]

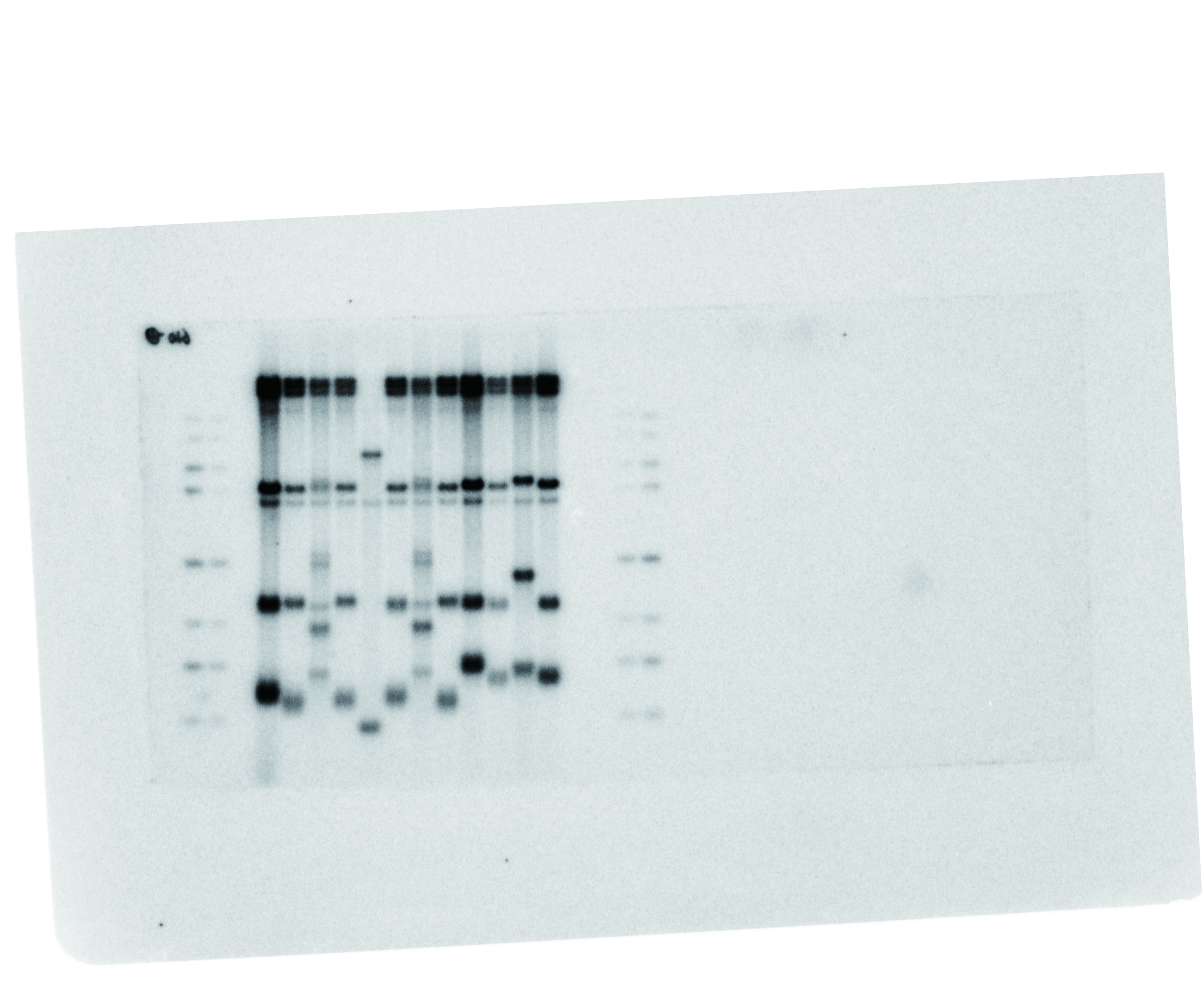

Supplement: Figure 5—figure supplement 1—source data 1. [file elife-91223-fig5-figsupp1-data1.zip › Figure5-figure supplementary1A- source data1.tif]

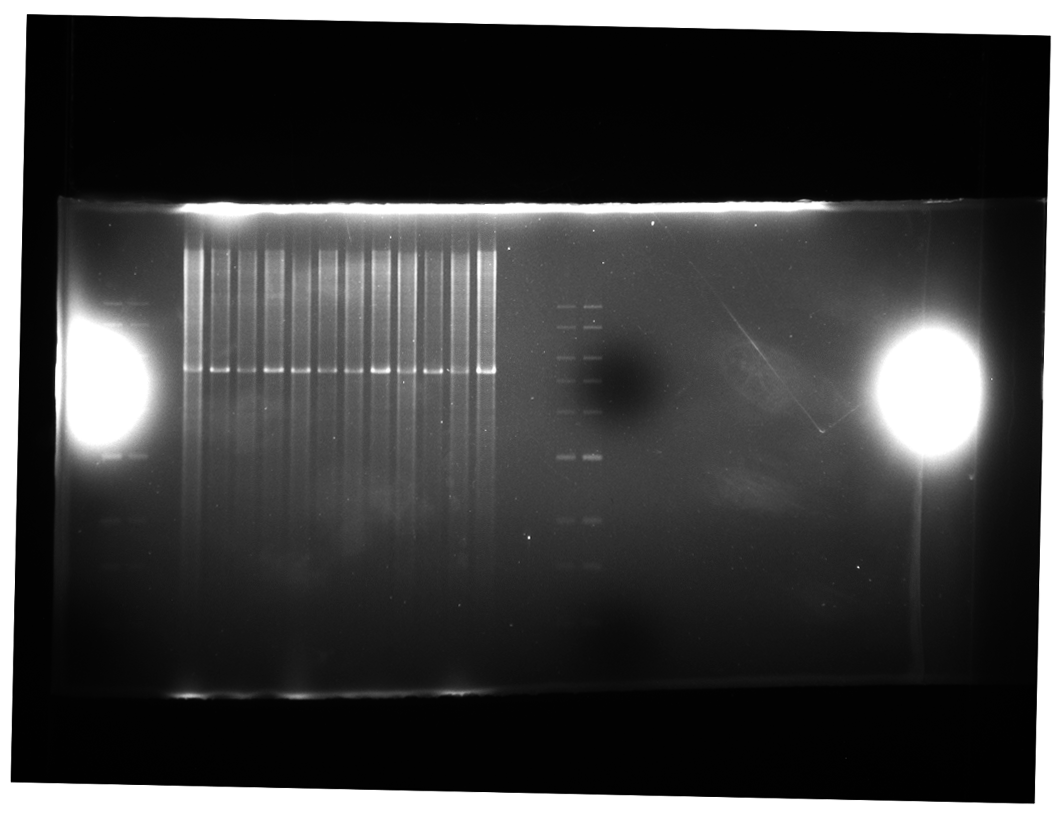

Supplement: Figure 5—figure supplement 1—source data 2. [file elife-91223-fig5-figsupp1-data2.zip › Figure5-figure supplementary1A- source data2.tif]

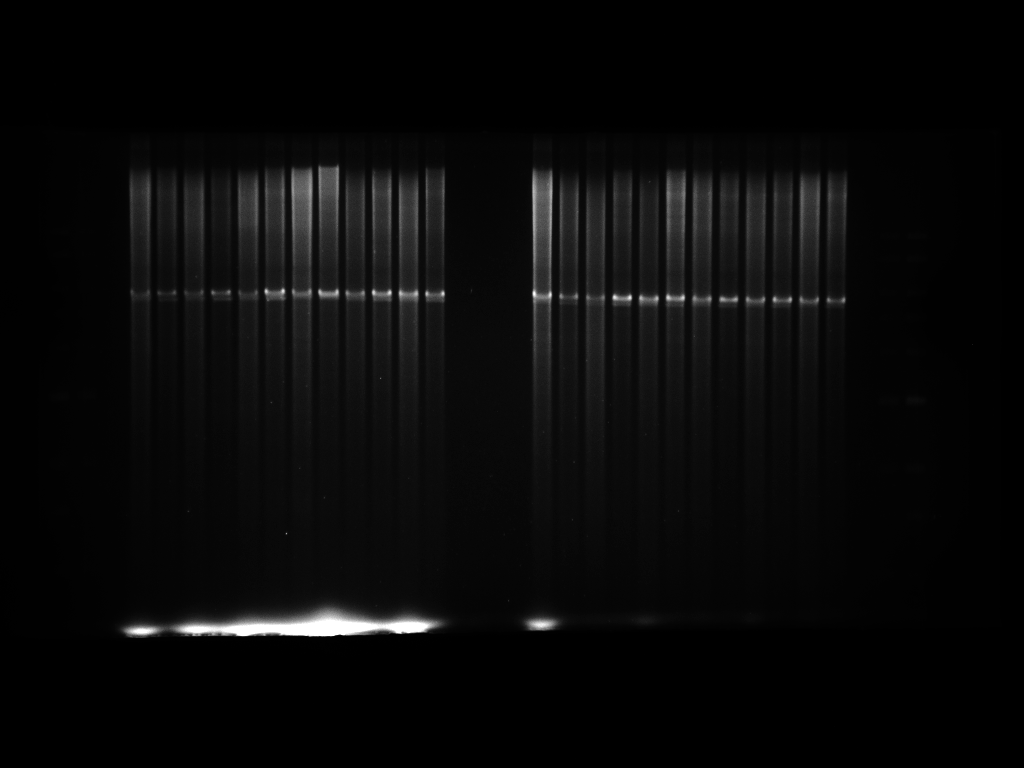

Supplement: Figure 5—figure supplement 1—source data 4. [file elife-91223-fig5-figsupp1-data4.zip › Figure5-figure supplementary1B- source data2.tif]

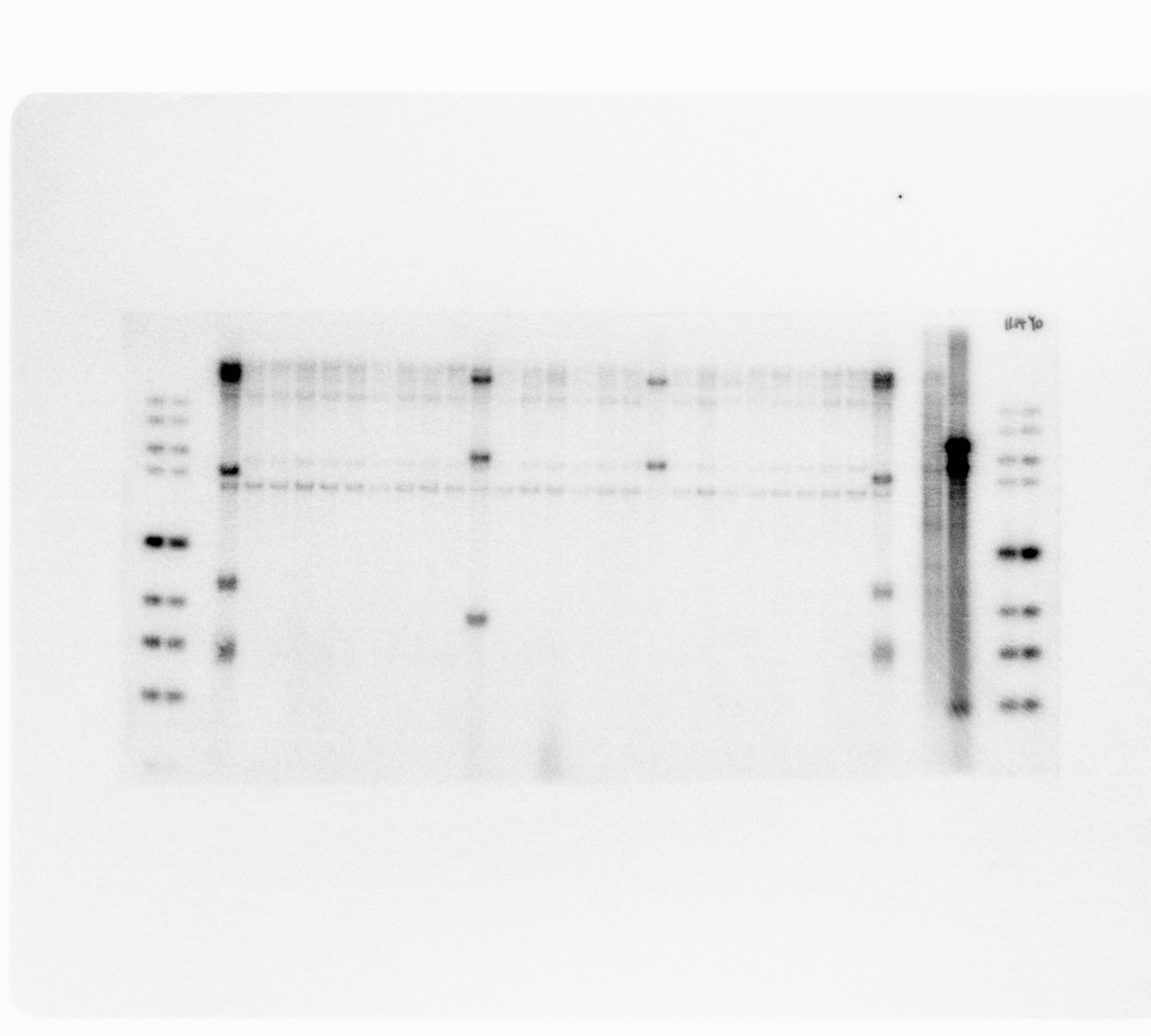

Supplement: Figure 5—figure supplement 3—source data 2. [file elife-91223-fig5-figsupp3-data2.zip › Figure5-figure supplementary 3- source data2.tif]

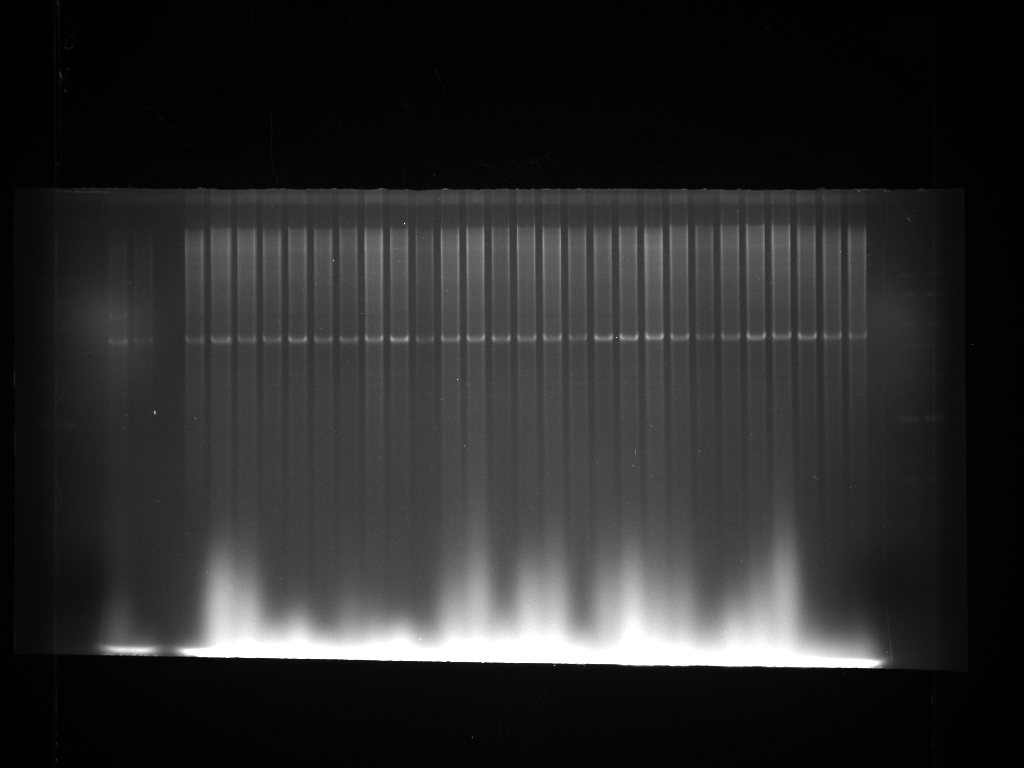

Supplement: Figure 5—figure supplement 3—source data 3. [file elife-91223-fig5-figsupp3-data3.zip › Figure5-figure supplementary3B- source data2.Tif]

**B**

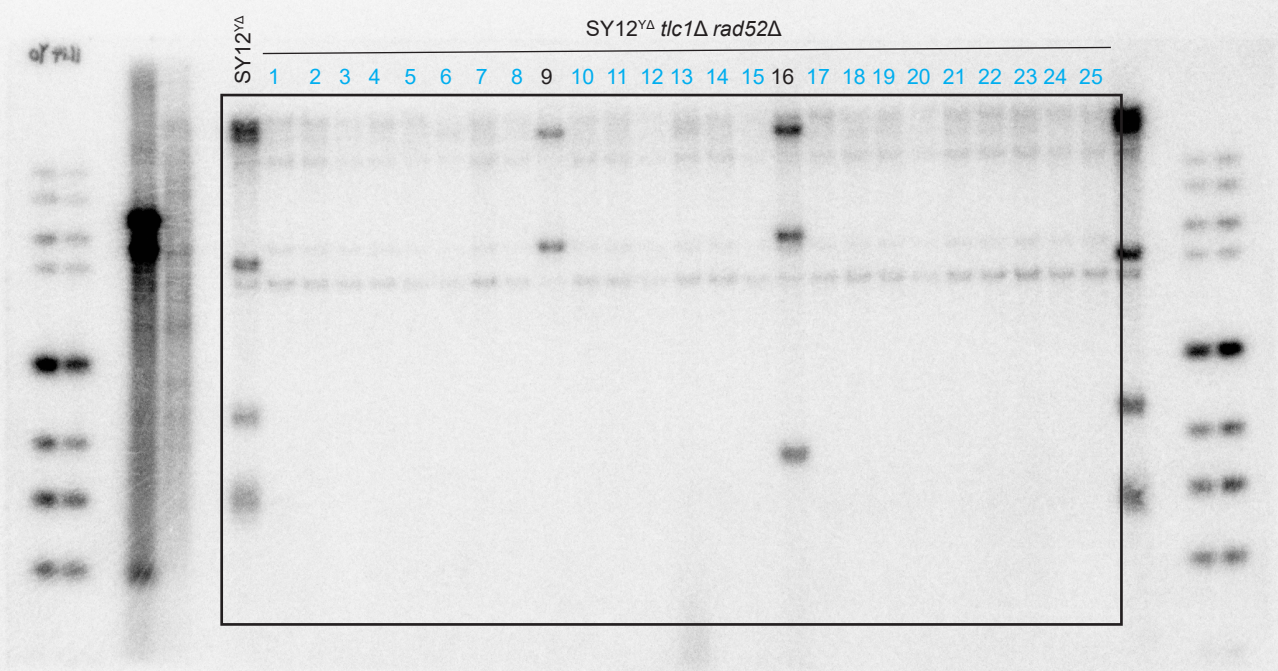

Supplement: Figure 5—figure supplement 3—source data 4. [file elife-91223-fig5-figsupp3-data4.zip › PDF containing Figure 5-figure supplementary3 and original scans of the relevant Southern blot analysis.pdf]

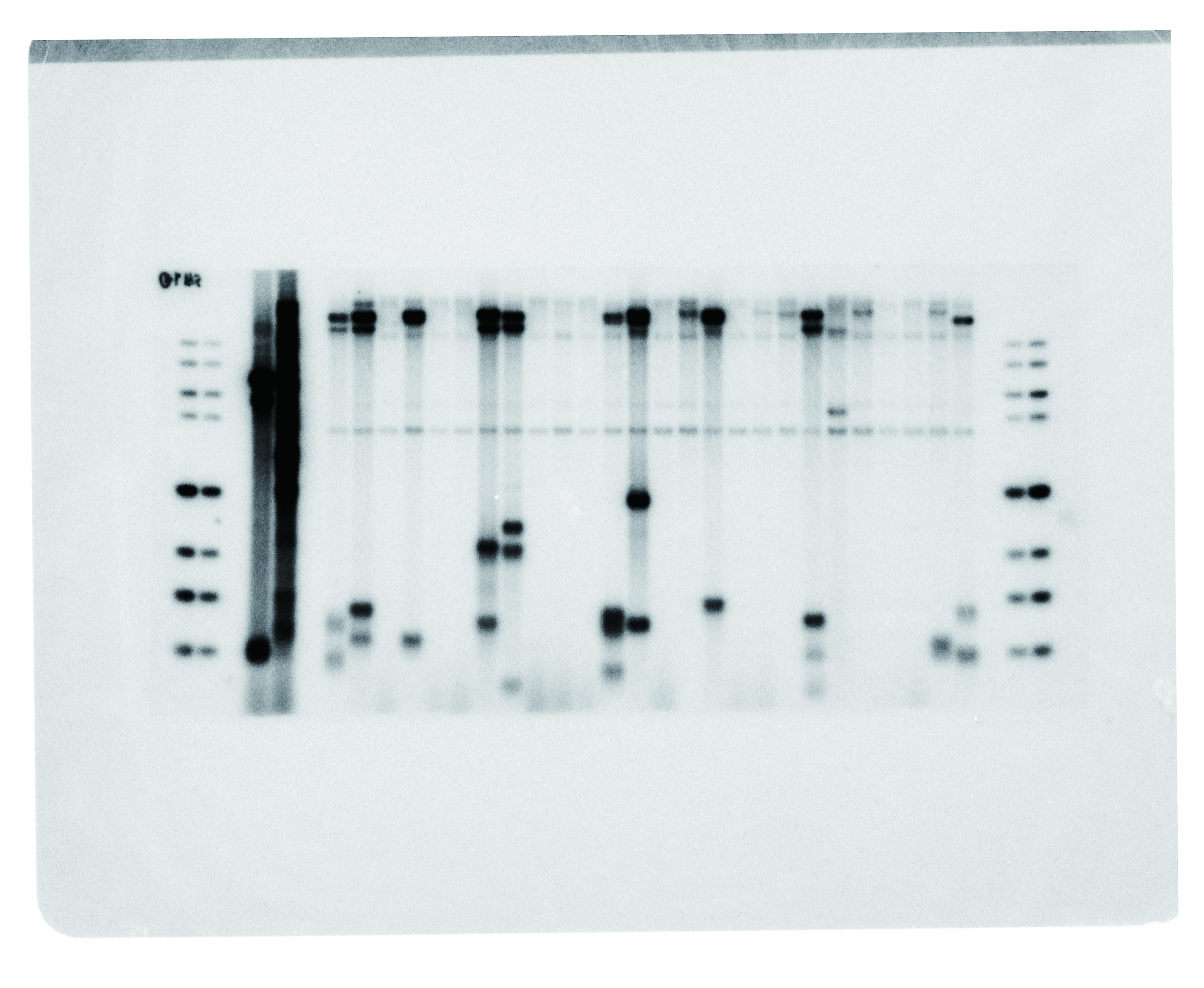

Supplement: Figure 6—source data 1. [file elife-91223-fig6-data1.zip › Figure 6A source data1.tif]

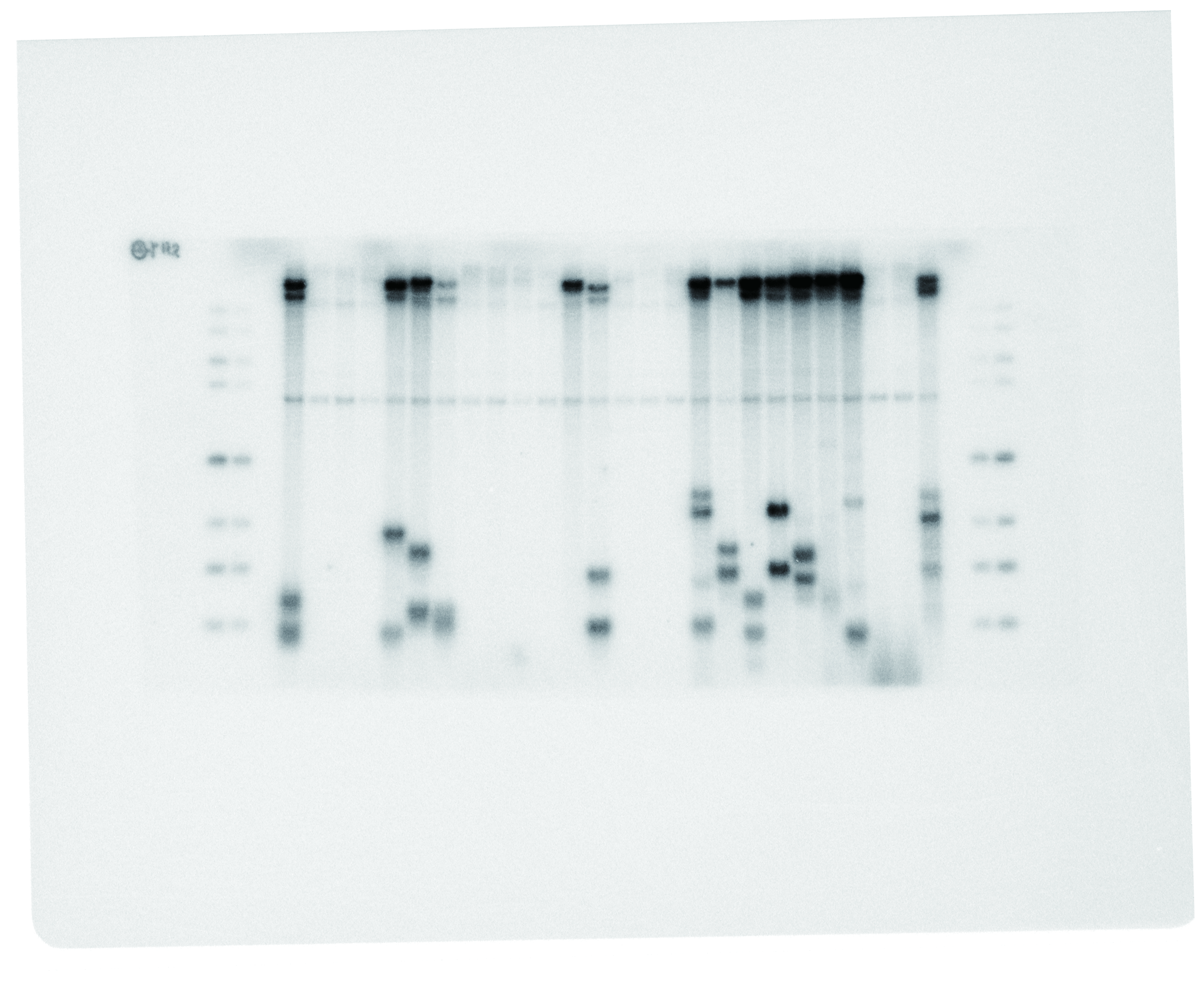

Supplement: Figure 6—source data 2. [file elife-91223-fig6-data2.zip › Figure 6A source data2.tif]

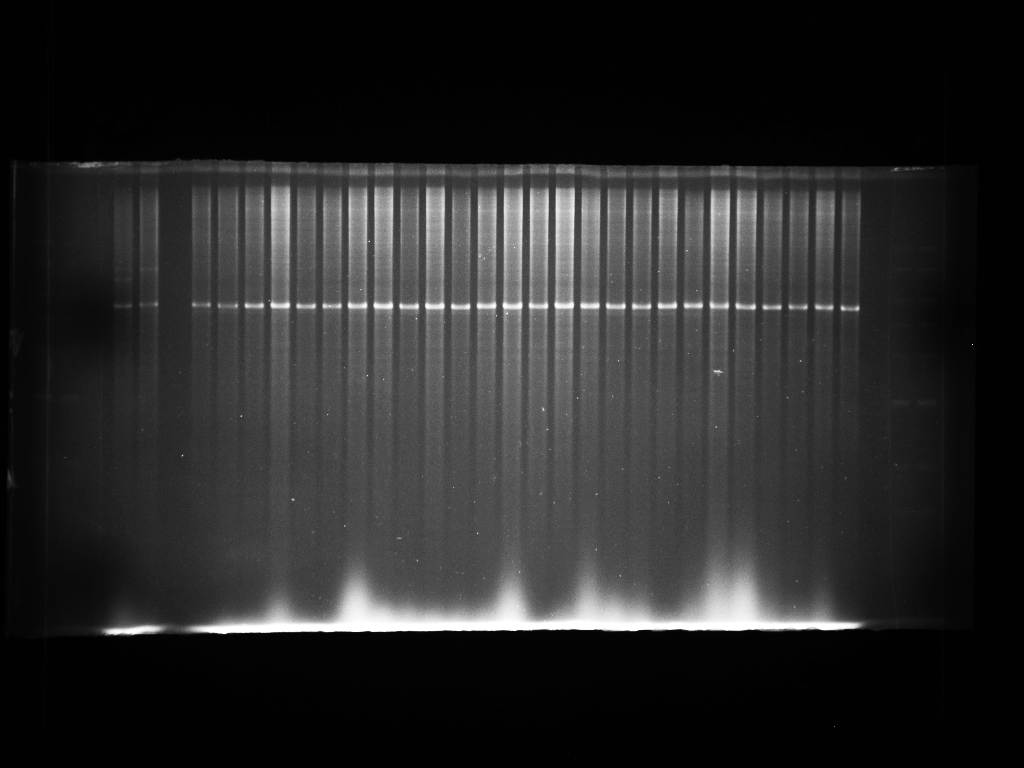

Supplement: Figure 6—source data 3. [file elife-91223-fig6-data3.zip › Figure 6A source data3.tif]

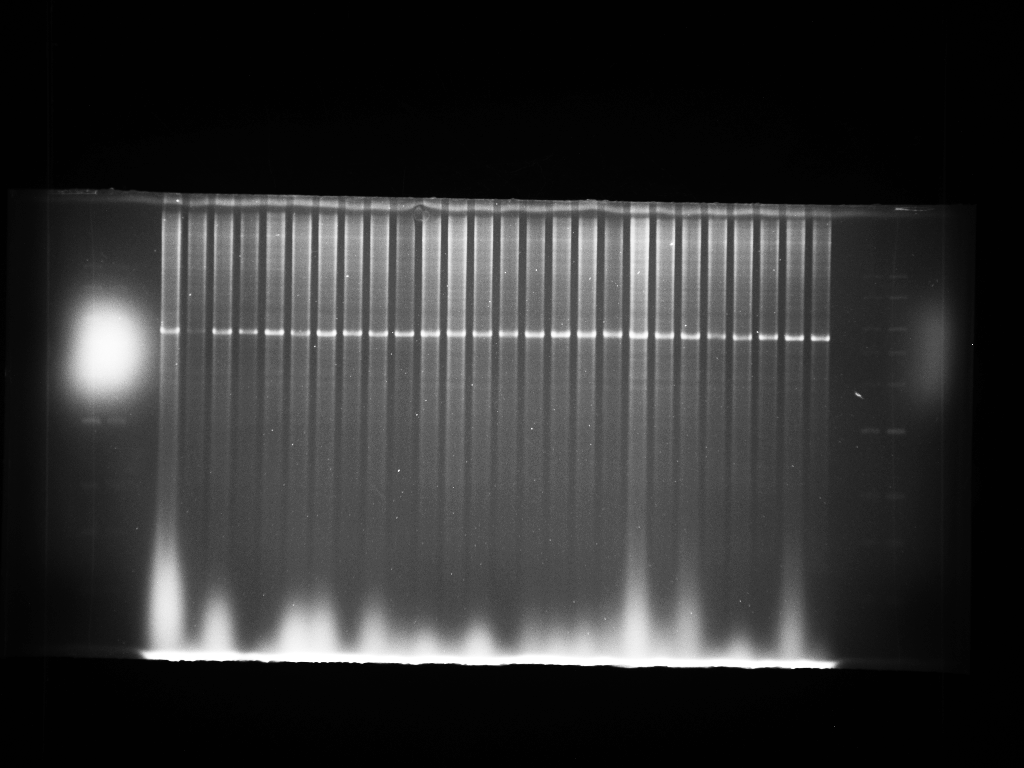

Supplement: Figure 6—source data 4. [file elife-91223-fig6-data4.zip › Figure 6A source data4.tif]

SY12<sup>XYΔ+Y</sup>

SY12<sup>XYΔ+Y</sup> *tlc1Δ*

1 2 3 4 5 6 7 8 9 10 11 12 13 14 15 16 17 18 19 20 21 22 23 24 25

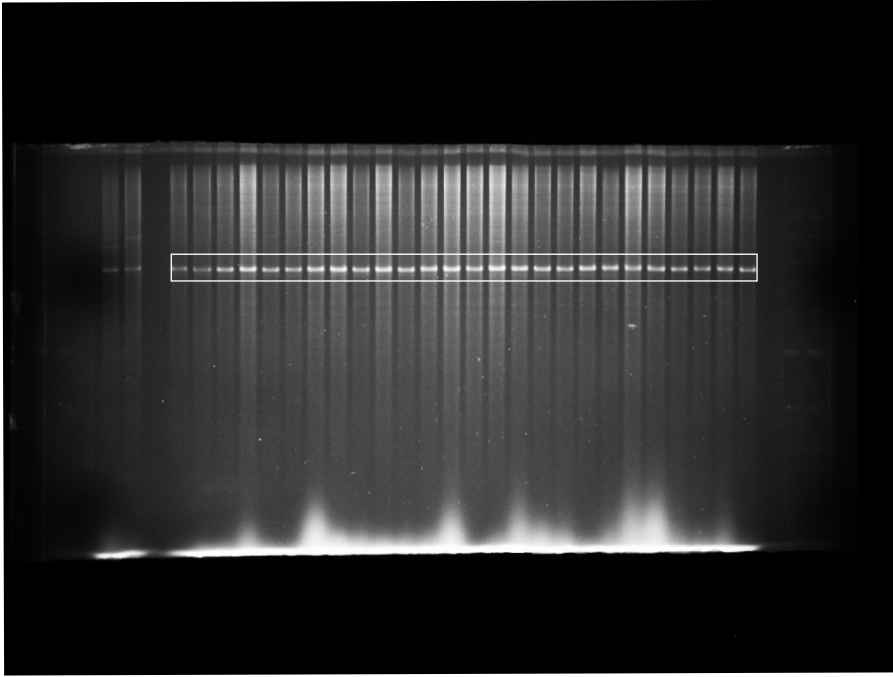

SY12<sup>XYΔ+Y</sup> *tlc1Δ*

26 27 28 29 30 31 32 33 34 35 36 37 38 39 40 41 42 43 44 45 46 47 48 49 50

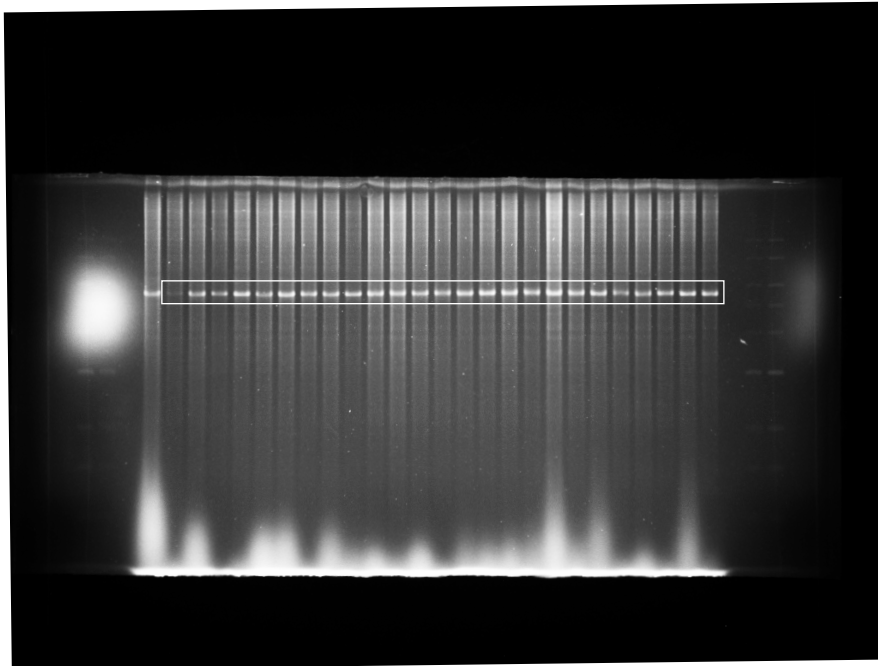

Supplement: Figure 6—source data 6. [file elife-91223-fig6-data6.zip › PDF containing original scans of the loading contral in Figure 6A.pdf]

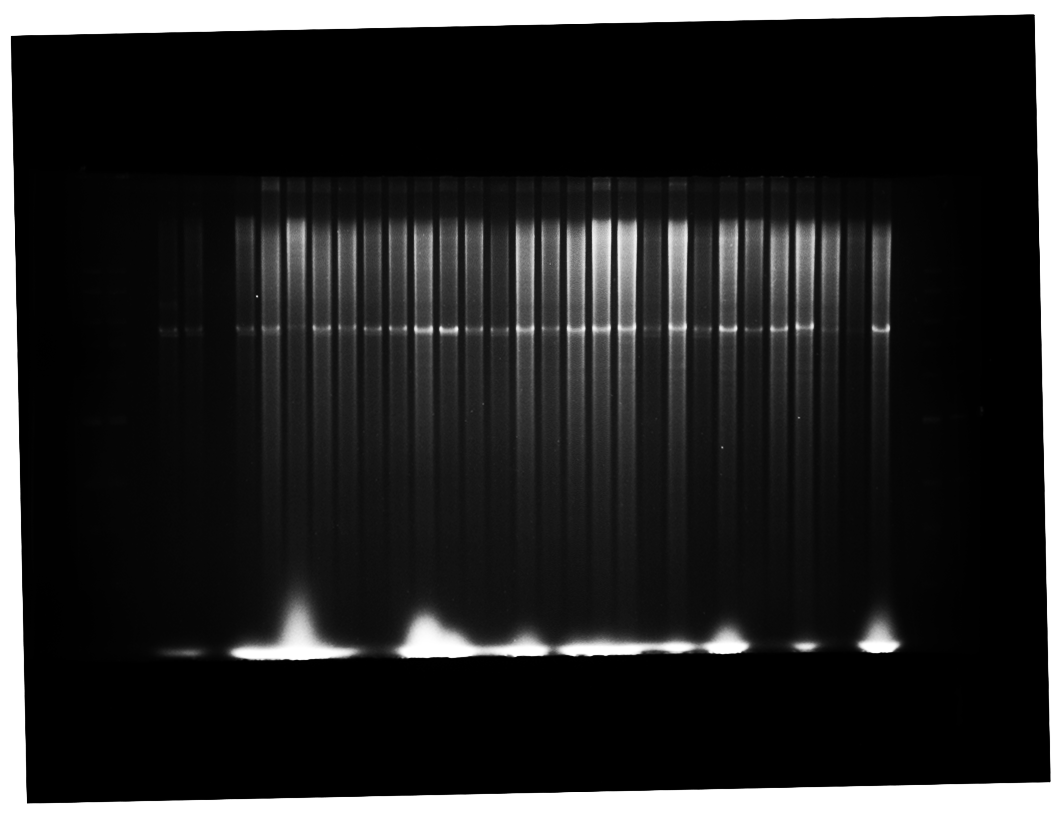

Supplement: Figure 6—source data 9. [file elife-91223-fig6-data9.zip › Figure 6B source data3.tif]

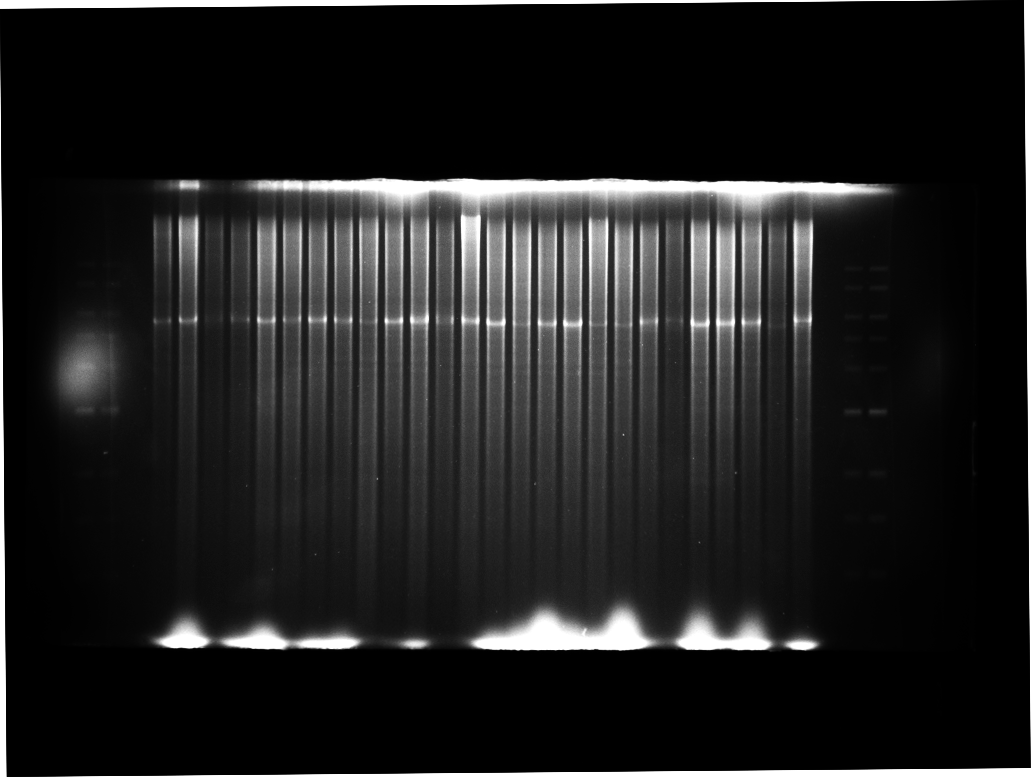

Supplement: Figure 6—source data 10. [file elife-91223-fig6-data10.zip › Figure 6B source data4.tif]

**B**

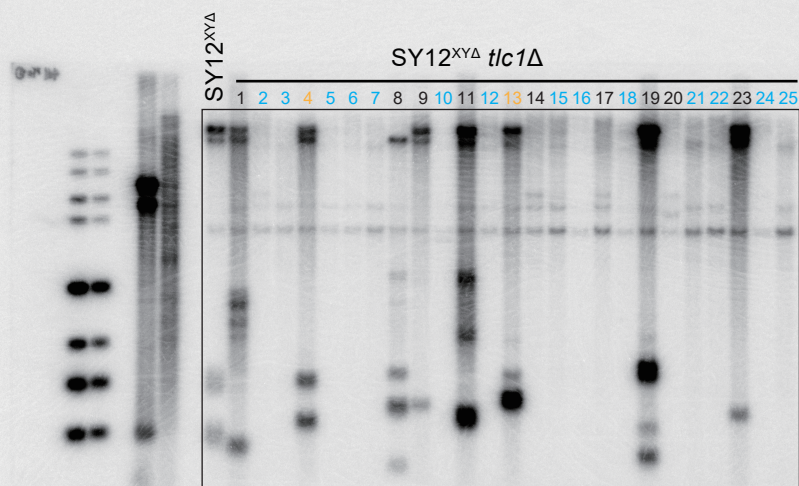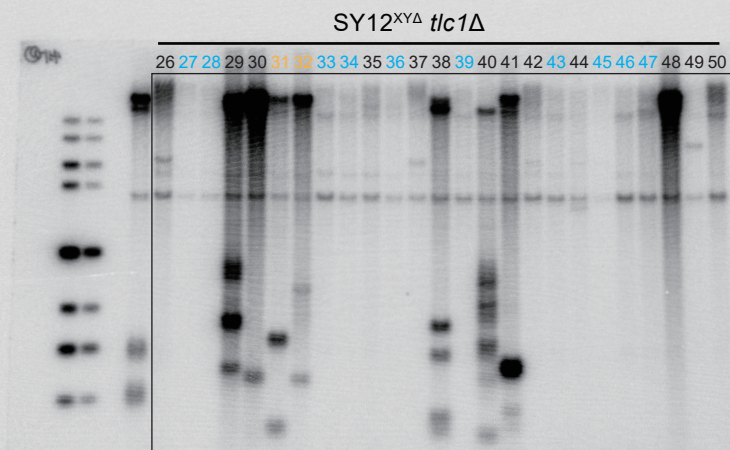

Supplement: Figure 6—source data 11. [file elife-91223-fig6-data11.zip › PDF containing Figure 6B and original scans of the relevant Southern blot analysis.pdf]

SY12<sup>XYΔ</sup>

SY12<sup>XYΔ</sup> *tlc1Δ*

1 2 3 4 5 6 7 8 9 10 11 12 13 14 15 16 17 18 19 20 21 22 23 24

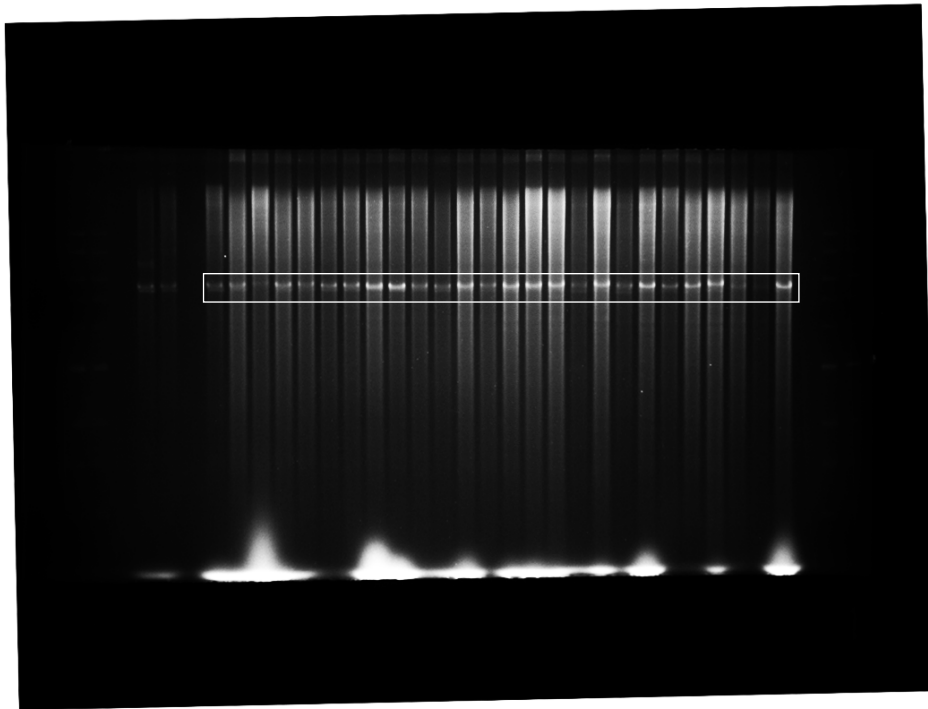

SY12<sup>XYΔ</sup> *tlc1Δ*

25 26 27 28 29 30 31 32 33 34 35 36 37 38 39 40 41 42 43 44 45 46 47 48 49 50

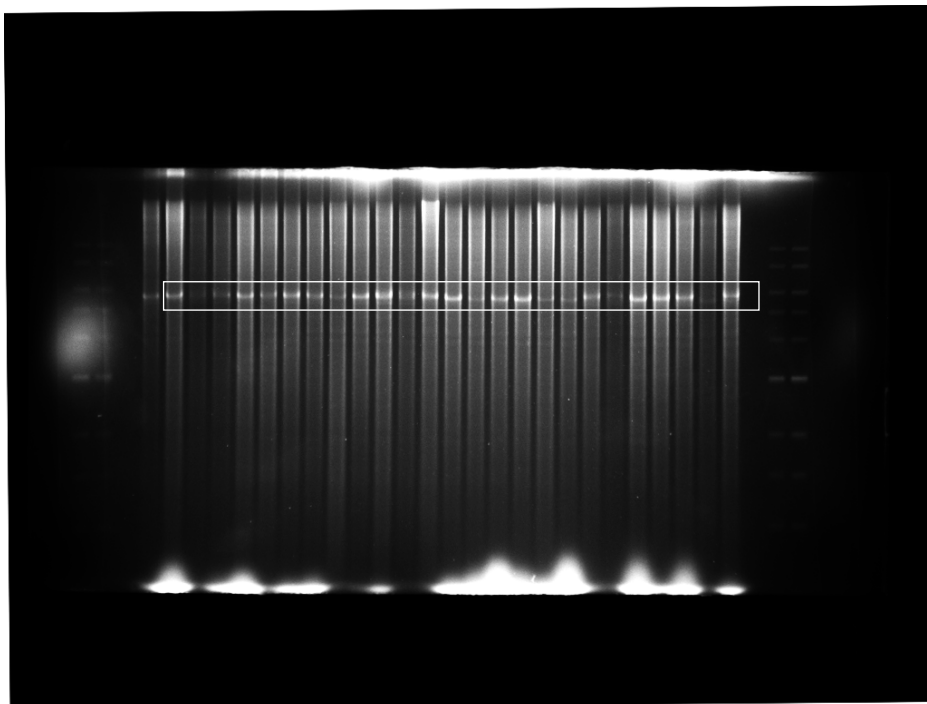

Supplement: Figure 6—source data 12. [file elife-91223-fig6-data12.zip › PDF containing original scans of the loading contral in Figure 6B.pdf]

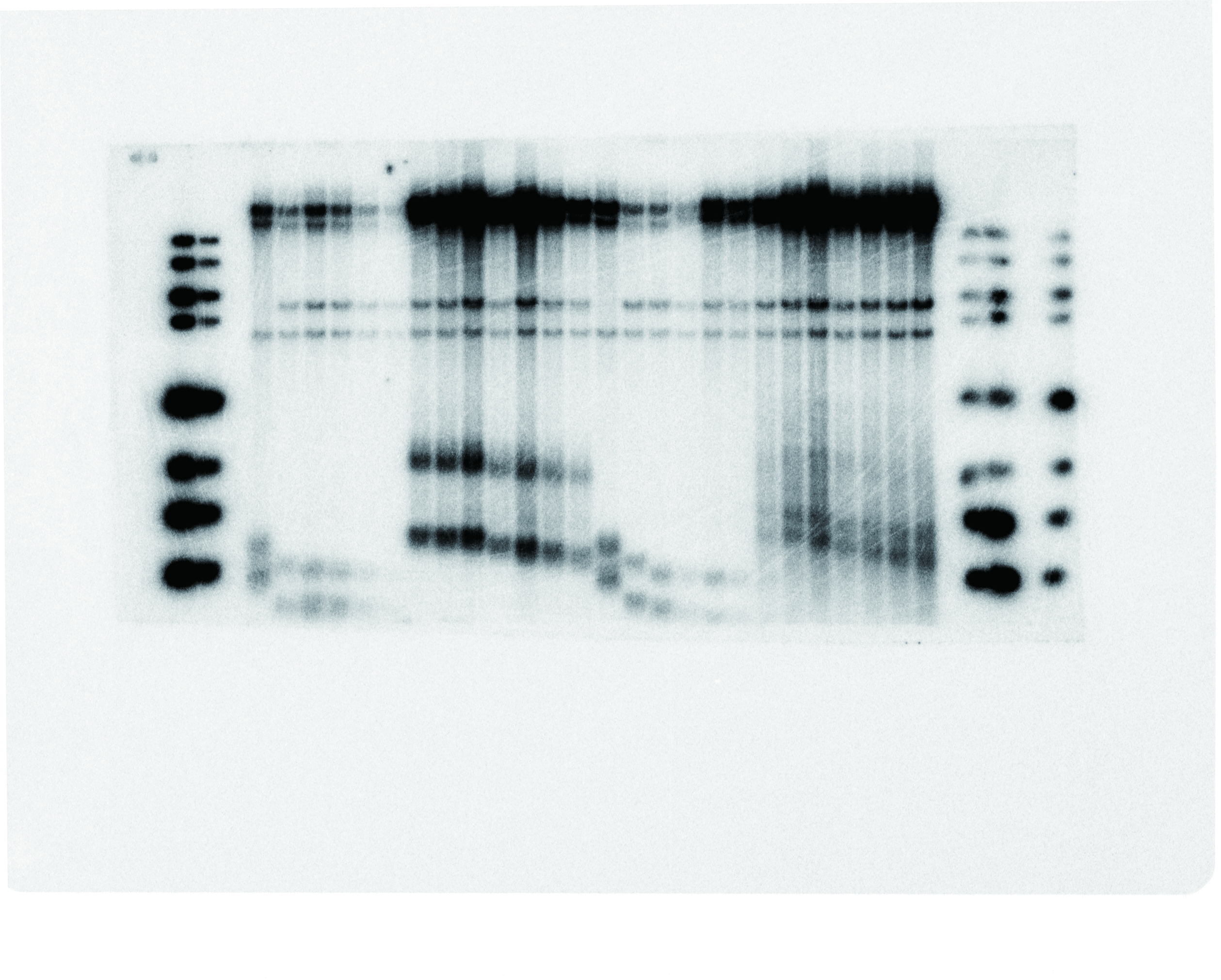

Supplement: Figure 6—figure supplement 1—source data 2. [file elife-91223-fig6-figsupp1-data2.zip › Figure 6-figure supplementary 1B- source data1.tif]

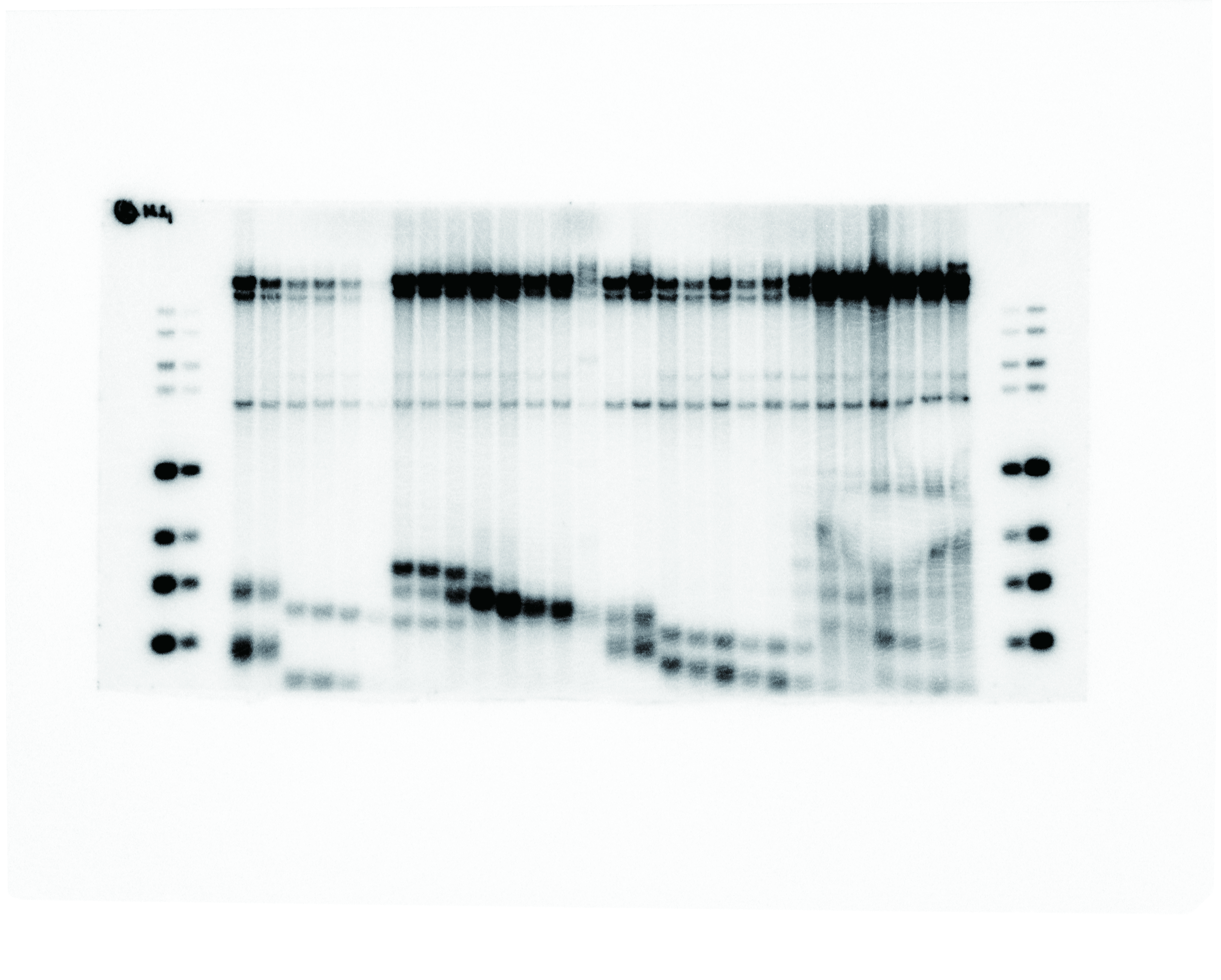

Supplement: Figure 6—figure supplement 1—source data 4. [file elife-91223-fig6-figsupp1-data4.zip › Figure 6-figure supplementary 1D- source data1.tif]

**B**

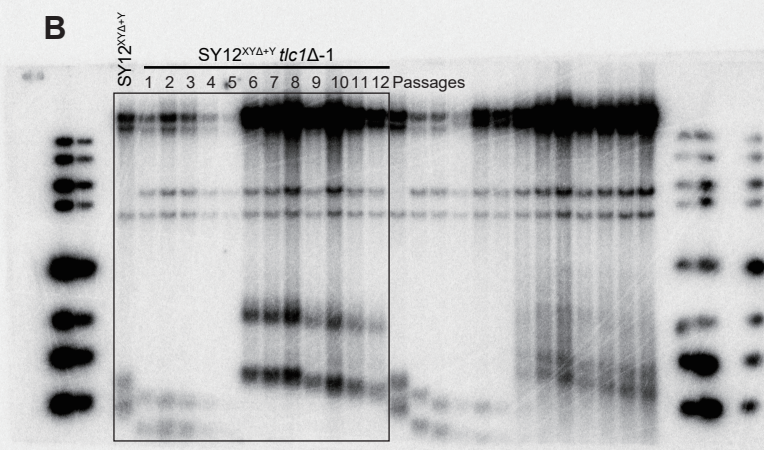

**D**

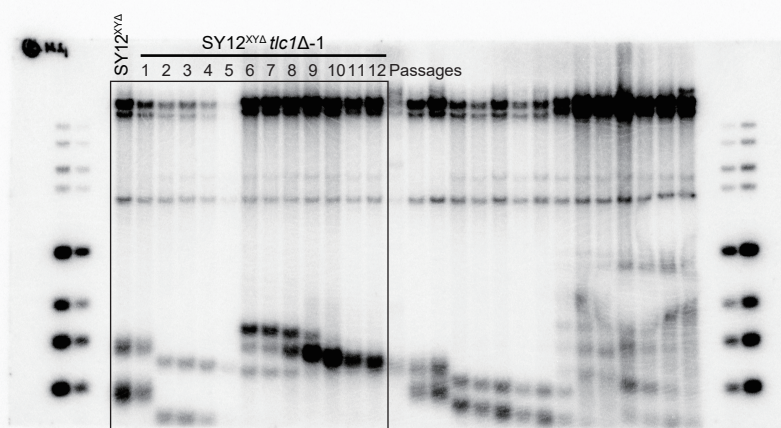

Supplement: Figure 6—figure supplement 1—source data 5. [file elife-91223-fig6-figsupp1-data5.zip › PDF containing Figure 6-figure supplementary 1 and original scans of the relevant Southern blot analysis.pdf]

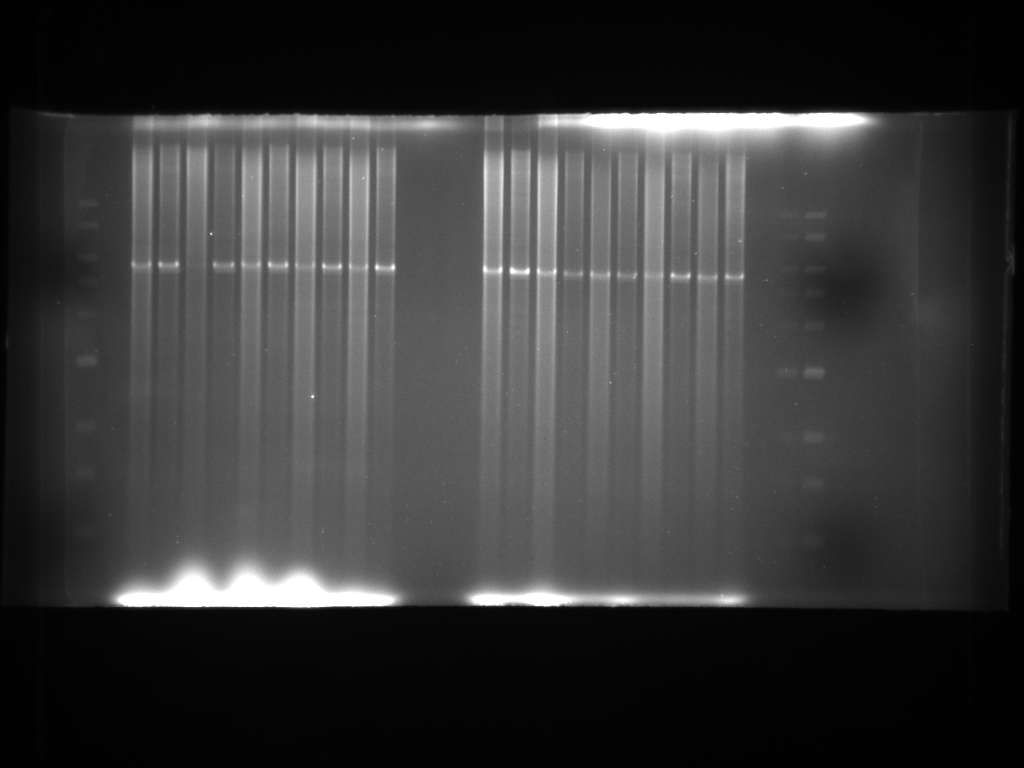

Supplement: Figure 6—figure supplement 3—source data 2. [file elife-91223-fig6-figsupp3-data2.zip › Figure 6-figure supplementary 3- source data 2.Tif]

0.5

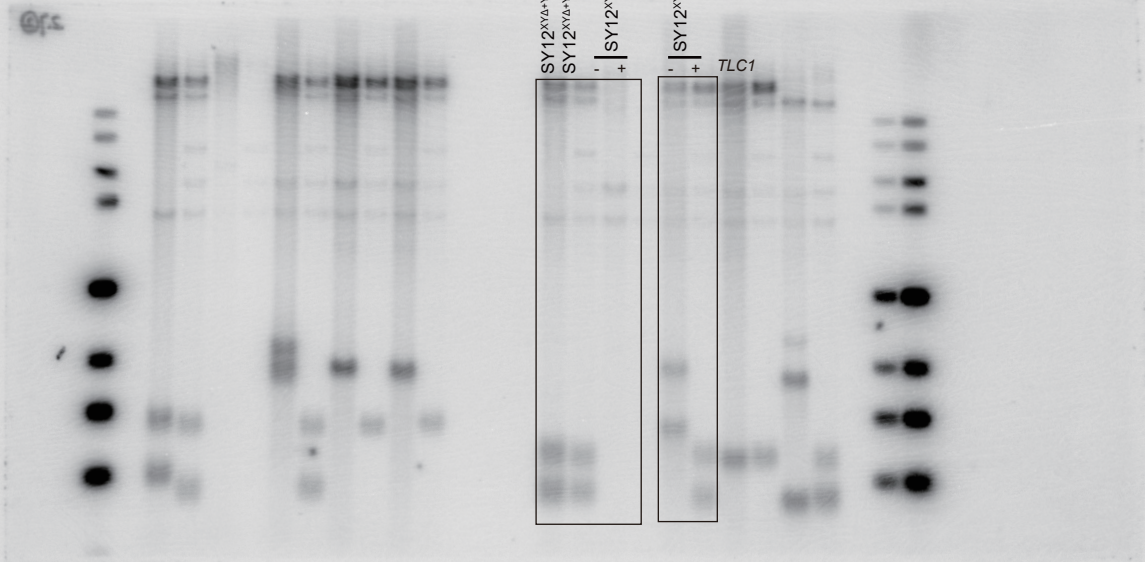

Supplement: Figure 6—figure supplement 3—source data 3. [file elife-91223-fig6-figsupp3-data3.zip › PDF containing Figure 6-figure supplementary 3 and original scans of the relevant Southern blot analysis.pdf]

SY12<sup>XYΔ-Y</sup>  
SY12<sup>XYΔ-Y</sup>-*flc1Δ* TLC1  
+ SY12<sup>XYΔ-Y</sup> *flc1Δ*-C1  
+ SY12<sup>XYΔ-Y</sup> *flc1Δ*-T1

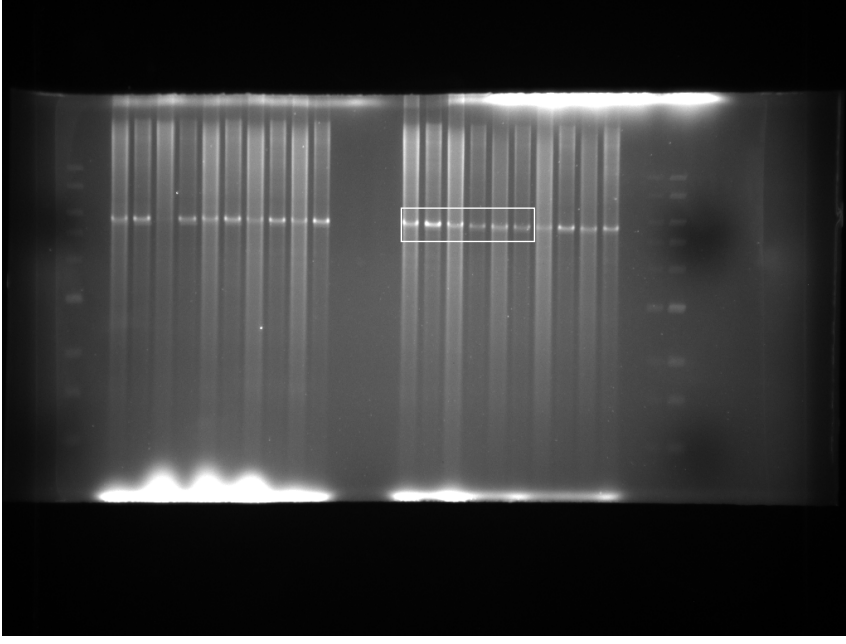

Supplement: Figure 6—figure supplement 3—source data 4. [file elife-91223-fig6-figsupp3-data4.zip › PDF containing original scans of the loading contral in Figure 6 figure supplementary 3.pdf]

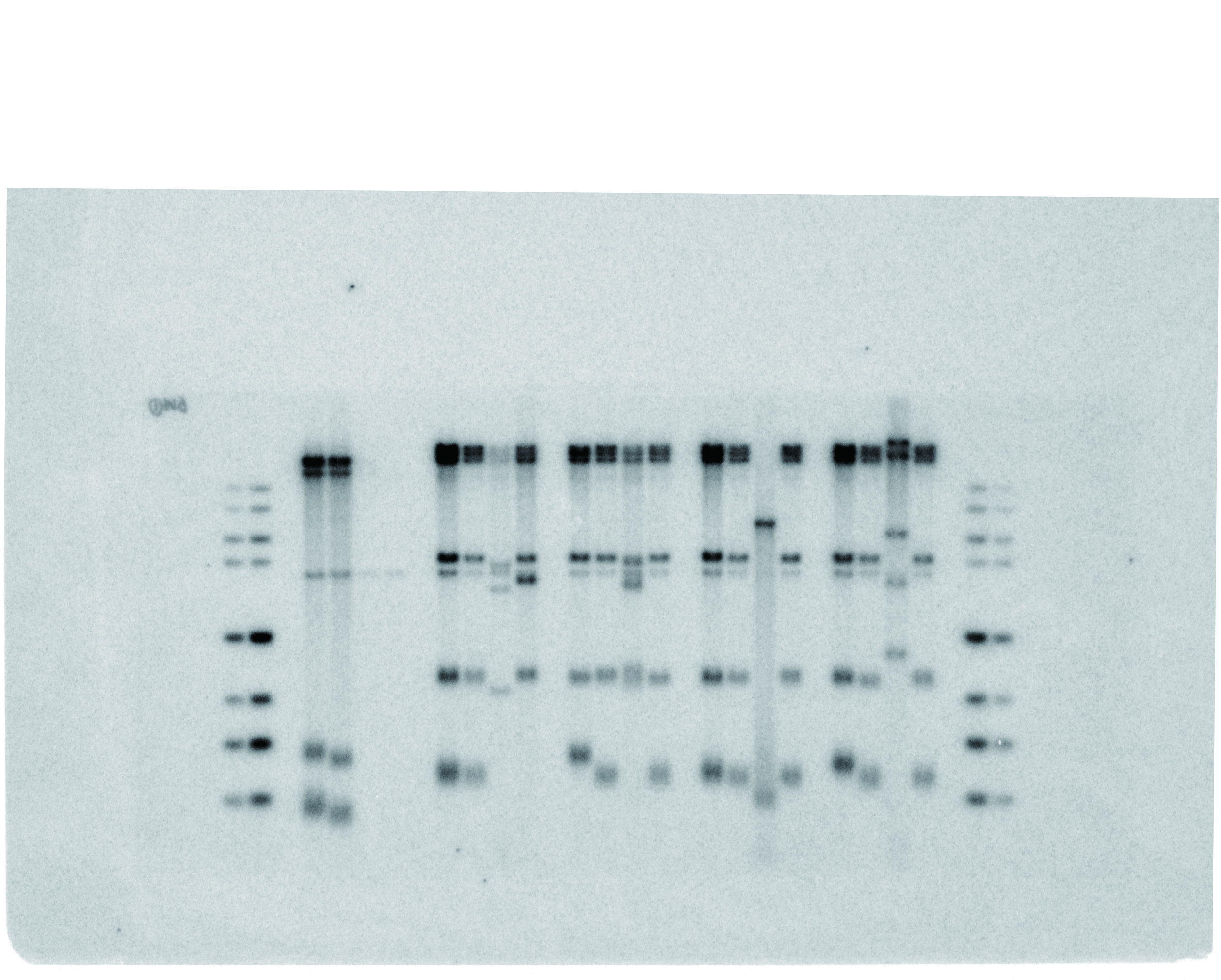

Supplement: Figure 6—figure supplement 5—source data 1. [file elife-91223-fig6-figsupp5-data1.zip › Figure 6-figure supplementary 5A- source data1.tif]

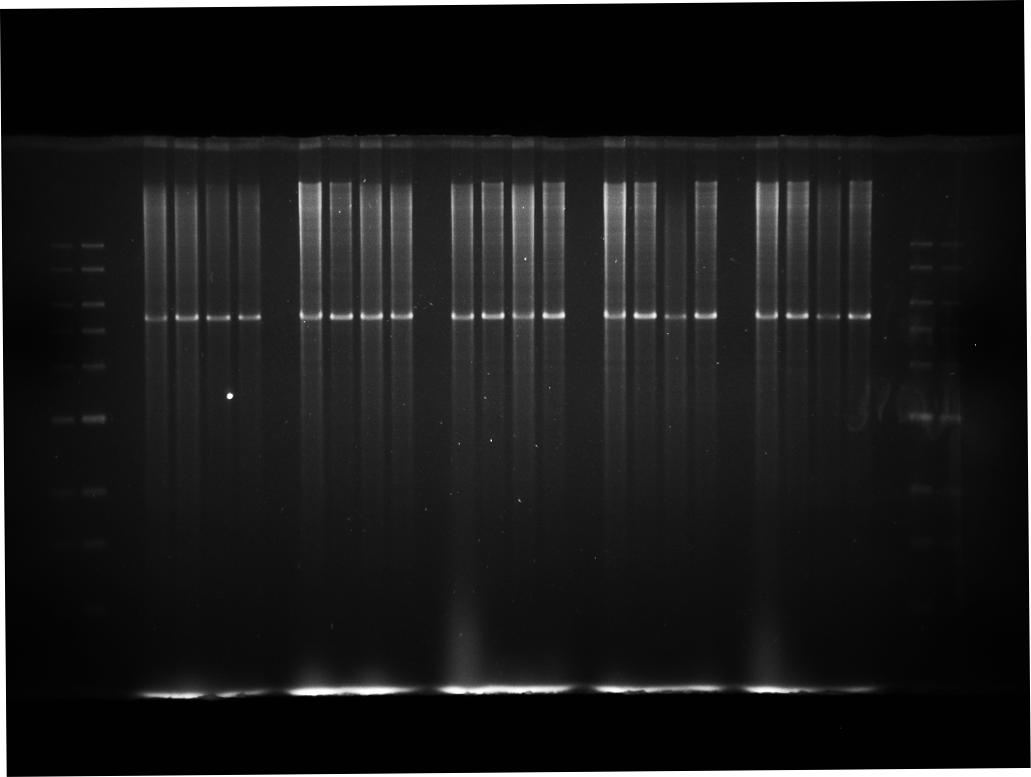

Supplement: Figure 6—figure supplement 5—source data 2. [file elife-91223-fig6-figsupp5-data2.zip › Figure 6-figure supplementary 5A- source data 2.tif]

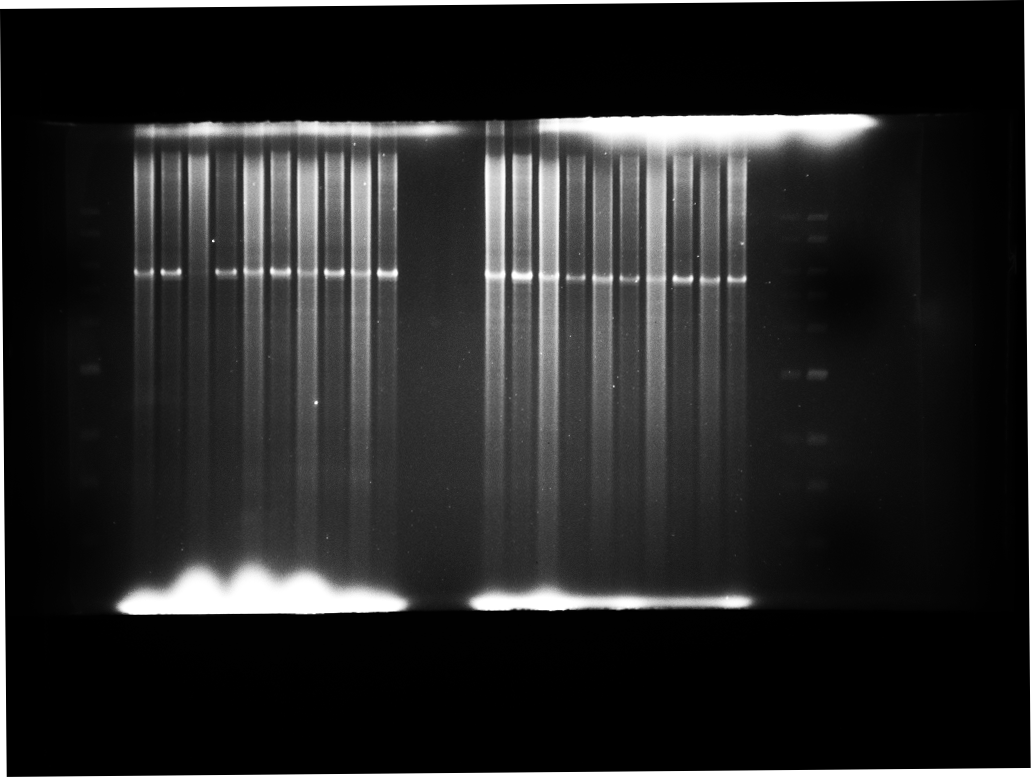

Supplement: Figure 6—figure supplement 5—source data 4. [file elife-91223-fig6-figsupp5-data4.zip › Figure 6-figure supplementary 5B- source data 2.tif]

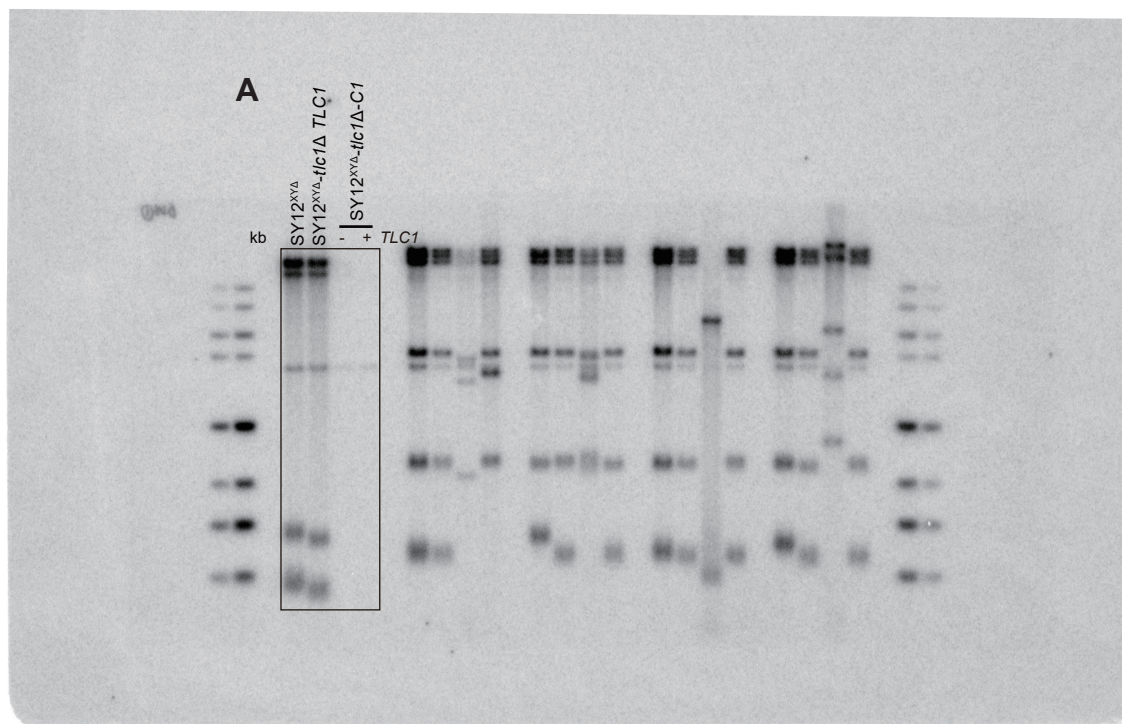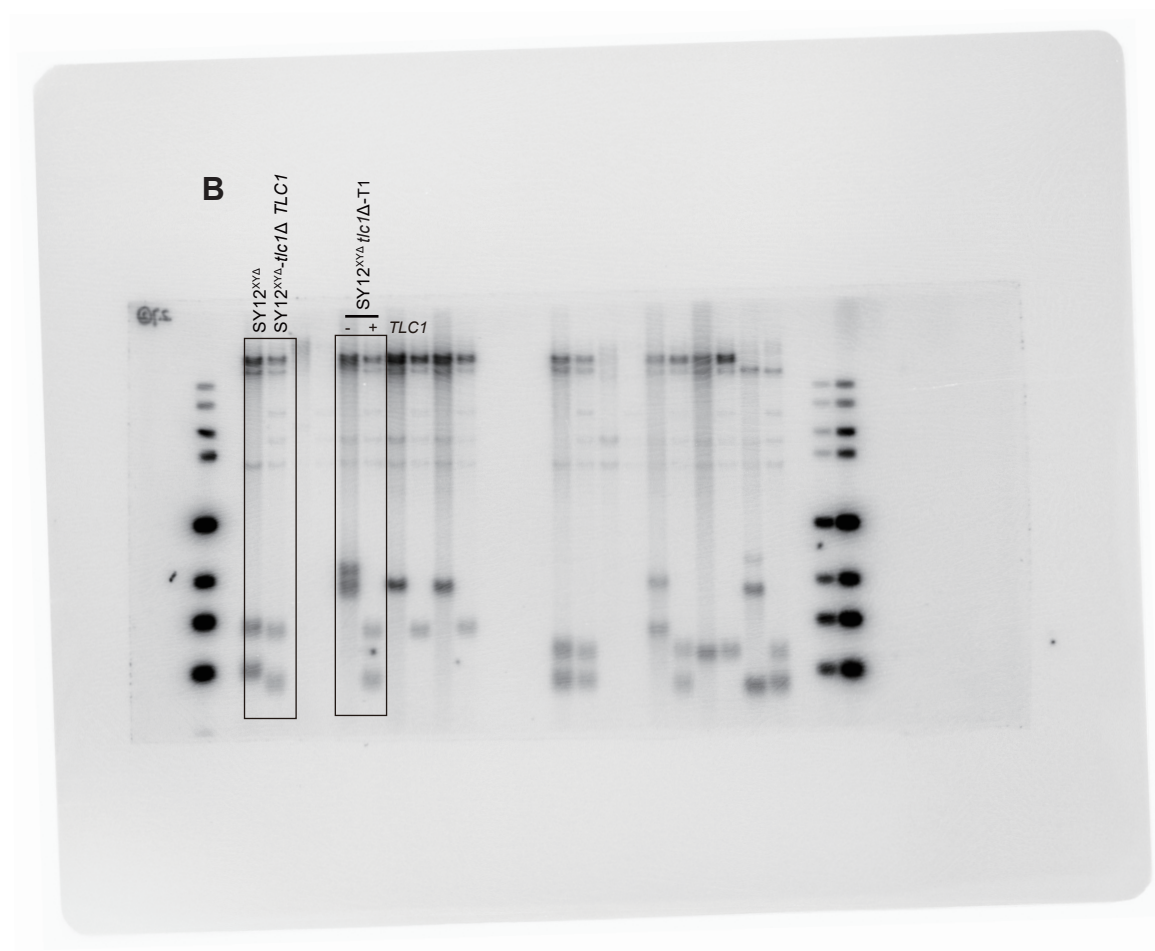

Supplement: Figure 6—figure supplement 5—source data 5. [file elife-91223-fig6-figsupp5-data5.zip › PDF containing Figure 6-figure supplementary 5 and original scans of the relevant Southern blot analysis.pdf]

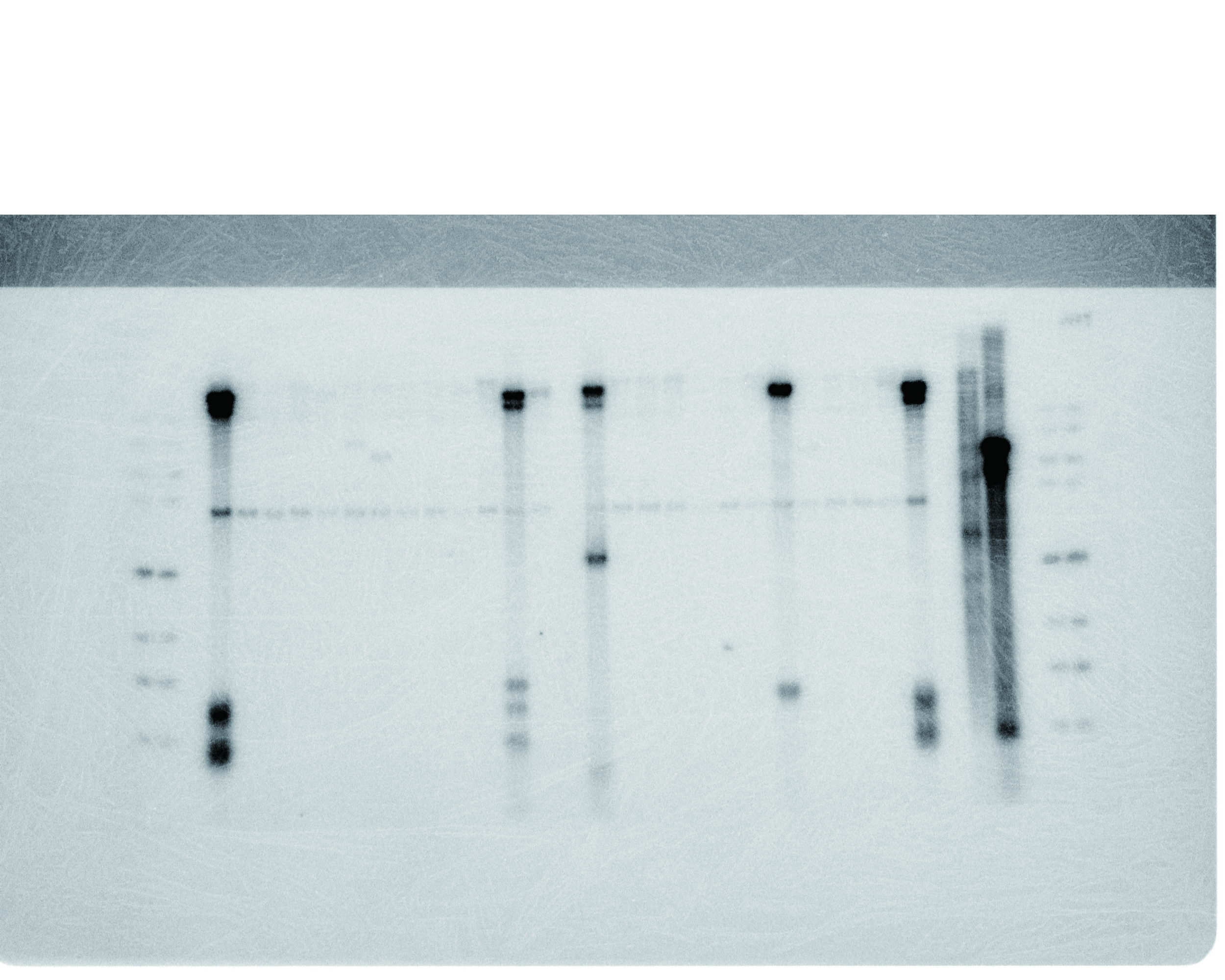

Supplement: Figure 6—figure supplement 6—source data 3. [file elife-91223-fig6-figsupp6-data3.zip › Figure 6-figure supplementary 6B- source data1.tif]

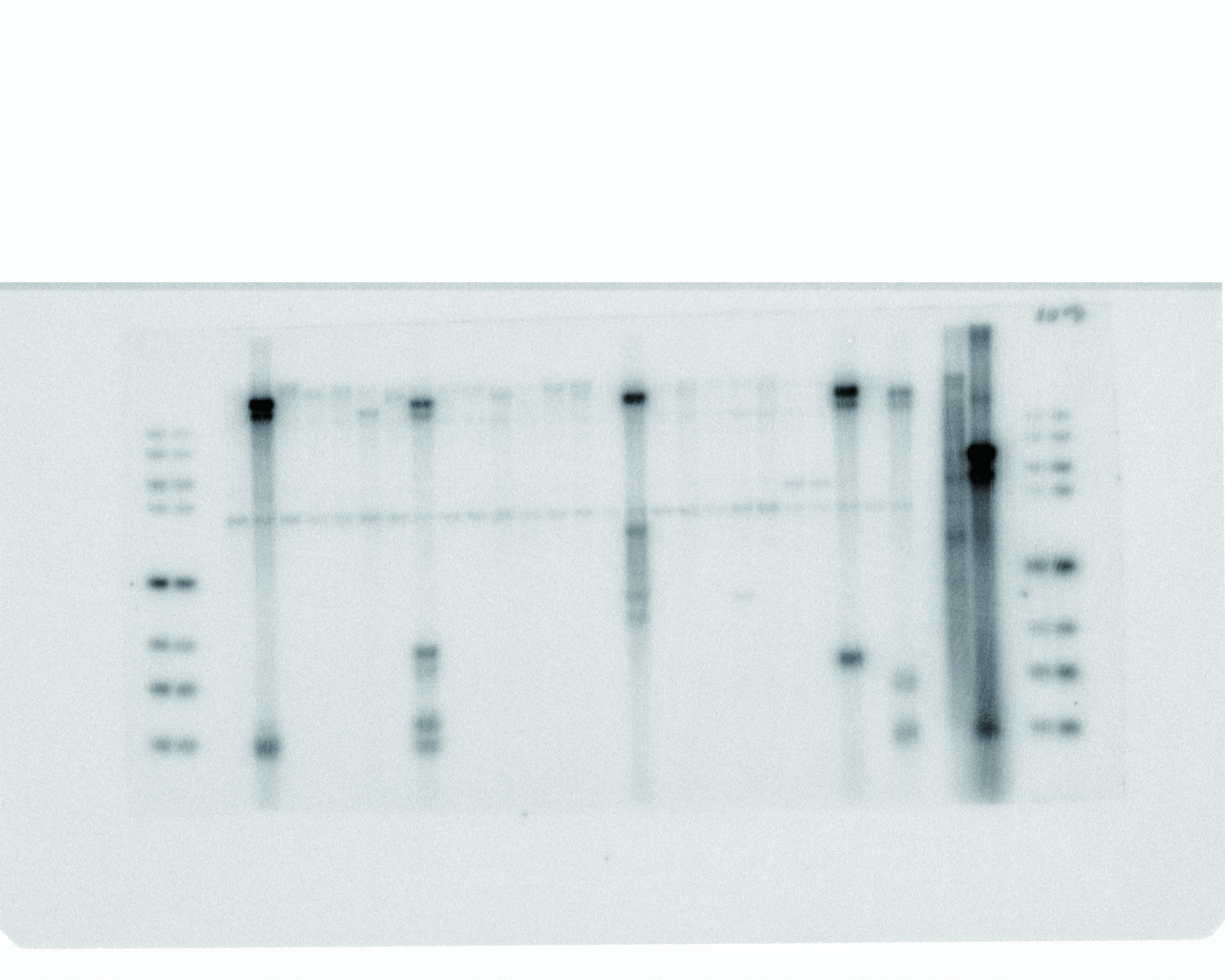

Supplement: Figure 6—figure supplement 6—source data 4. [file elife-91223-fig6-figsupp6-data4.zip › Figure 6-figure supplementary 6B- source data2.tif]

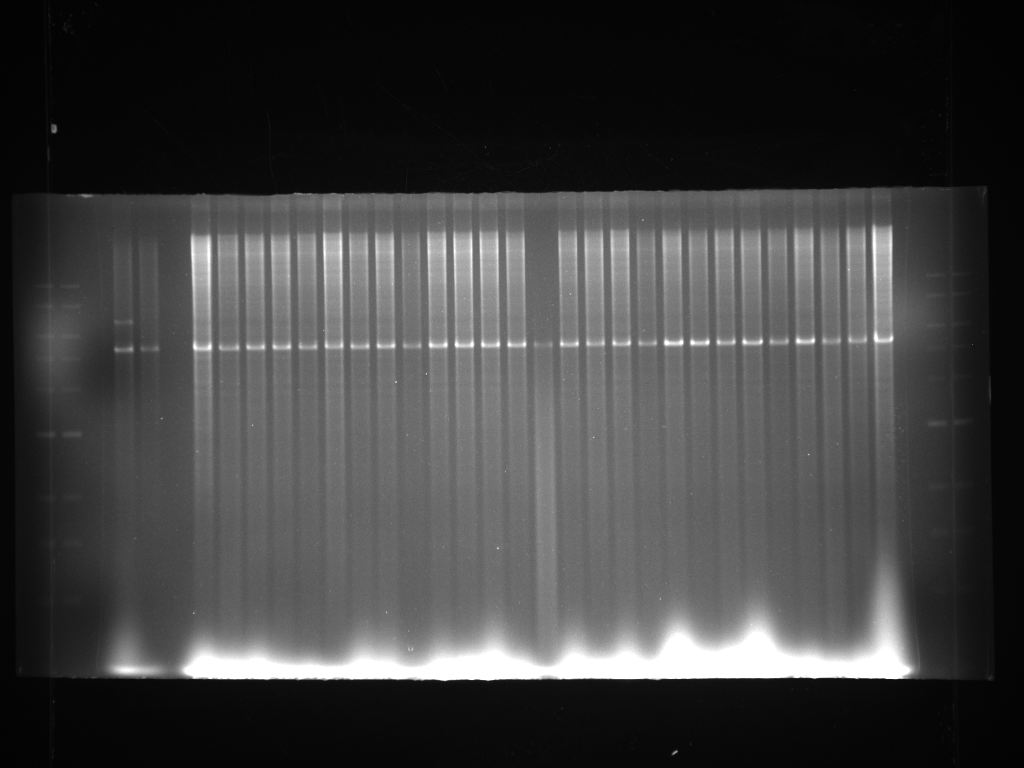

Supplement: Figure 6—figure supplement 6—source data 5. [file elife-91223-fig6-figsupp6-data5.zip › Figure 6-figure supplementary 6B- source data3.Tif]

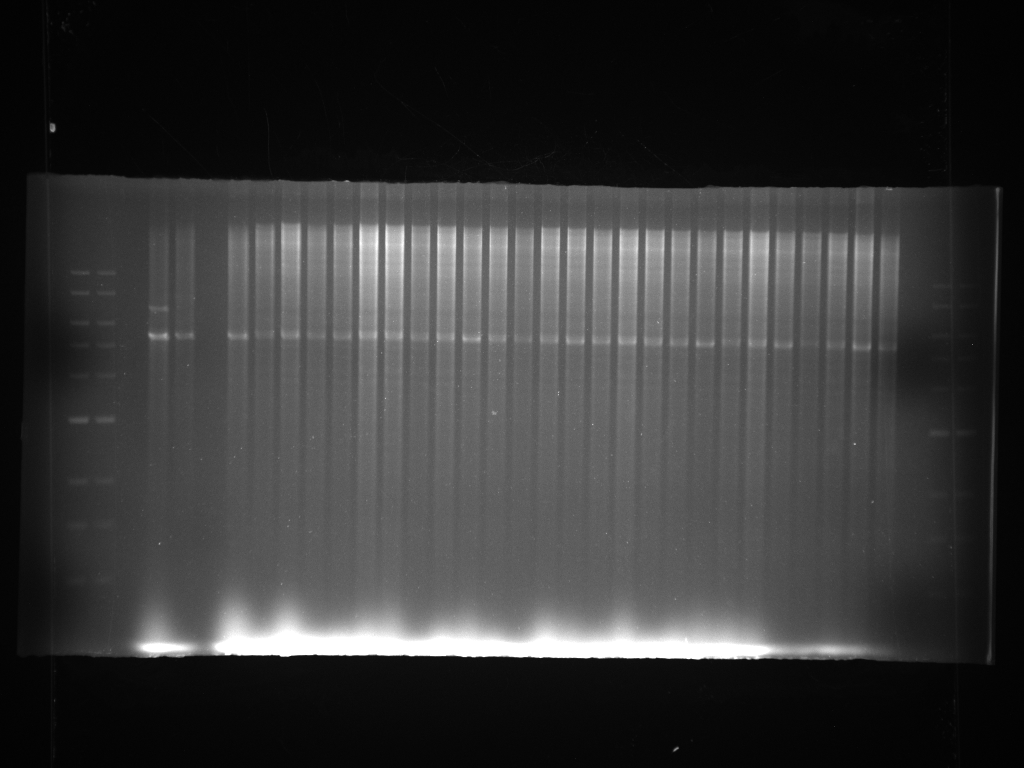

Supplement: Figure 6—figure supplement 6—source data 6. [file elife-91223-fig6-figsupp6-data6.zip › Figure 6-figure supplementary 6B- source data4.Tif]

**B**

SY12<sup>XYΔ+Y</sup>

SY12<sup>XYΔ+Y</sup> *tlc1Δ rad52Δ*

1 2 3 4 5 6 7 8 9 10 11 12 13 14 15 16 17 18 19 20 21 22 23 24 25

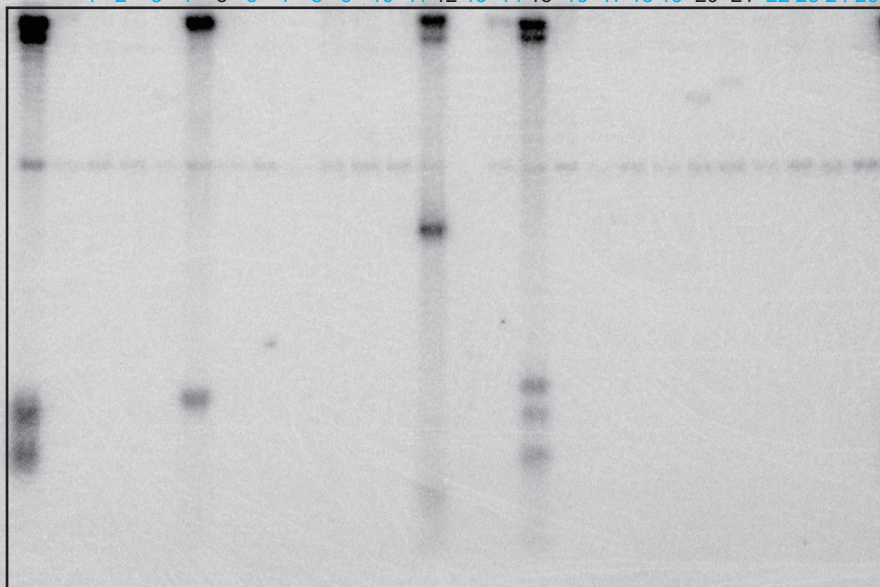

SY12<sup>XYΔ</sup>

SY12<sup>XYΔ</sup> *tlc1Δ rad52Δ*

1 2 3 4 5 6 7 8 9 10 11 12 13 14 15 16 17 18 19 20 21 22 23 24 25

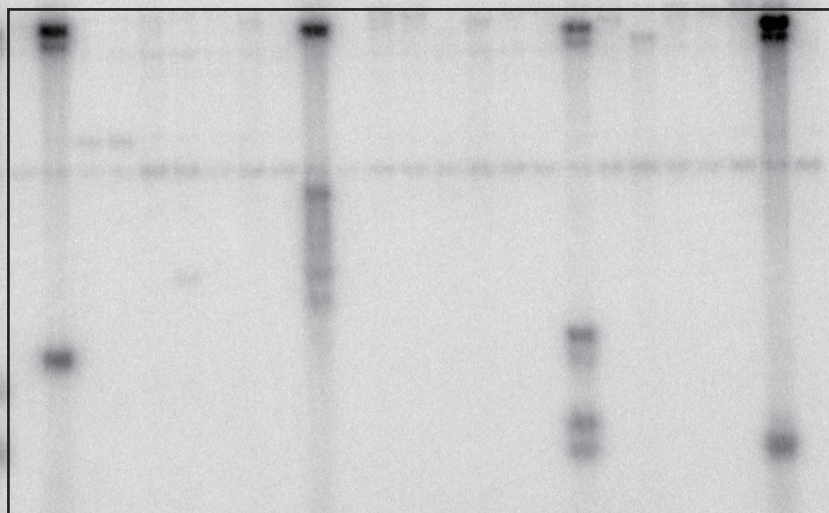

Supplement: Figure 6—figure supplement 6—source data 7. [file elife-91223-fig6-figsupp6-data7.zip › PDF containing Figure 6-figure supplementary 6 and original scans of the relevant Southern blot analysis.pdf]

**B**

SY12<sup>XYΔ+Y</sup>

SY12<sup>XYΔ+Y</sup> *tlc1Δ rad52Δ*

1 2 3 4 5 6 7 8 9 10 11 12 13 14 15 16 17 18 19 20 21 22 23 24 25

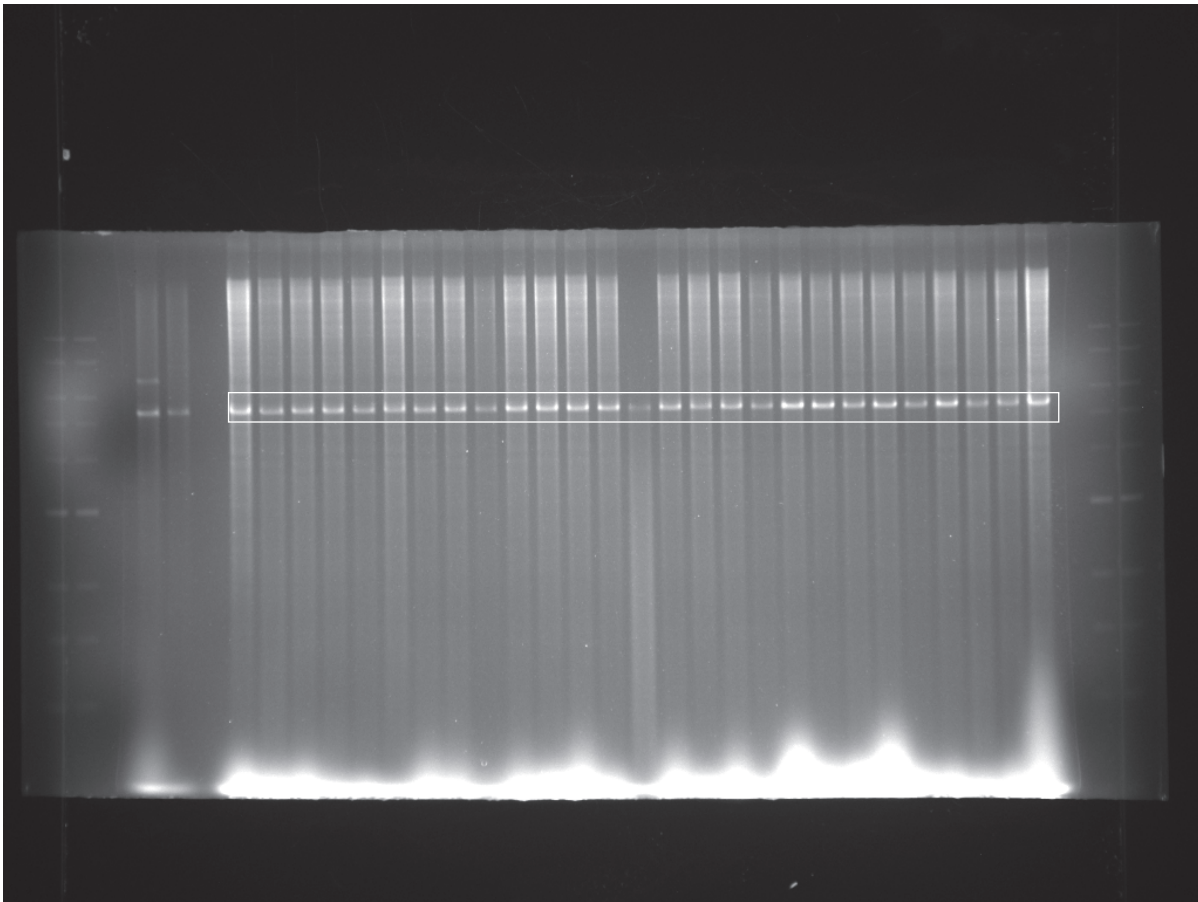

SY12<sup>XYΔ</sup>

SY12<sup>XYΔ</sup> *tlc1Δ rad52Δ*

1 2 3 4 5 6 7 8 9 10 11 12 13 14 15 16 17 18 19 20 21 22 23 24 25

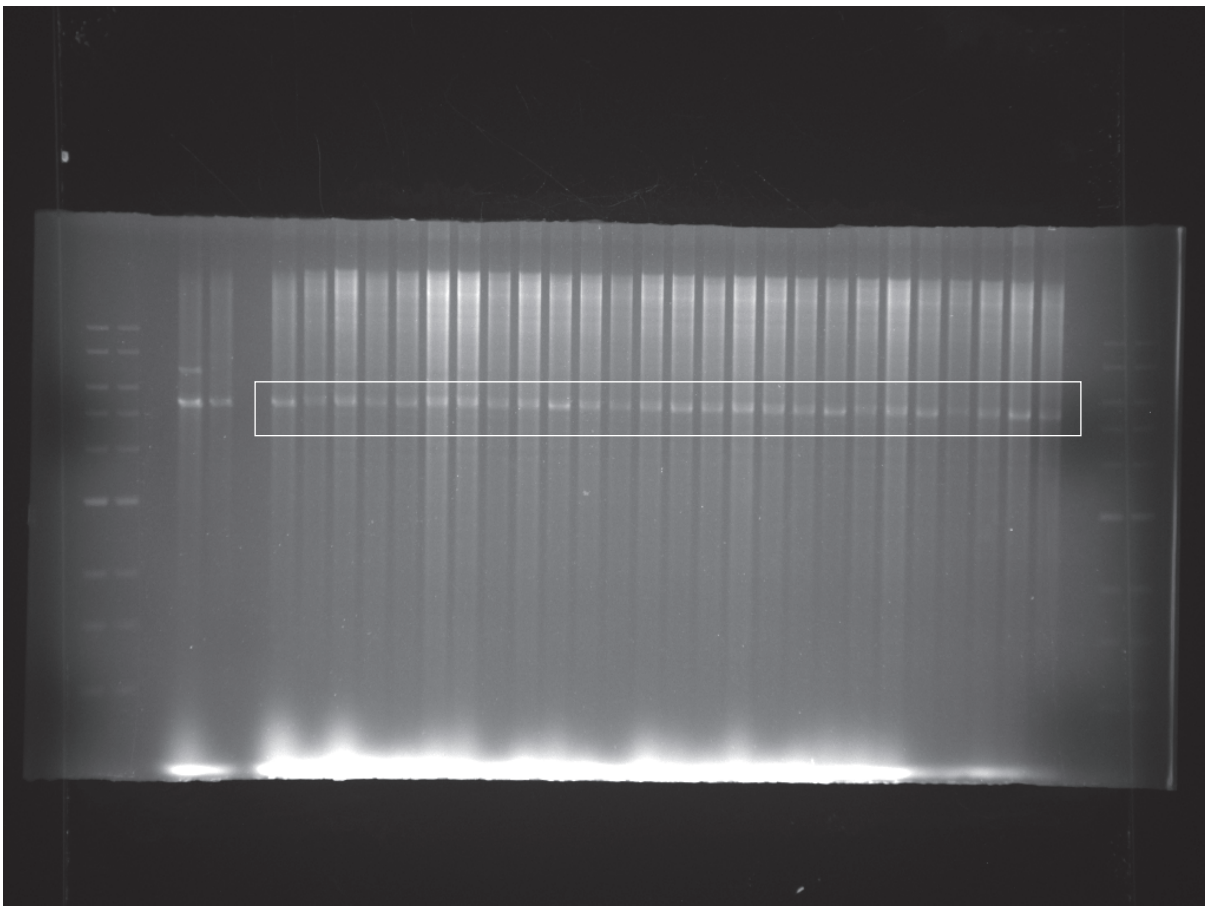

Supplement: Figure 6—figure supplement 6—source data 8. [file elife-91223-fig6-figsupp6-data8.zip › PDF containing original scans of the loading contral in Figure 6 figure supplementary 6B.pdf]

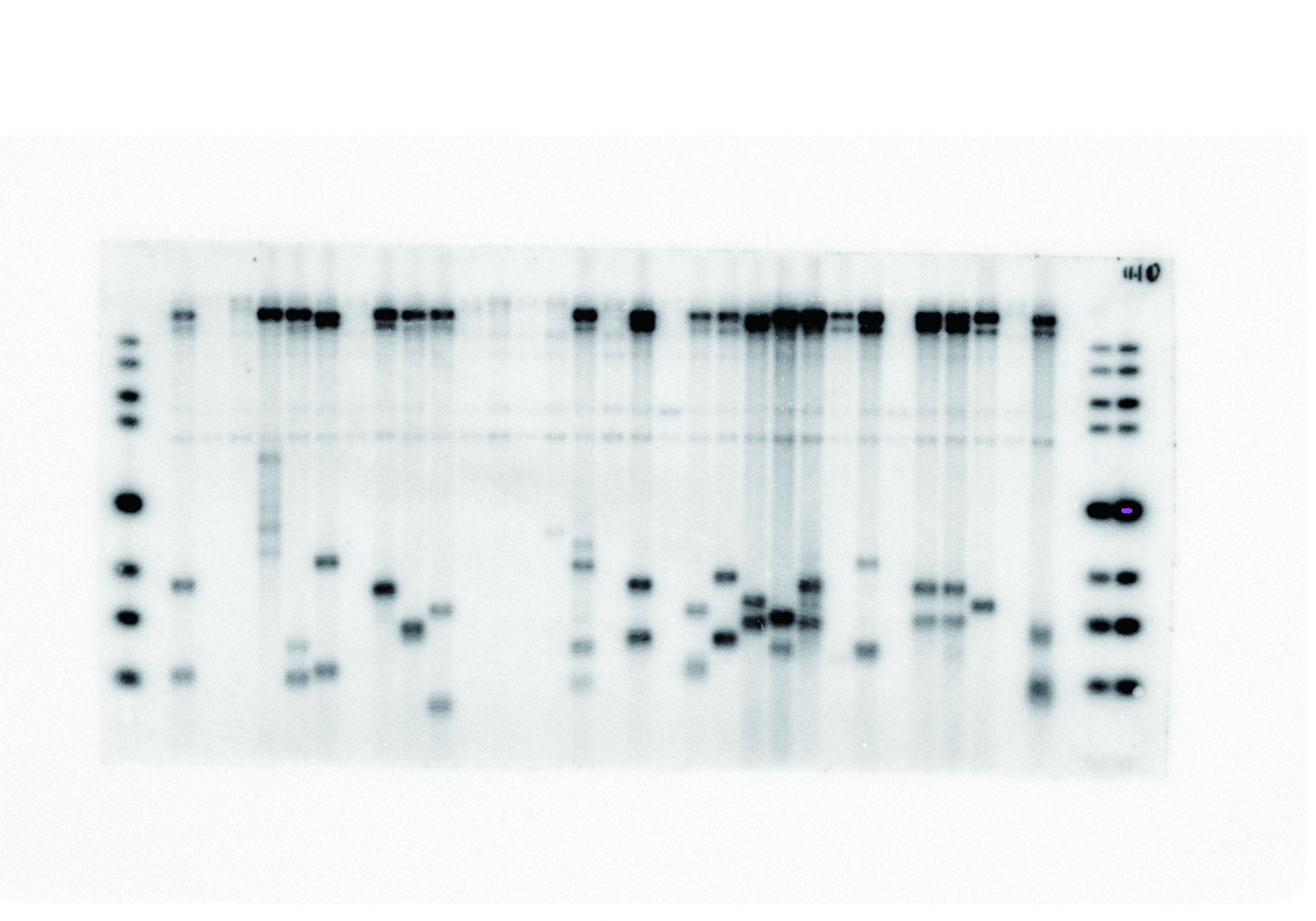

Supplement: Figure 6—figure supplement 7—source data 1. [file elife-91223-fig6-figsupp7-data1.zip › Figure 6-figure supplementary 7A- source data1.tif]

**A**

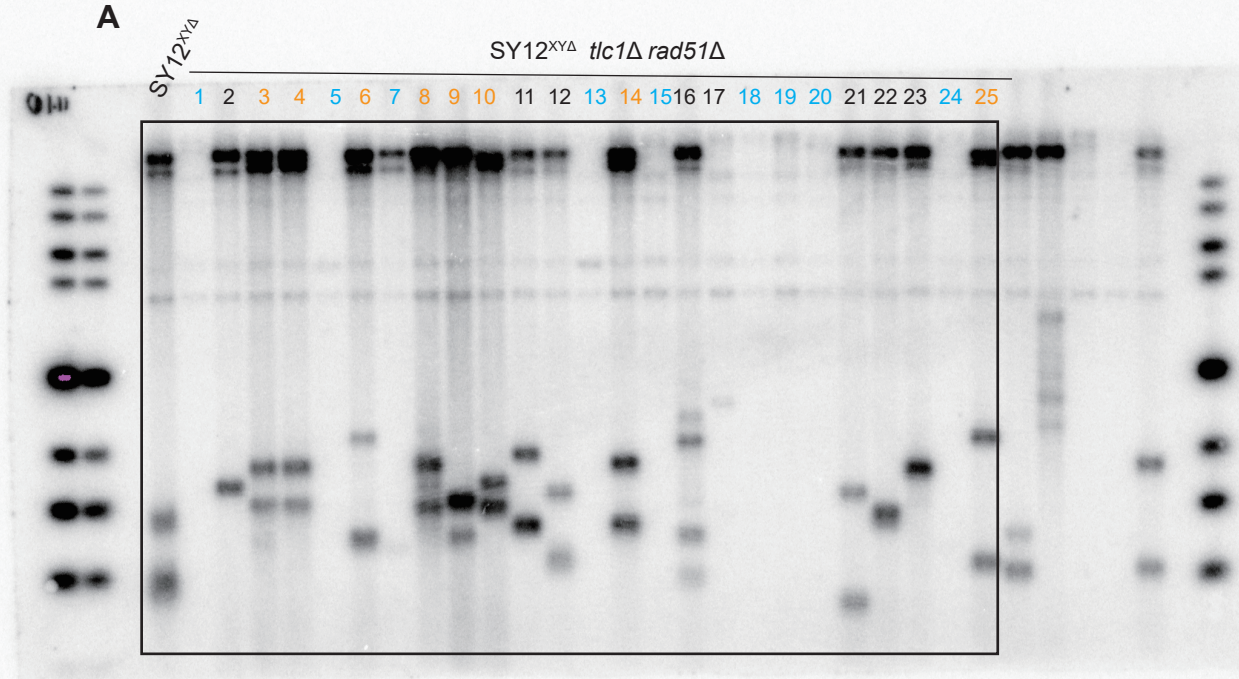

Supplement: Figure 6—figure supplement 7—source data 2. [file elife-91223-fig6-figsupp7-data2.zip › PDF containing Figure 6-figure supplementary 7 and original scans of the relevant Southern blot analysis.pdf]
