# Supplementary material for: Elimination of subtelomeric repeat sequences exerts little effect on telomere essential functions in Saccharomyces cerevisiae
Source: eLife. 2024 Apr 24;12:RP91223. doi: 10.7554/eLife.91223 (PMC11042809; doi:10.7554/eLife.91223)
Supplement: Figure 2—figure supplement 5—source data 2. [file elife-91223-fig2-figsupp5-data2.zip › PDF containing Figure 2-figure supplementary5 and original scans of the relevant Southern blot analysis.pdf]

A

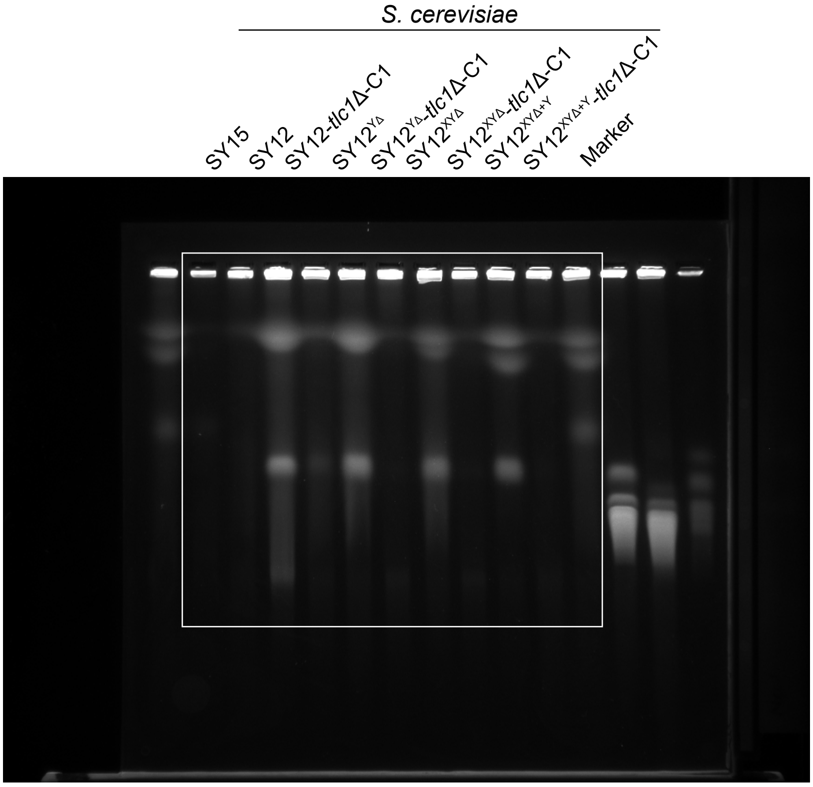
S. cerevisiae
SY15
SY12
SY12-tlc1Δ-C1
SY12YΔ
SY12YΔ-tlc1Δ-C1
SY12XYΔ
SY12XYΔ-tlc1Δ-C1
SY12XYΔ+Y
SY12XYΔ+Y-tlc1Δ-C1
Marker
