# Supplementary material for: Elimination of subtelomeric repeat sequences exerts little effect on telomere essential functions in Saccharomyces cerevisiae
Source: eLife. 2024 Apr 24;12:RP91223. doi: 10.7554/eLife.91223 (PMC11042809; doi:10.7554/eLife.91223)
Supplement: Figure 3—source data 20. [file elife-91223-fig3-data20.zip › Figure 3F-source data 1 Original FACS analysis results..pdf]

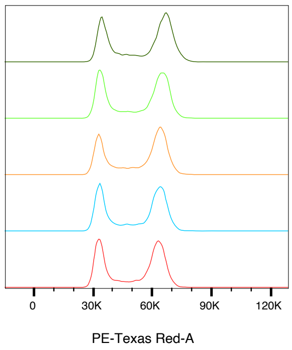

	Sample Name	Subset Name	Count
□	20220720_Tube_002_002.fcs	002-2	8553
□	20220720_Tube_005_005.fcs	002-2	8511
□	20220720_Tube_007_007.fcs	002-2	7828
□	20220720_Tube_025_025.fcs	002-2	7899
□	20220720_Tube_027_027.fcs	002-2	8064
