# Supplementary material for: Elimination of subtelomeric repeat sequences exerts little effect on telomere essential functions in Saccharomyces cerevisiae
Source: eLife. 2024 Apr 24;12:RP91223. doi: 10.7554/eLife.91223 (PMC11042809; doi:10.7554/eLife.91223)
Supplement: Figure 3—source data 27. [file elife-91223-fig3-data27.zip › PDF containing Figure 3G and original scans of the relevant dotting blot analysis.pdf]

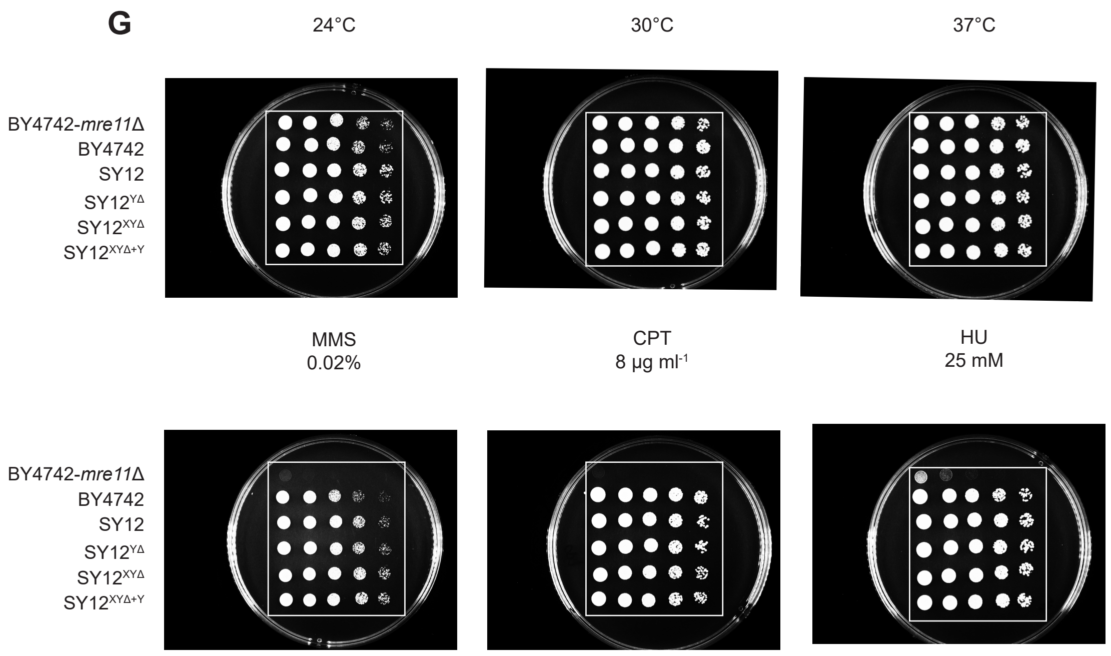
G
24°C
30°C
37°C
BY4742-mre11Δ
BY4742
SY12
SY12YΔ
SY12XYΔ
SY12XYΔ+Y
MMS
0.02%
CPT
8 µg ml-1
HU
25 mM
BY4742-mre11Δ
BY4742
SY12
SY12YΔ
SY12XYΔ
SY12XYΔ+Y
