# Supplementary material for: Elimination of subtelomeric repeat sequences exerts little effect on telomere essential functions in Saccharomyces cerevisiae
Source: eLife. 2024 Apr 24;12:RP91223. doi: 10.7554/eLife.91223 (PMC11042809; doi:10.7554/eLife.91223)
Supplement: Figure 5—figure supplement 1—source data 5. [file elife-91223-fig5-figsupp1-data5.zip › PDF containing Figure 5-figure supplementary1 and original scans of the relevant Southern blot analysis.pdf]

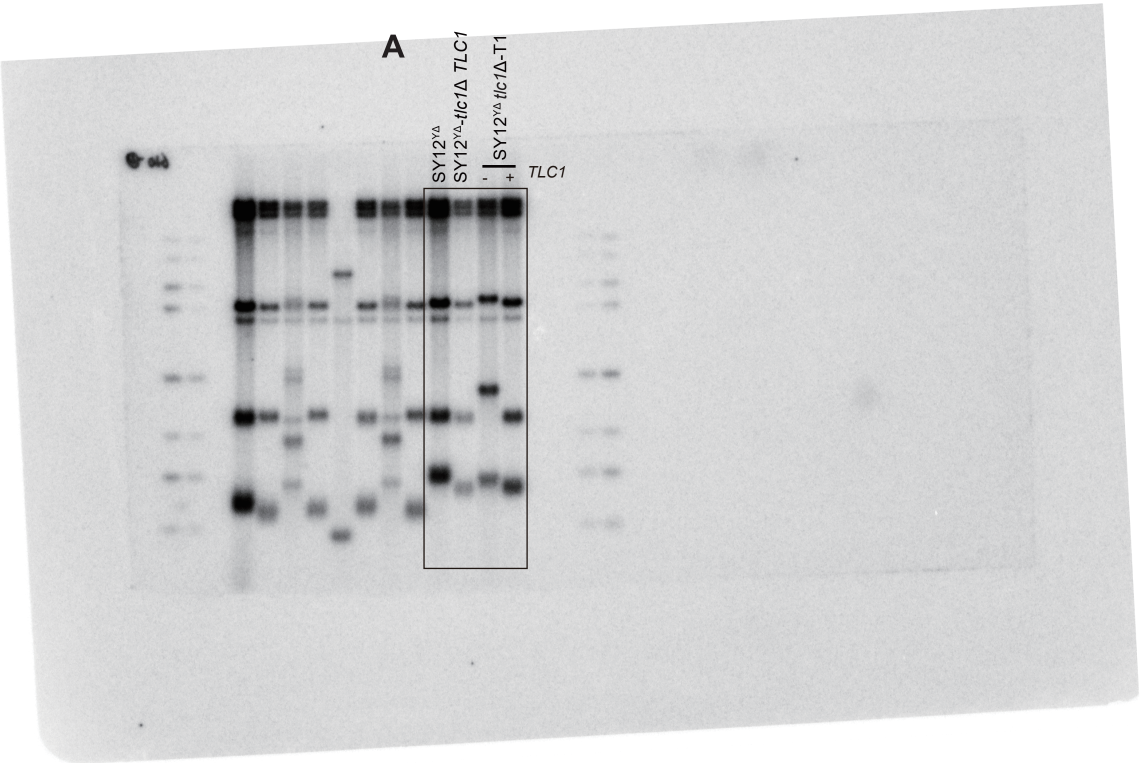
A
SY12$^{Y\Delta}$
SY12$^{Y\Delta}$-*tlc1*Δ *TLC1*
SY12$^{Y\Delta}$ *tlc1*Δ-T1
-
+
TLC1

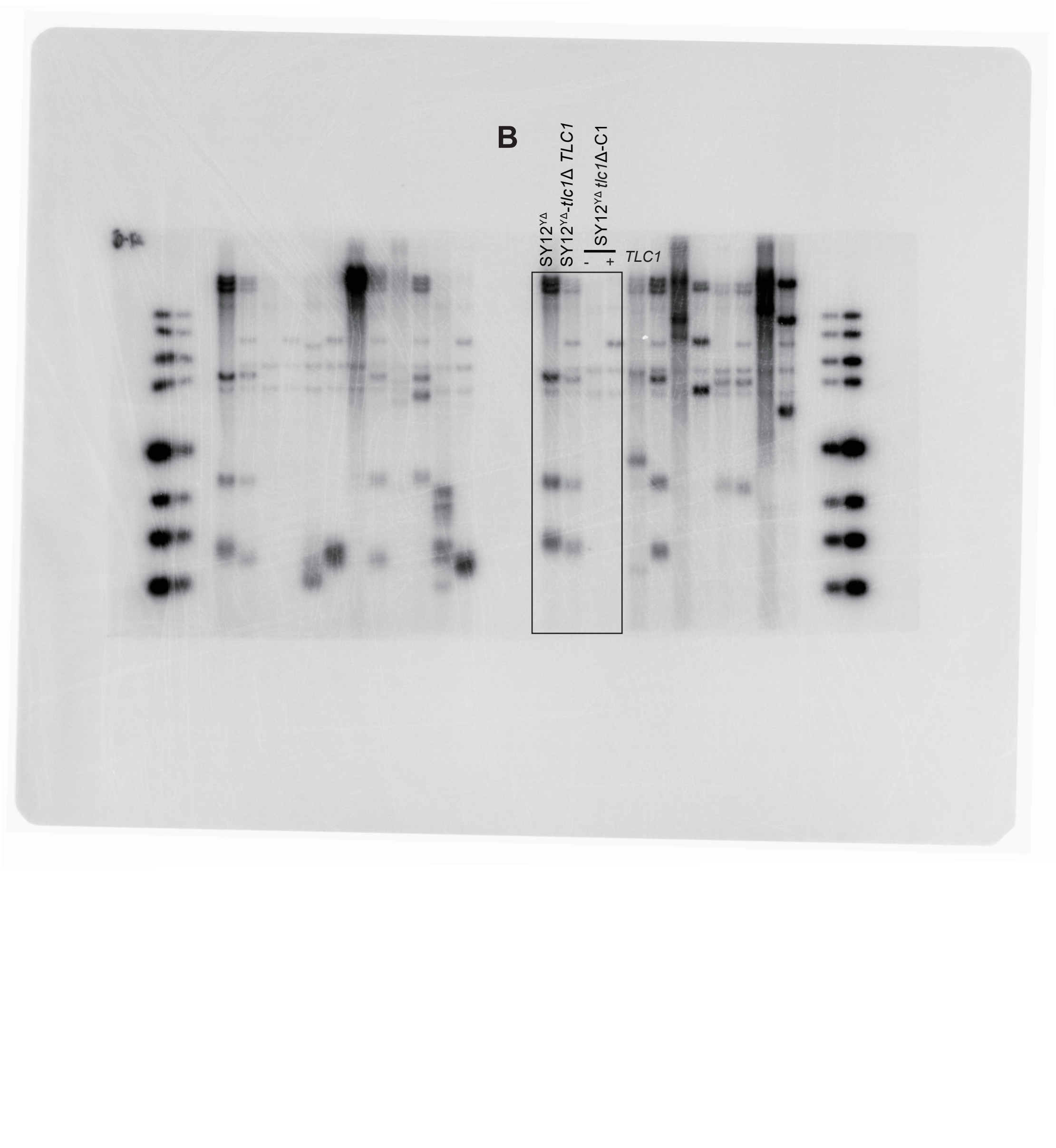
B
SY12$^{Y\Delta}$
SY12$^{Y\Delta}$-*tlc1*Δ *TLC1*
SY12$^{Y\Delta}$ *tlc1*Δ-C1
-
+
TLC1
