# Supplementary material for: Elimination of subtelomeric repeat sequences exerts little effect on telomere essential functions in Saccharomyces cerevisiae
Source: eLife. 2024 Apr 24;12:RP91223. doi: 10.7554/eLife.91223 (PMC11042809; doi:10.7554/eLife.91223)

B

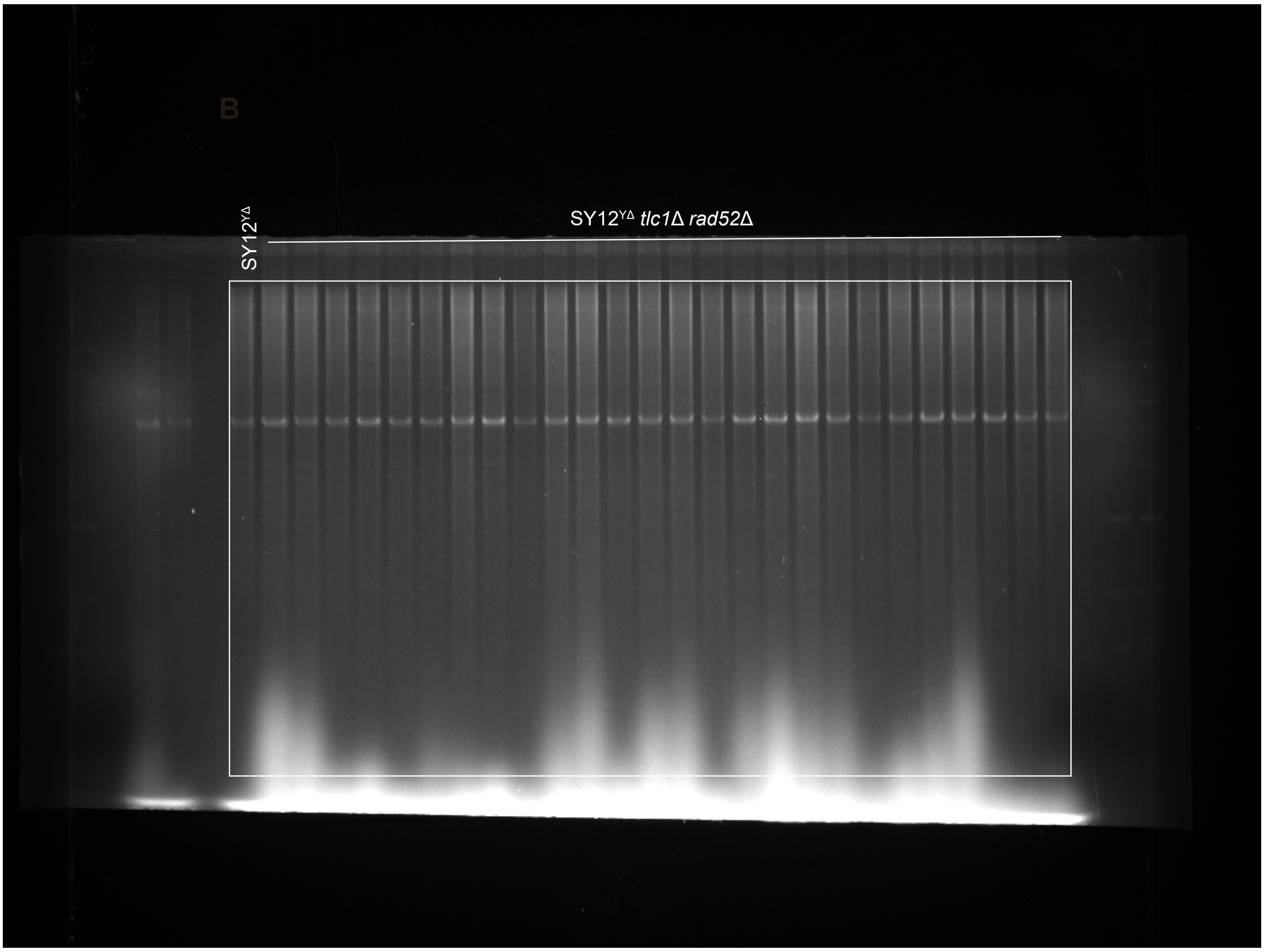

Supplement: Figure 5—figure supplement 3—source data 5. [file elife-91223-fig5-figsupp3-data5.zip › PDF containing original scans of the loading contral in Figure 5 figure supplementary 3.pdf]
