# Supplementary material for: Elimination of subtelomeric repeat sequences exerts little effect on telomere essential functions in Saccharomyces cerevisiae
Source: eLife. 2024 Apr 24;12:RP91223. doi: 10.7554/eLife.91223 (PMC11042809; doi:10.7554/eLife.91223)
Supplement: Figure 6—figure supplement 5—source data 6. [file elife-91223-fig6-figsupp5-data6.zip › PDF containing original scans of the loading contral in Figure 6 figure supplementary 5.pdf]

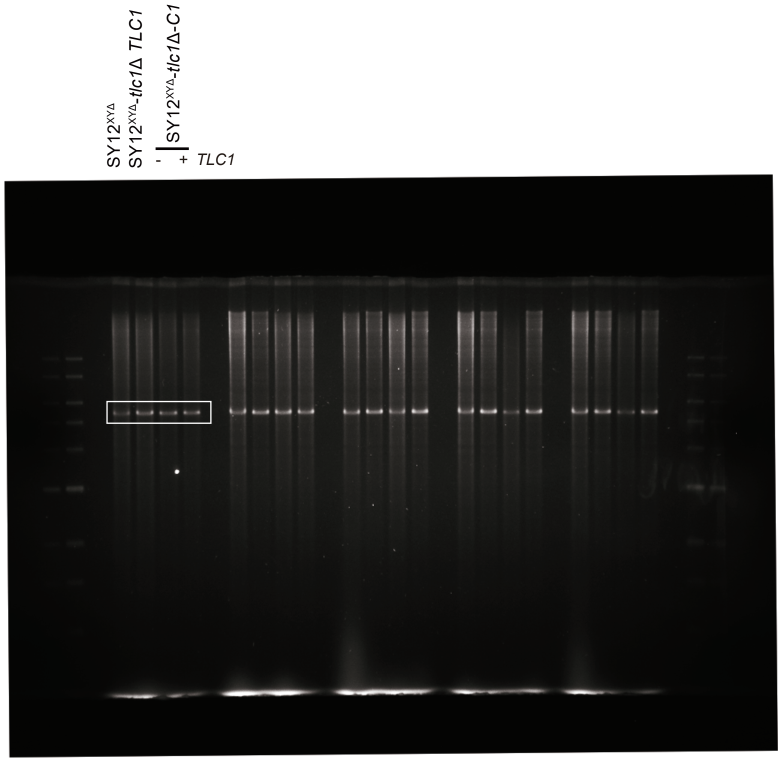
SY12$^{XY\Delta}$
SY12$^{XY\Delta}$-*tlc1Δ TLC1*
SY12$^{XY\Delta}$-*tlc1Δ-C1*
- + *TLC1*

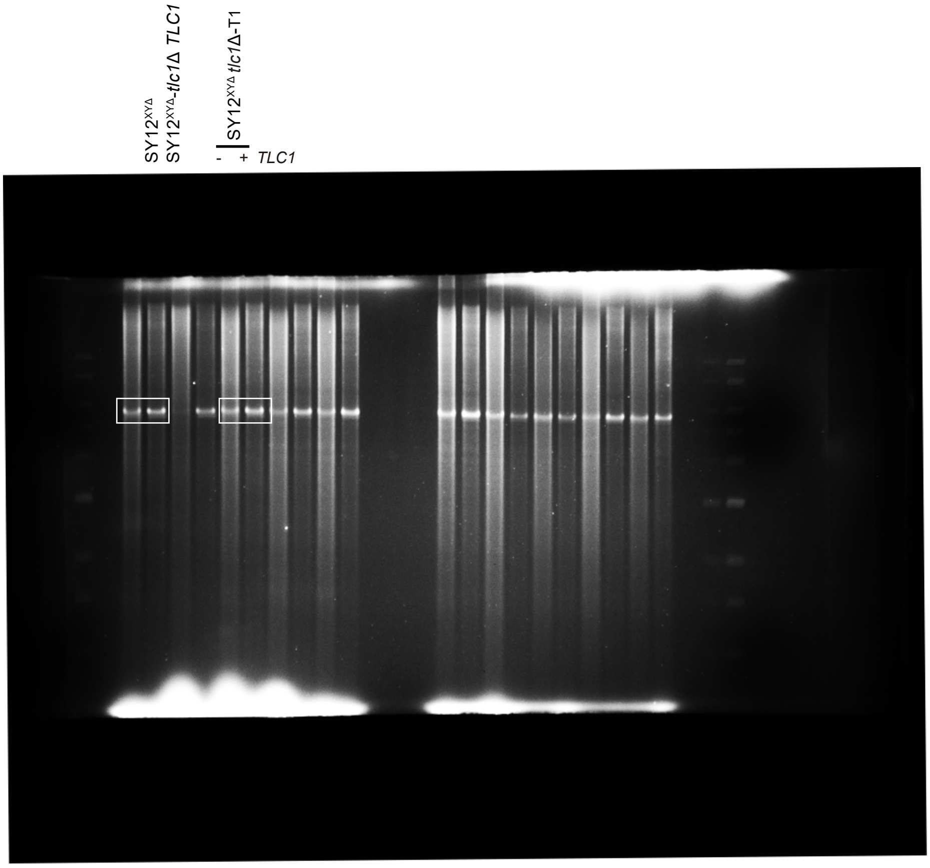
SY12$^{XY\Delta}$
SY12$^{XY\Delta}$-*tlc1Δ TLC1*
SY12$^{XY\Delta}$ *tlc1Δ*-T1
- + *TLC1*
