# Supplementary material for: Elimination of subtelomeric repeat sequences exerts little effect on telomere essential functions in Saccharomyces cerevisiae
Source: eLife. 2024 Apr 24;12:RP91223. doi: 10.7554/eLife.91223 (PMC11042809; doi:10.7554/eLife.91223)
Supplement: Supplementary file 5. [file elife-91223-supp5.docx]

Supplementary file 5. Quantitation of each survivor type in SY12 subtelomeric strains

| strain | I survivor | II survivor | Type X survivor | Uncharacterized survivor | Circular survivor | Total |
| --- | --- | --- | --- | --- | --- | --- |
| SY12 *tlc1*Δ | 8 | 1 | 12 | 19 | 10 | 50 |
| SY12^YΔ^ *tlc1*Δ | 0 | 2 | 0 | 22 | 26 | 50 |
| SY12^XYΔ^ *tlc1*Δ | 0 | 4 | 0 | 22 | 24 | 50 |
| SY12^XYΔ+Y^ *tlc1*Δ | 0 | 7 | 0 | 21 | 22 | 50 |
| SY12^XYΔ^ *tlc1*Δ *rad51*Δ | 0 | 8 | 0 | 8 | 9 | 25 |
