# Supplementary material for: Elimination of subtelomeric repeat sequences exerts little effect on telomere essential functions in Saccharomyces cerevisiae
Source: eLife. 2024 Apr 24;12:RP91223. doi: 10.7554/eLife.91223 (PMC11042809; doi:10.7554/eLife.91223)
Supplement: Supplementary file 6. [file elife-91223-supp6.docx]

| Supplementary file 6. Yeast strains used in this study | | |
| --- | --- | --- |
| Strain | Genotype | Source |
| BY4742 | *MATα his3*Δ*1 leu2*Δ*0 lys2*Δ*0 ura3*Δ*0* | Euroscarf |
| SY12 | *MATα his3*Δ*1 leu2*Δ*0 lys2*Δ*0 ura3*Δ*0* | (Shao et al., 2018) |
| WZJ0044 | BY4742 *tlc1*Δ::*HIS3/CEN* pRS316-*TLC1* | This study |
| WZJ0045 | SY1 *tlc1*Δ:: *HIS3/CEN* pRS316-*TLC1* | This study |
| WZJ0046 | SY3 *tlc1*Δ::*HIS3/CEN* pRS316-*TLC1* | This study |
| WZJ0047 | SY5 *tlc1*Δ::*HIS3/CEN* pRS316-*TLC1* | This study |
| WZJ0048 | SY7 *tlc1*Δ::*HIS3/CEN* pRS316-*TLC1* | This study |
| WZJ0049 | SY8 *tlc1*Δ::*HIS3/CEN* pRS316-*TLC1* | This study |
| WZJ0050 | SY9 *tlc1*Δ::*HIS3/CEN* pRS316-*TLC1* | This study |
| WZJ0051 | SY10 *tlc1*Δ::*HIS3/CEN* pRS316-*TLC1* | This study |
| WZJ0052 | SY11 *tlc1*Δ::*HIS3/CEN* pRS316-*TLC1* | This study |
| WZJ0053 | SY12 *tlc1*Δ::*HIS3/CEN* pRS316-*TLC1* | This study |
| WZJ0054 | SY12^YΔ^ | This study |
| WZJ0055 | SY12^YΔ+1XΔ^ | This study |
| WZJ0056 | SY12^YΔ+2XΔ^ | This study |
| WZJ0057 | SY12^YΔ+3XΔ^ | This study |
| WZJ0058 | SY12^YΔ+4XΔ^ | This study |
| WZJ0059 | SY12^XYΔ^ | This study |
| WZJ0060 | SY12^XYΔ+Y^ | This study |
| WZJ0061 | BY4742 *sir2*Δ::*HIS3* | This study |
| WZJ0062 | SY12 *sir2*Δ::*HIS3* | This study |
| WZJ0063 | SY12^YΔ^ *sir2*Δ::*HIS3* | This study |
| WZJ0064 | SY12^XYΔ^ *sir2*Δ::*HIS3* | This study |
| WZJ0065 | SY12^XYΔ+Y^ *sir2*Δ::*HIS3* | This study |
| WZJ0066 | SY12^YΔ^ *tlc1*Δ::*HIS3/CEN* pRS316-*TLC1* | This study |
| WZJ0067 | SY12^XYΔ^ *tlc1*Δ::*HIS3/CEN* pRS316-*TLC1* | This study |
| WZJ0068 | SY12^XYΔ+Y^ *tlc1*Δ::*HIS3/CEN* pRS316-*TLC1* | This study |
| WZJ0069 | SY12 *tlc1*Δ::*HIS3 yku70Δ::LEU2/CEN* pRS316-*TLC1* | This study |
| WZJ0070 | SY12^YΔ^ *tlc1*Δ::*HIS3 yku70Δ::LEU2/CEN* pRS316-*TLC1* | This study |
| WZJ0071 | SY12^XYΔ^ *tlc1*Δ::*HIS3 yku70Δ::LEU2/CEN* pRS316-*TLC1* | This study |
| WZJ0072 | SY12^XYΔ+Y^ *tlc1*Δ::*HIS3 yku70Δ::LEU2/CEN* pRS316-*TLC1* | This study |
| WZJ0073 | SY12 *tlc1*Δ::*HIS3 rad51Δ::LEU2/CEN* pRS316-*TLC1* | This study |
| WZJ0074 | SY12 *tlc1*Δ::*HIS3 rad52Δ::LEU2/CEN* pRS316-*TLC1* | This study |
| WZJ0075 | SY12^YΔ^ *tlc1*Δ::*HIS3 rad52Δ::LEU2/CEN* pRS316-*TLC1* | This study |
| WZJ0076 | SY12^XYΔ^ *tlc1*Δ::*HIS3 rad52Δ::LEU2/CEN* pRS316-*TLC1* | This study |
| WZJ0077 | SY12^XYΔ+Y^ *tlc1*Δ::*HIS3 rad52Δ::LEU2/CEN* pRS316-*TLC1* | This study |
| WZJ0078 | SY12XYΔ tlc1Δ::HIS3 rad50Δ::LEU2/CEN pRS316-TLC1 | This study |
| WZJ0079 | SY12XYΔ tlc1Δ::HIS3 rad51Δ::LEU2/CEN pRS316-TLC1 | This study |

**References**

Shao Y, Lu N, Wu Z, Cai C, Wang S, Zhang LL, Zhou F, Xiao S, Liu L, Zeng X, Zheng H, Yang C, Zhao Z, Zhao G, Zhou JQ, Xue X, Qin Z (2018) Creating a functional single-chromosome yeast. *Nature* 560: 331-335
