# Supplementary material for: Elimination of subtelomeric repeat sequences exerts little effect on telomere essential functions in Saccharomyces cerevisiae
Source: eLife. 2024 Apr 24;12:RP91223. doi: 10.7554/eLife.91223 (PMC11042809; doi:10.7554/eLife.91223)
Supplement: Figure 6—source data 5. [file PDF containing Figure 6A and original scans of the relevant Southern blot analysis.pdf › PDF containing Figure 6A and original scans of the relevant Southern blot analysis.pdf]

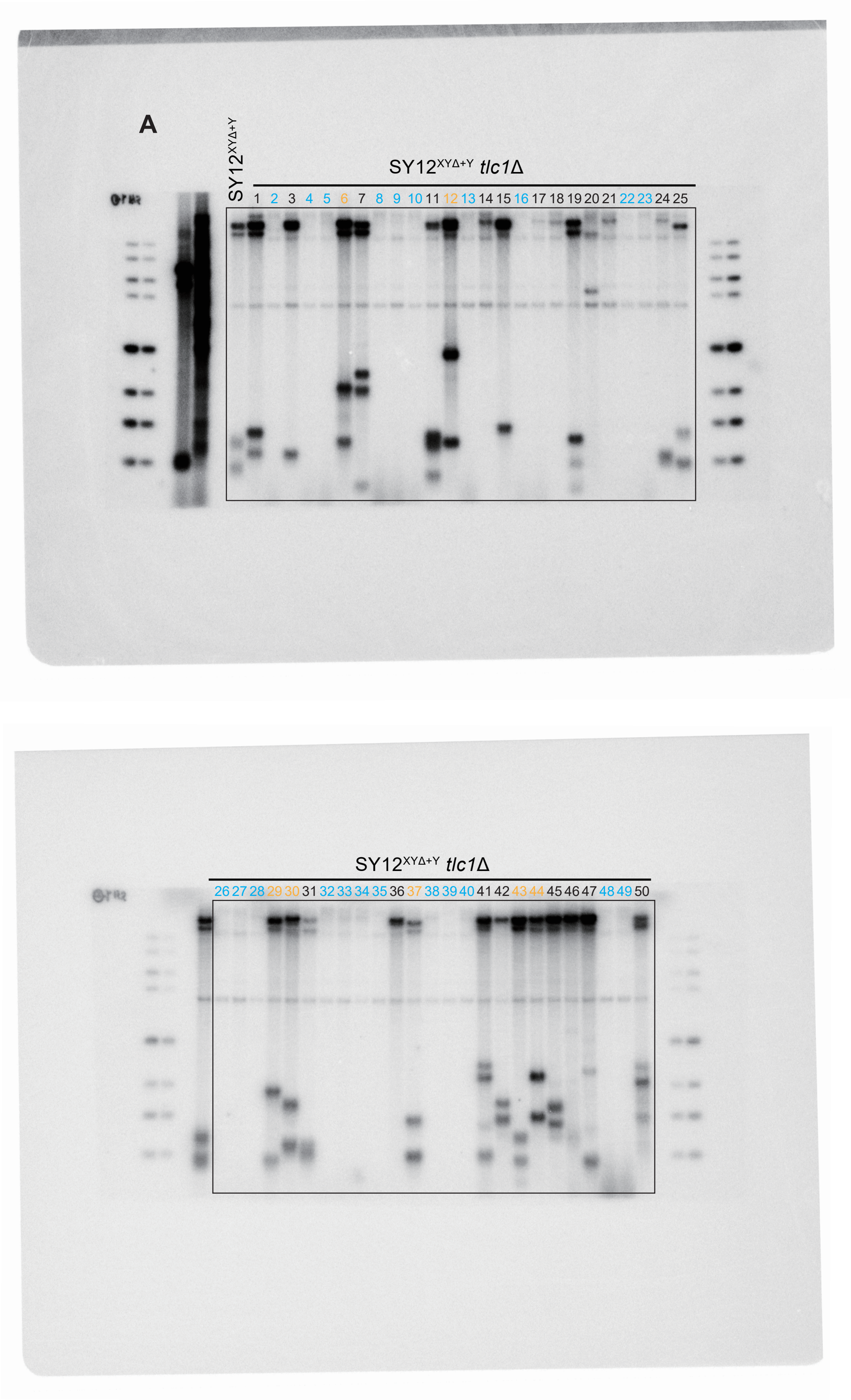
A
SY12XYΔ+Y
SY12XYΔ+Y tlc1Δ
1 2 3 4 5 6 7 8 9 10 11 12 13 14 15 16 17 18 19 20 21 22 23 24 25
SY12XYΔ+Y tlc1Δ
26 27 28 29 30 31 32 33 34 35 36 37 38 39 40 41 42 43 44 45 46 47 48 49 50
